# From Dynamic Expression Patterns to Boundary Formation in the Presomitic Mesoderm

**DOI:** 10.1371/journal.pcbi.1002586

**Published:** 2012-06-28

**Authors:** Hendrik B. Tiedemann, Elida Schneltzer, Stefan Zeiser, Bastian Hoesel, Johannes Beckers, Gerhard K. H. Przemeck, Martin Hrabě de Angelis

**Affiliations:** 1 Institute of Experimental Genetics, Helmholtz Zentrum München - German Research Center for Environmental Health, Neuherberg, Germany; 2 Kinesis Pharma BV, NK Breda, The Netherlands; 3 Technische Universitaet Muenchen, Center of Life and Food Sciences Weihenstephan, Chair of Experimental Genetics, Freising, Germany; University of Michigan, United States of America

## Abstract

The segmentation of the vertebrate body is laid down during early embryogenesis. The formation of signaling gradients, the periodic expression of genes of the Notch-, Fgf- and Wnt-pathways and their interplay in the unsegmented presomitic mesoderm (PSM) precedes the rhythmic budding of nascent somites at its anterior end, which later develops into epithelialized structures, the somites. Although many *in silico* models describing partial aspects of somitogenesis already exist, simulations of a complete causal chain from gene expression in the growth zone via the interaction of multiple cells to segmentation are rare. Here, we present an enhanced gene regulatory network (GRN) for mice in a simulation program that models the growing PSM by many virtual cells and integrates WNT3A and FGF8 gradient formation, periodic gene expression and Delta/Notch signaling. Assuming *Hes7* as core of the somitogenesis clock and LFNG as modulator, we postulate a negative feedback of HES7 on *Dll1* leading to an oscillating *Dll1* expression as seen *in vivo*. Furthermore, we are able to simulate the experimentally observed wave of activated NOTCH (NICD) as a result of the interactions in the GRN. We esteem our model as robust for a wide range of parameter values with the Hes7 mRNA and protein decays exerting a strong influence on the core oscillator. Moreover, our model predicts interference between *Hes1* and HES7 oscillators when their intrinsic frequencies differ. In conclusion, we have built a comprehensive model of somitogenesis with HES7 as core oscillator that is able to reproduce many experimentally observed data in mice.

## Author Summary

Somitogenesis is a process in embryonic development establishing the segmentation of the vertebrate body by the periodic separation of small balls of epithelialized cells called somites from a growing mesenchymal tissue, the presomitic mesoderm (PSM). The basic mechanisms are often discussed in terms of the clock-and-wave-front model, which was proposed already in 1976. Candidate genes for this model were found only in the last fifteen years with the cyclically expressed *Hairy/Hes* genes functioning as the clock and posteriorly expressed *Fgf*, *Tbx6*, and *Wnt* genes establishing the gradient(s). In addition, the Delta/Notch signal transduction pathway seems to be important for boundary formation between forming somites and the remaining PSM by inducing *Mesp2* expression just behind a future somitic boundary. Although many *in silico* models describing partial aspects of somitogenesis already exist, there are still conflicts regarding the mechanisms of the somitogenesis clock. Furthermore, a simulation that fully integrates clock and gradient was only recently published for chicken. Here, we propose a cell- and gene-based computer model for mammalian somitogenesis, simulating a gene regulatory network combining clock (*Hes1/7*) and gradient (Tbx6, Fgf8, Wnt3a) with Delta/Notch signaling resulting in dynamic gene expression patterns as observed *in vivo* finally leading to boundary formation.

## Introduction

Somitogenesis is an embryonic process that provides the basis for the mesodermal segmentation of the vertebrate body. Somites are derivatives of the presomitic mesoderm (PSM), a mesenchymal tissue that is formed during gastrulation and maintained by proliferation of cells in the tail bud. They are epithelial balls of cells that separate from the anterior end of the PSM to both sides of the neural tube. In mice, approximately every two hours one pair of somites is formed until proliferation in the tail bud stops and a species-specific number of somite pairs has been generated [1]. Fundamental to somitogenesis is the formation of a segmental boundary between the last formed somite and the unsegmented PSM. Before a boundary becomes morphologically visible, wave-like gene expression patterns propagate from the posterior to the anterior end of the PSM with the same periodicity as somites are formed [2].

Most prominent among these cycling genes are those involved in the Delta/Notch (D/N) pathway, like *Lfng* and the helix-loop-helix transcription factors *Hes1*, *Hes5*, *Hes7* and *Heyl*. They are induced by the NOTCH intracellular domain (NICD), which is cleaved off from the NOTCH receptor upon binding to DELTA or JAGGED ligands at adjacent cells and acts subsequently as co-transcription factor in the nucleus of NOTCH expressing cells [3]. NICD shows a cycling and wave-like expression in the PSM [4]. D/N signaling and *Hes7* oscillation are essential for somitogenesis [5], [6]. For example, loss of NOTCH1 function resulted in delayed and disorganized somitogenesis [7]. Similarly, in mice lacking the NOTCH ligand DELTA-LIKE 1 (DLL1) or the down-stream effector HES7 somites are not properly segmented and display a disrupted rostral-caudal polarity [8], [9]. In contrast, oscillating expression of *Lfng* in the posterior PSM seems to be dispensable for the formation of somites that later give rise to sacral and tail vertebrae [10], [11].

Other genes required for normal somitogenesis belong to the Fgf and Wnt/β-catenin signaling pathways. Both *Fgf8* and *Wnt3a* are transcribed in the growth zone of the tail bud but not in the more anterior region of the PSM. A slow decay of *Fgf8* mRNA leads to a graded expression of FGF8 protein levels from the posterior to the anterior end of the PSM [12]. Likewise, a posterior to anterior gradient of nuclear β-catenin is observed [13]. A third gradient of retinoic acid (RA) is established in the reverse direction and thought to suppress *Fgf8* expression [14]. Genes downstream of the Fgf pathway cycle in phase with respect to D/N oscillations, whereas genes belonging to Wnt/β-catenin signaling cycle in anti-phase [15]. Experimental manipulations of the Fgf or Wnt/β-catenin pathway also impair somite formation [13], [16], and inhibition of casein kinase 1, which is downstream of Wnt, lengthens the period of the somitogenesis clock [17].

In *Mesp2* deficient embryos, somite boundary formation is lost [18]. MESP2 induces the expression of *Epha4* [19]. In chick, the EPHA4 receptor binds to the ephrin B2 ligand on cells across the future boundary and thereby triggers furrow formation and cell epithelialization at the gap between the forming somite and the PSM [20]. *Mesp2* is expressed periodically by joint binding of NICD and the T-box transcription factor TBX6 in its promoter region resulting in a narrowing stripe of *Mesp2* expression at the anterior PSM [21]. While *Tbx6* expression is static and restricted to the PSM and is rapidly down-regulated as the somites form, NICD expression is dynamic, forming a wave moving in anterior direction through the PSM and contracting antero-posteriorly in width as it nears the anterior end [4]. Based on the promoter information for *Mesp2* and additional evidence that FGF8 suppresses posterior *Mesp2* expression, Oginuma et al. formulated a model that describes how dynamic NICD induces *Mesp2* expression patterns in the PSM [22]. Later, they simulated *Mesp2* and TBX6 expression with a system of differential equations in a computer model of a one-dimensional array of cells [23]. Other models employ a modulo function on an Fgf gradient [24] to generate the NICD wave, or a Boolean variable for NICD, which is repressed by the action of a *Hes7* oscillator with an empirically adjusted oscillation period [25].

We aim to develop an integrated model that depicts the causal chain leading from processes in the growth zone – unfortunately still incompletely known – to the dynamic gene expression patterns in the PSM wave zone and to segmentation of the PSM into somites. In particular, we are driven by the following questions:

- how is the NICD wave generated in the PSM?
- why does the NICD wave slow down, contract in antero-posterior direction, and stop finally?
- why is *Dll1* expression dynamic in the PSM? (For many years it was assumed to be static.)
- what is the role of *Lfng* in Delta-Notch-signaling?

These questions are connected to our central question: what is the core oscillator driving somitogenesis and how does it work? While this manuscript was in the review process, Hester et al. published such an integrated model for chick somitogenesis, in which repression of D/N signaling by LFNG serves as the core oscillator [26].

**Figure 1:**
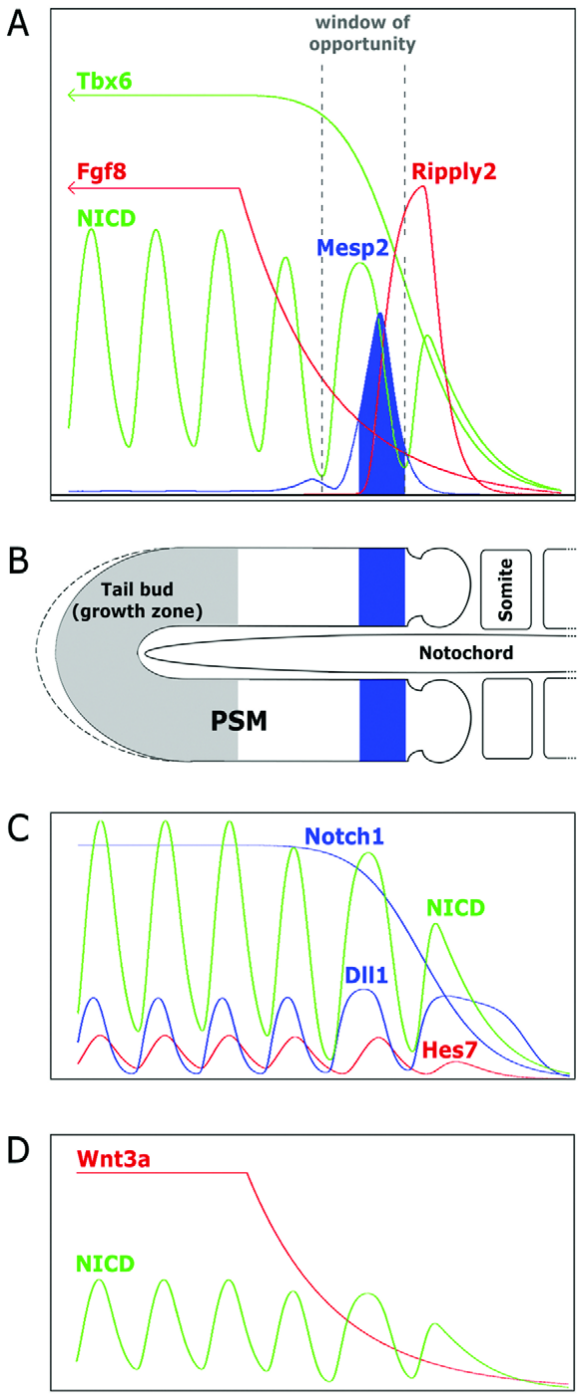
Relation between temporal evolution of gene expression and spatial pattern in somitogenesis. Panels **A**, **C**, and **D** show the time evolution of gene expressions in our model for one cell (not all drawn to scale). Panel **B** depicts a sketch of the PSM (anterior to the right) with formed somites, the growth zone, and a forming somitic cleft, in which the anterior boundary of *Mesp2* expression marks the upcoming somite boundary. (**A**) *Mesp2* is induced by dynamic NICD expression in concert with TBX6. The *Mesp2* expression boundary moves to the right together with the WNT3A and FGF8 gradients that are generated by decay of their gene products after their expression has stopped outside the growth zone. At the same time, *Mesp2* is repressed by FGF8 signaling and by RIPPLY2, which is induced by MESP2. By the growing PSM the temporal expression is mapped into a spatial pattern: in a moving ‘window of opportunity’ between activating TBX6 and repressive FGF8 expression, *Mesp2* is induced when NICD is highly expressed, i.e. the NICD wave ‘moves’ into this window. (**C**) NICD expression oscillates as a result of the reaction of NOTCH1 (static expression) with DLL1, which is dynamic and controlled by the negative feedback oscillator HES7. (**D**) NICD forms a ‘wave’ because its oscillations are slowed down by interaction with the WNT3A gradient.

Here, we propose an extended theoretical gene regulatory network (GRN) for *Mesp2* oscillation that is based on a *Hes7* feedback oscillator [27] driven by dynamic NICD expression with LFNG as modulator/enhancer of D/N signaling. Additionally, we assume a negative feedback of HES7 on *Dll1* expression and hypothesize an influence of the Wnt3a signaling gradient on the decay of NICD. Figure 1 shows a schematic representation of the vertebrate segmentation process and the underlying gene/protein expression patterns that we consider to be essential. By incorporating the extended GRN in a multi-cell simulation program of the growing PSM that allows real-time observation of gene expression in thousands of virtual cells [28], we are able to model the dynamics of NICD expression and link D/N signaling to the *Mesp2*/*Ephrin* system. As result we observe dynamic wave-like expression of NICD and *Dll1 in silico* as seen *in vivo* [4], [29] as well as periodic *Mesp2* expression along the growing PSM [4]. Introducing a gene “*Epha4*” downstream of MESP2 we obtain regular formation of “*Epha4*” expression maxima. The usefulness of our *in silico* system is demonstrated by the elimination of *Hes7* or *Ripply2* from the GRN, which results in non-oscillatory NICD and *Mesp2* expression patterns moving from anterior to posterior or, in the case of absent RIPPLY2, in a double-striped *Mesp2* expression as observed similarly in respective mouse models [6], [30]–[32]. Furthermore, we show the occurrence of beat [33] in oscillating gene expression resulting from the interference of two genetic oscillators with different eigenfrequencies.

## Model

### Motivation for the model

A lot of experimental information in biology resides in pictures derived from experiments showing their results by *in situ* staining. These results lead to hypotheses formulated e.g. in the form of GRNs. In the field of somitogenesis, Gonzalez et al. [34] has built a database of all relevant experiments and examined whether the GRNs discussed so far can explain the observed results. They found gaps in our understanding and tried to fill them with hypothetical interactions. Still missing is a computational validation whether the postulated GRN can really explain observed gene expression patterns. Therefore, one would need a gene- and cell-based model of the process in question. Our model is intended as a first step in this direction.

Somitogenesis is comprised of several subprocesses such as the growth of the PSM, oscillatory gene expression, synchronization of the oscillators, boundary formation between PSM and the next forming somite, somite polarization, and somite epithelialization. Several mathematical models exist for some of the processes, which provide a basic understanding of the described phenomena. However, each model has its own assumptions and simplifications, so it is not clear whether existing partial models are ‘consistent and integrable with one another’ [26]. Furthermore, there are experimentally generated phenotypes, which can be fully understood only by the interaction of several parts of the system, each of them modeled separately until now (for an example, see our *in silico Hes7* knock-out experiment below.)

We intended to build a comprehensive model of somitogenesis, in which most processes generated by the action of a GRN i.e. by integrating the differential equations describing the processes. However, the proliferation in the growth zone and the deactivation of *Fgf8* and *Wnt3a* expression when cells leave the growth zone were put in ‘by hand’ and are controlled by the program. We introduced EPH4A as a marker, which has the only task to trigger the shape of a cell to indicate boundary formation at the anterior PSM. This process is effected by the program when a certain threshold of EPH4A protein concentration is reached in the simulation by the GRN.

We designed our program with the intention that a user can easily change the numerical values of the rate constants, the Hill functions, or take out genes. The resulting expression pattern of a chosen gene product can be followed in real time. We therefore attached a detailed graphical user interface (GUI) to our program (see the screenshots in the user manual provided as supplementary Text S1.)

### Basic features of the model

To model gene expressions in the PSM we use essentially the same methodology as recently described in Tiedemann et al. 2007 [28], i.e. a gene- and cell-based simulation program that numerically solves differential equations describing a gene regulatory network in each cell and displays the actual concentration of a selected gene product by color intensity (virtual *in situ* staining). The following improvements were made to the program:

A growth zone of several cell layers now extends the rectangular geometry of the PSM. During growth of the PSM one cell of each column along the growth direction is randomly chosen for mitosis. Thereby the program allocates a new instance of a Java ‘cell’ object, which inherits all concentration values of its mother cell. A new ‘cell’ is created at the location of its ‘mother cell’ and then pushes stepwise all other cells of the respective column towards the posterior end of the PSM. The corresponding movements and position changes are computed by the program.

The growth zone of the PSM is defined by the program to encompass the last n layers (user defined, default value: n = 15), i.e. planes perpendicular to the growth direction. If a cell leaves the growth zone *Fgf8* and *Wnt3a* expression is shut down by the program, as we have no GRN modeling this process. The diffusion of FGF8 and WNT3A protein is not simulated in detail since we assume the gradients to be mostly determined by the intracellular decay of the respective mRNAs. So we assume a very short diffusion range for FGF8 and WNT3A [35], i.e. each cell receives only the proteins from its nearest neighbors. The concentrations are averaged and act immediately on the targets of the respective signal transduction pathway. This means, we assume the intervening processes are fast compared to the mRNA decay, which determines the dynamics and scale of the gradients.

Furthermore, we introduce distinct variables for cellular and nuclear concentrations of proteins and the respective mRNAs. The distinction in compartments is made for the oscillatory factors HES1/7, MESP2, NICD and LFNG, but not for the slow-changing concentrations of proteins and mRNAs of Notch1, Tbx6, Fgf8 and Wnt3a. For the DLL1 ligand and the NOTCH receptor we set separate variables in the cytoplasm and membrane compartments.

#### The core oscillator and D/N signaling

**Figure 2:**
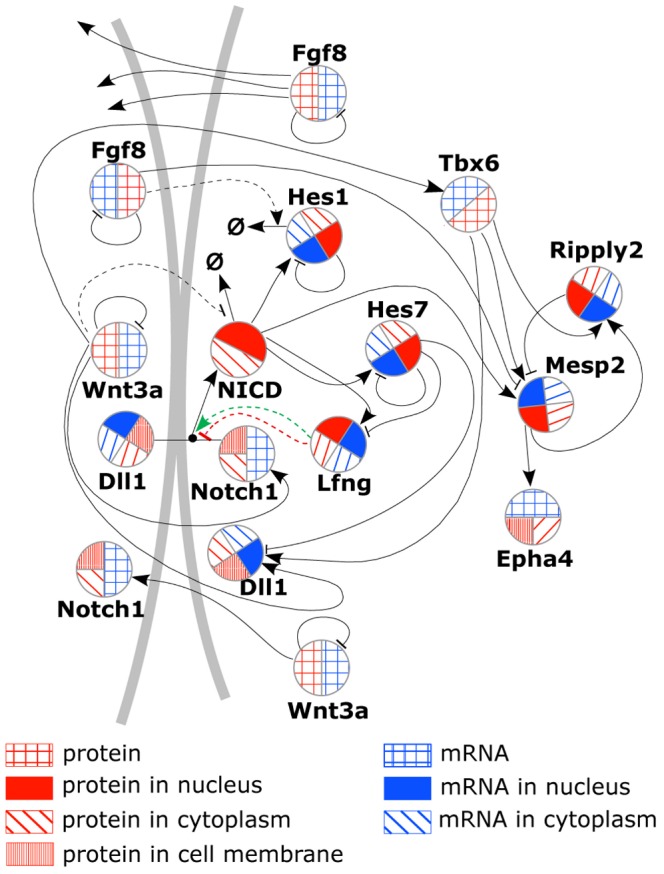
Reaction scheme of the proposed gene regulatory network (GRN). The scheme details the full GRN for one cell and part of a neighboring cell for those reactions that involve ligand-receptor interactions like in Delta-Notch signaling or input from the Fgf8 or Wnt3a signal transduction pathways. Color-coded circular areas for each gene symbolize mRNA and protein. For fast changing gene products the transport of mRNA or protein between cytoplasm and nucleus or between cytoplasm and membrane is explicitly simulated, which is indicated by dividing each half-area of the circle again. Regulatory interactions are shown as activating or repressing arrows. Broken lines indicate that the interaction is simulated only in an even more course-grained manner than the other gene regulatory reactions (see [28] for an extensive discussion). NICD, which originates through cleavage reactions following DLL1 ligand binding to the NOTCH1 receptor [3], was assigned a separate symbol to clarify that only the intracellular domain of the Notch receptor acts in the nucleus as a transcription (co)-factor. The (weak) modulating action of LFNG on D/N signaling is shown as dashed lines - (red for the case of inhibiting action, green for the case of a positive effect on the D/N reaction rate.) Arrows pointing to the symbol for the empty set designate decay reactions of a species. We suppressed them for all species' decays except for those decay rates that we assume as controlled by signal transduction pathways. This applies also to the removal of DLL1 and NOTCH1 from the membrane after their binding, resulting in NOTCH1 cleavage and NICD split-off.

A schematic view of the GRN used in our simulations is depicted in Figure 2. Its central element is the negative feedback oscillator *Hes7* [5]. By binding to the promoter it inhibits its own production. The *Hes7* promoter also receives input from D/N and Fgf signaling [36]. Furthermore, HES7 inhibits *Lfng*, which in turn modulates D/N interaction [37]. NICD acts as an activator of *Hes7* (and *Hes1*) [38]. Here, we assume that HES7 inhibits *Dll1* expression. In the following simulations, *Lfng* is induced by NICD and inhibited by HES7 but inhibits D/N signaling only marginally.

The activation of *Hes7* in the growth zone by Fgf signaling [36] is currently not included, as we have not enough promoter information for this induction of *Hes7* in the posterior tail bud, in particular of its weight relative to the influence of other promoter elements. In addition, a GRN functioning in the growth zone is still missing. The *Hes7* expression induced by FGF8 is static and we were interested in the generation of the dynamics in the wave zone of the PSM. As HES7 represses *Dll1*, which induces NICD, which in turn co-induces *Hes1/7* and *Lfng*, we expect the expression pattern in the posterior end of the tail bud to change, if one would include this. However, as one does not know how the growth zone with its expression of *Fgf8* and *Wnt3a* is maintained, there could be other influences in addition.

In the simulations presented here, D/N signaling generates the NICD wave and affects the oscillation period of the coupled oscillator system, but D/N signaling is not needed to synchronize *Hes1*/*7* oscillators in neighboring cells as all cells start synchronously in the growth zone and no source of desynchronization is introduced. All daughter cells inherit the oscillation phase of their mother cells and no stochastic noise is added in the differential equations describing the cellular processes.

For the mathematical description of the model we use ordinary differential equations. To describe negative feedback oscillators one has to introduce a function describing the repressive action of the gene product on the promoter of its gene. We use Hill functions of the form
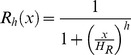
to describe this negative feedback, wherein the Hill-coefficient 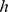 is a measure for the cooperativity of the repressor binding to the promoter and 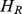 or 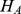 is the threshold determining half-inhibition or activation (see below). For transcription factors binding as homo-dimers we set the Hill coefficient to the value of 2 [27].

To describe activating gene action we use analogously Hill functions of the form
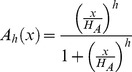
Oscillations start only when there is a delay between gene expression and negative feedback. This is often modeled with direct introduction of delayed arguments into the differential equations specifying the time used for transcribing a gene into mRNA and translating a mRNA into protein, resulting in a so-called delay differential equation system (for an example see [39], [40]).

Another way to introduce delays is to model the system with a chain of transport equations describing, for example, the intracellular transport of a chemical species as a chain of chemical reactions that changes the species in one compartment or way station into another in the next compartment. For instance, the movement of a protein from cytoplasm to the nucleus is modeled as a transformation of a cytoplasmic protein into a nuclear protein. These kind of models are also sometimes referred to as ‘Goodwin models’ [41]. However, modeling delays in this manner comes at a computational price. If one couples a negative feedback using one repressive Hill function with a chain of several transport equations with the last transported species repressing the production of the first one in the chain, one has to introduce ever larger Hill coefficients the lower the number of transport equations are. The maximum is 8 for a minimal set of three equations [42]. For smaller, more realistic values of the Hill coefficient one has to use more transport steps, implying more differential equations. Alternatively, one can introduce nonlinearities, for instance, a saturated decay process [42]–[44]. Our previous model [28] had three equations: two for the nuclear and cytoplasmic protein and one for the mRNA, thereby not differentiating between nucleus and cytoplasm. In our extended model, presented here, we abandoned the unequal treatment for protein and mRNA and implemented nuclear and cytoplasmic compartments for both protein and mRNA. To avoid large Hill coefficients we introduced a saturation of transcription factor decay in the nucleus assuming that the protein decay by nuclear proteasomes could become saturated.

In the following, we give the example for *Hes7* (we suppress the gene indices on the variables in the right side of the equations except when the variables refer to other genes).
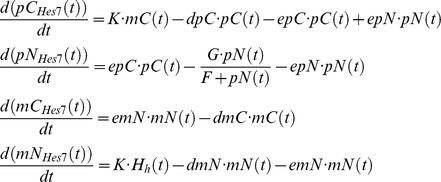
wherein 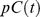, 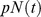, 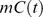, 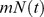 designate concentrations of cytoplasmic protein, nuclear protein, cytoplasmic mRNA, and nuclear mRNA, respectively, while 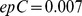, 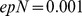, 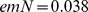 are the export rates of the protein from cytoplasm to nucleus, from nucleus to cytoplasm, and for the transport of mRNA from nucleus to cytoplasm. 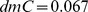, 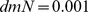, 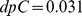 are the decay rates for cytoplasmic and nuclear mRNA, and cytoplasmic protein, respectively. We assume a very small rate of mRNA degradation in the nucleus for all genes [45]. Here and in the following, decay rates are given in 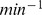 and concentration values in arbitrary units. 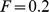 and 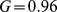 describe the saturated protein decay in the nucleus. 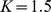 denotes the translation rate, while 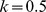 designates the maximal transcription rate.

The control of *Hes7* transcription by the Notch intracellular domain (NICD) and the negative feedback of HES7 on its own promoter is described by the Hill function 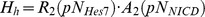 with 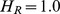 and 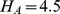.

The bHLH-transcription factors HES7 and HES1 bind as dimers to their own promoters to inhibit transcription. We chose a Hill-coefficient of 3 for *Hes1* due to the assumed concurrence of three HES1 dimer-binding sites (so-called N-boxes) in its promoter and an assumed strong cooperativity between dimers occupying these binding sites [46], and a Hill-coefficient of 2 for *Hes7* as it contains only one N-box in its promoter [47]. If HES7 binding would happen also to the so-called E-boxes in the *Hes7* promoter the Hill-coefficient might be higher [27]. However, Chen et al. have shown that HES7 only binds to the N-box [48], so only one HES7 dimer binds, leading to a Hill-coefficient of 2.

In contrast to Bernard et al. [49], we did not model the interaction with co-factors like Groucho/TLE1. We subsumed their influences in the basal transcription rate.

*Hes7* and *Hes1* are downstream of D/N signaling. It was shown that two complexes comprising NICD, MAML1 and CSL bind as a dimer to the *Hes1* promoter [38]. Therefore, we chose a Hill-coefficient of 2 for the Hill-function describing the activation of transcription by NICD.

NICD is a protein that is generated by proteases after binding of the DLL1 ligand to the NOTCH1 receptor. Subsequently, it moves from the cytoplasm to the nucleus [50].
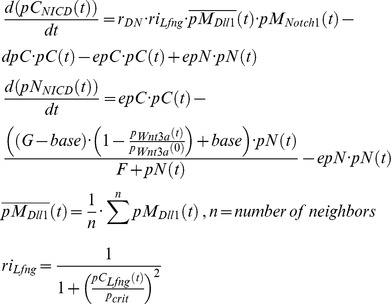



Here, 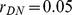 is the reaction rate between NOTCH1 receptors and the DLL1 ligands on the 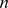 neighboring cells while 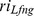 describes the inhibition of D/N signaling by LFNG. For the simulations shown here the default value of 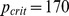 is chosen so high that 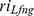 has only a small influence on D/N signaling. 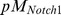 designates NOTCH membrane protein, 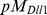 DLL1 protein in the membrane. 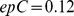 and 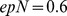 are the export rates for NICD from the cytoplasm and nucleus, 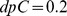 is the NICD decay rate in the cytoplasm. As NICD acts as a co-transcription factor in the nucleus its import rate to the nucleus is chosen larger as the export rate. In our model, we assume that the decay rate of NICD depends on Wnt signaling. It inhibits the decay rate in the growth zone where only a residual activity with a very low base rate (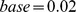) remains. With the degradation of *Wnt3a* mRNA outside of the growth zone also WNT3A protein diminishes, so that the NICD decay rate can rise to a maximum value of 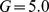.

#### Induction of *Notch1*, *Dll1* and *Tbx6* by WNT3A

In our model, *Dll1*, *Notch1*, and *Tbx6* are under the control of the Wnt signaling pathway [51]–[53]. To obtain a sharp expression boundary of *Notch1* at the anterior end of the PSM, we set a rather high Hill coefficient of 3. The expression of *Dll1* is under the control of TBX6 and WNT3A, with several binding sites for TBX6 and the Wnt effector LEF1/TCF in the *Dll1* promoter [52]. In the simulations, we use a multiplication of activating Hill functions with coefficients of 3 and 1, respectively, to model this relationship as joint action of TBX6 and WNT3A on the *Dll1* promoter, whereby WNT3A protein concentration in the Hill function is computed by averaging over neighboring cells.

We describe *Dll1* expression by a transport equation system as an oscillatory gene because we take into account that *Dll1* showed dynamic expression in the PSM.
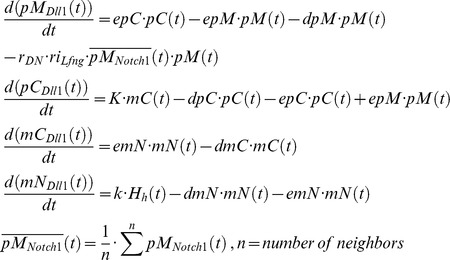



*Dll1* is activated directly and indirectly via TBX6 by Wnt signaling [52]. We assume an additional control by HES7. Therefore, we chose a Hill function of the form 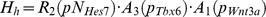, with 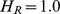, 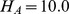 for TBX6 activation, and 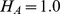 for activation by WNT3A. The rate constants are: 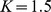, 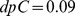, 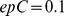, 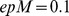, 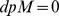, 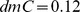, 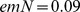, 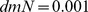 and 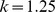.

When DLL1 in the membrane of one cell and NOTCH1 in the membrane of a neighboring cell react, the intracellular part of NOTCH1 is cleaved off to release NICD, which results in the destruction of the NOTCH1 molecule in this reaction. Consequently, the reaction term is added to the NICD equation and subtracted in the equation describing NOTCH1 in the membrane. As the DLL1 ligand, bound to the extracellular domain of NOTCH1, is endocytosed and probably degraded [54], the same reaction term is subtracted in the equation describing DLL1 in the membrane.

As we do not expect dynamic *Notch1* expression we describe its mRNA concentration by one simple equation with a production and decay term. We chose a rather high Hill coefficient of 3 to get a sharp expression boundary at the anterior end of the PSM. This has only phenomenological reasons, as experimental data are sparse.
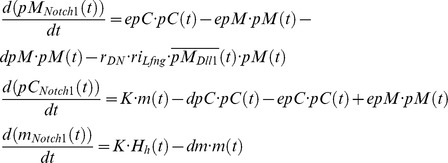


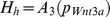
 with 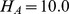. The rate constants are 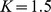, 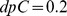, 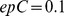, 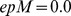, 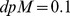, 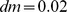, and 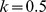.

The same assumption of non-oscillatory behavior is made for *Tbx6* expression. We chose a coarse description of *Tbx6* dynamics without differentiating between nuclear and cytoplasmic variables.
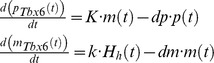


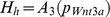
 with 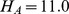. The rate constants are 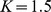, 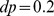, 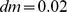, and 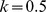. Here, we simulate the constant TBX6 protein expression in the PSM with a sharply curtailed anterior boundary as observed *in vivo* and chose a high Hill coefficient of 3, a short protein and long mRNA half-life.

As the Fgf8 and Wnt3a gradients were shown to have slow dynamics [12], [13], we use a simplified description also in these cases.
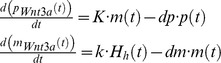


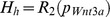
 with 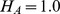. The rate constants are 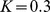, 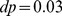, 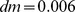, and 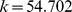 for the growth zone, and 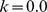 for all other cells, i.e. transcription of *Wnt3a* and *Fgf8* is shut down when a cell leaves the growth zone. The Wnt gradient is modeled analogous to the Fgf8 gradient as outlined in our previous model [28]. Due to the lack of more informative data on Wnt3a, we assume a relatively short protein half-life of 20 minutes and a mRNA half-life of 2 hours as default values comparable to the experimentally determined *Fgf8* mRNA half-life [12]. A similar decay profile for the Wnt3a gradient was suggested by Aulehla et al. [13]. With a *Wnt3a* mRNA half-life set to 2 hours a cell experiences approximately 5 oscillation periods (depending of the position of a cell in the growth zone) before becoming part of a somite, which is realistic compared to experimental results [37].

#### Action of the Wnt gradient on the core oscillator

To describe the progressive slowdown of the oscillators with increasing distance from the growth zone, we assume an influence of the Wnt3a gradient on the decay of NICD. Phosphorylation of NICD by GSK3, which is inhibited by Wnt signaling, promotes the degradation of NICD in the proteasome [55], [56]. We take this into account by mathematically inverting the Wnt3a gradient before coupling it to the NICD decay in the nuclear compartment. As a result, the decay of NICD starts at a low threshold value in the growth zone where Wnt signaling is maximal and rises to a maximum in the wave zone where Wnt signaling decays exponentially. In the schematic view of the GRN (Figure 2) this is indicated by an inhibitory action of WNT3A on the NICD decay.

#### Choice of rate constants in the core oscillator

The chosen rate constants have to fulfill several requirements: The whole oscillator including D/N signaling should oscillate with a period of approximately 120 minutes. The netto decay rates should be fitting to the decay rates measured for *Hes1* and *Hes7* [57], [58], and allow for fast synchronization of D/N coupled oscillators. Furthermore, the decay rates should be in a biological realistic range. We, therefore, have chosen very fast rates in processes involving DLL1, NOTCH1, and NICD proteins, as transport and removal processes at the cell membrane may be effected by vesicular transport processes [59].

Taken together, the chosen parameter set allows dynamic expression of *Hes7* and *Dll1* as well as enables the NICD wave as described by Morimoto et al. [4], which is implemented by the influence of Wnt signaling on the decay of nuclear NICD.

#### Induction of *Mesp2* in the anterior PSM

*Mesp2* is induced by the joint action of NICD and TBX6 and is suppressed posteriorly by FGF8 [22] and anteriorly by RIPPLY2 [31]. This complex interaction of transcription factors at the *Mesp2* promoter is modeled in a Hill function using a product of Hill functions and comprises the concentrations of NICD, TBX6, FGF8, and RIPPLY2 as arguments.
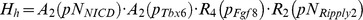



#### Repression of *Mesp2* by FGF8 in the tail bud

*Mesp2* expression is assumed to be repressed by FGF8, as its posterior border coincides with the anterior expression border of *Dusp4*, an Fgf signaling target gene [22]. Since no details on the molecular mechanism underlying this suppression are known, we use an inhibiting Hill function with a coefficient of 4 for the FGF8 input in the promoter term of *Mesp2*. Since we disregard all details of FGF8 downstream signaling, which we assume to be much faster than the *Fgf8* mRNA decay, the concentration of the FGF8 protein from neighboring cells (averaged over the number of cells) is taken as direct input for the Hill function. The Fgf8 gradient is described by differential equations of the same form as for Wnt3a with identical default coefficients (see supplementary Table S1).

#### Control by RIPPLY2

MESP2 activates the expression of *Ripply2*, which in turn represses *Mesp2* and is also connected to Wnt signaling probably via TBX6 [32], [60]–[62]. As MESP2 is a transcription factor of the bHLH type that bind as dimer, we modeled the *Ripply2* promoter structure [22] in a simplified manner by assuming an activating Hill-function with a Hill-coefficient of 2 for binding of MESP2 to the *Ripply2* promoter and an inhibiting Hill-function with the same coefficient for binding of RIPPLY2 to the *Mesp2* promoter.
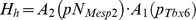
As both Mesp2 and Ripply2 have fast dynamics we use the same transport equation model as for Hes7, Hes1, and Lfng.

#### *Mesp2*, *Ripply2*, and *Epha4*

MESP2 activates the Eph receptor A4 gene *Epha4* [19]. In the simulations, we assume a long protein half-life, and when EPHA4 accumulates in a cell above a certain threshold a deformation of the cell symbolizes its epithelialization. This gives us a continuous record of past *Mesp2* expression maxima, which results in periodic boundaries.

#### Other genes driven by the core oscillator

We included the simple negative feedback oscillator *Hes1* [57] with its mRNA decay coupled to the FGF8 gradient to show the consequence of this coupling of a negative feedback oscillator and posterior-to-anterior gradient. The equations and parameters are the same as for *Hes7*, the only difference is a Hill coefficient of three in the self-repressive part of the Hill function 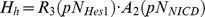.

#### The role of *Lunatic fringe*

LFNG influences the interaction of the NOTCH1 receptor with the DLL1 ligand, is activated by NICD and repressed by HES7 [48] (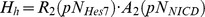) and cycles in phase with genes of the D/N pathway. Not included in our simulation is a promoter element responsible for the rostral stripe expression of *Lfng*. Therefore, the expression pattern simulated in the model presented here corresponds to the experiments performed by Oginuma et al. [23] where *Lfng* was expressed under control of the *Hes7* promoter. Due to the lack of precise data we used the same parameter values as for Hes1/7. In the simulation run with the default values for the coupling of LFNG to D/N signaling, *Lfng* expression serves only as a clock output as it influences D/N signaling only very weakly.

#### Alternative couplings of clock and gradient

Uriu et al. demonstrated in a cell- and gene-based simulation of the zebrafish PSM that several possibilities exist to couple a gradient to a system of D/N-connected negative-feedback oscillators to generate wave-like gene expression patterns [43]. We therefore examined also two alternative models: (i) coupling of the FGF8 gradient to the HES7 protein decay (Figure S1) or (ii) coupling of the FGF8 gradient to the mRNA decay of *Hes7* (Figure S2).

## Results

To model the dynamic behavior of gene expression during somitogenesis we utilized our gene-based simulation tool described above. It numerically solves a set of differential equations describing the GRN in each virtual cell and displays the concentration of a chosen gene product (mRNA or protein) by color intensity [28].

**Figure 3:**
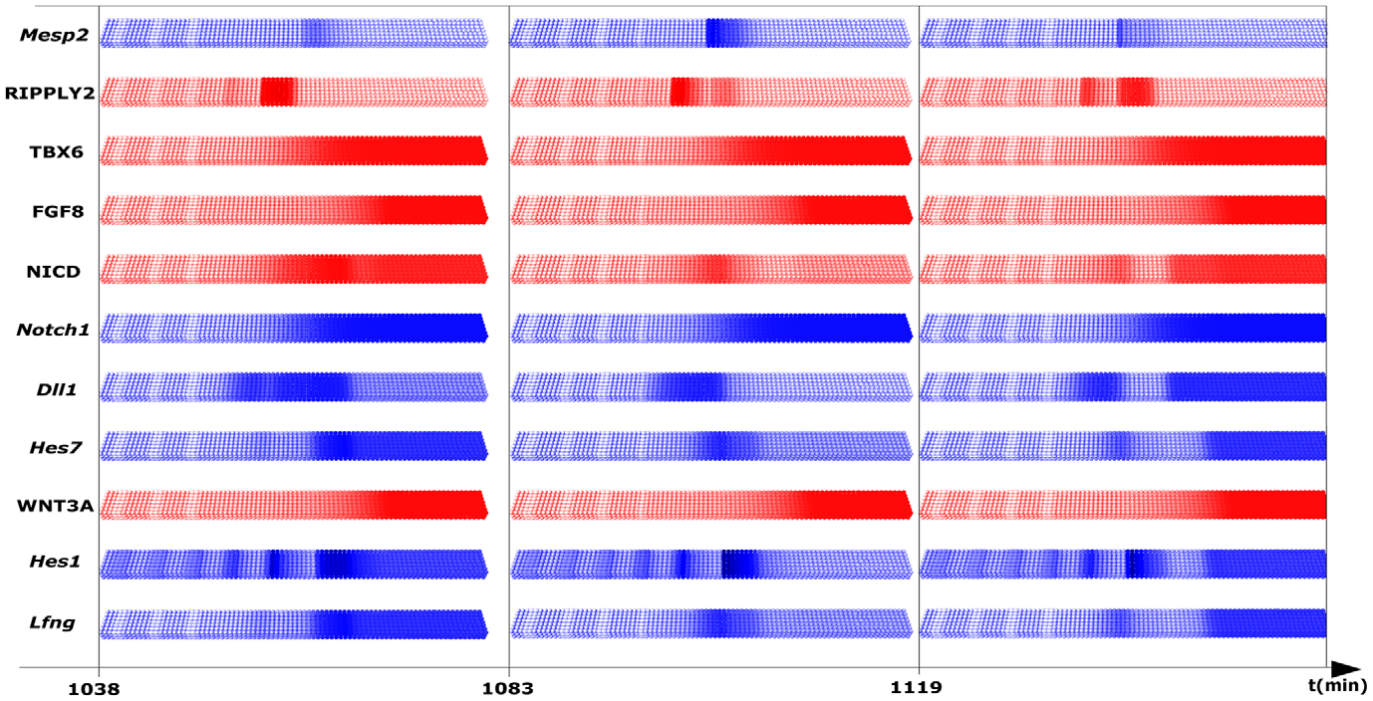
Virtual expression patterns as simulated *in silico* by the proposed gene regulatory network. Expression patterns are shown at three different time points in one oscillation cycle for one half of the PSM. Cytoplasmic mRNAs are colored in blue, proteins in red. The tail bud is growing from left to right. When EPHA4 concentration has reached a certain threshold, the virtual cells change their shape to symbolize epithelialization at the forming somite border.

Figure 3 depicts snapshots of the expression of *Mesp2*, RIPPLY2, TBX6, FGF8, NICD, *Notch1*, *Dll1*, *Hes7*, WNT3A, *Hes1* and *Lfng* at three time points within one oscillation cycle. The blue color indicates mRNA expression in the cytoplasmic compartment, while protein expression is depicted in red.

The interaction of the DLL1 ligand and the NOTCH1 receptor on neighboring cells leads to the release of NICD into the cytoplasm. Similar to the situation *in vivo* [4], we observed an NICD wave running in the posterior to anterior direction inducing a corresponding wave of *Hes7* mRNA expression (Videos S1 and S2). In the GRN, we also assumed a negative regulation of *Dll1* through HES7, which results in a dynamic expression of *Dll1* mRNA (Video S3). This simulated *Dll1* dynamics in the PSM compares well with the *in vivo* situation [29]. However, it did not reproduce *Dll1* expression in already formed somites [63] (see discussion).

We assumed half-lifes of 2 hours for the mRNAs of both *Wnt3a* and *Fgf8*, whose transcription is limited to the growth zone, and of 20 minutes for the corresponding proteins. As result, the WNT3A and FGF8 protein gradients recede with the growing posterior end of the PSM. Because *Notch1* and *Tbx6* expression depend on Wnt3a signaling one observes a similar anterior to posterior movement of their anterior expression boundaries. Also, FGF8 in the posterior end of the PSM represses *Mesp2*. Hence, there is a moving *window of opportunity* at the anterior end where *Mesp2* expression can be activated. When the NICD wave hits this part of the PSM, *Mesp2* expression is induced and then quickly decays due to the short half-life time of its mRNA and protein (Video S4). *Mesp2* appears first in a stripe of roughly one somite in length, contracts subsequently and is finally expressed in a single-cell row wide stripe.

*Lfng* is induced by NICD and suppressed by HES7 and, hence, shows also dynamic expression. However, *Lfng* expression as shown in Figure 3 has to be seen as an output, as the modulation of D/N signaling by LFNG is negligible with the couplings chosen here. Also, the model describes only the posterior expression pattern and not the most anterior expression stripe, which is driven by a separate promoter element [11] not included in our model. For *Hes1* expression, which serves as further clock output, we coupled the FGF8 gradient to the *Hes1* mRNA decay.

To sharpen *Mesp2* expression, we included a negative feedback of RIPPYL2 on *Mesp2* (Video S5). RIPPLY2 acts as a repressor of *Mesp2*, which is also apparent in the snapshots, where RIPPLY2 protein expression is complementary to *Mesp2* mRNA expression (Figure 3).

In our simulation program, we implemented the possibility to visualize a simultaneous expression of two proteins similar to double fluorescence antibody stainings. Examples are given for HES7/NICD, MESP2/NICD, and HES7/DLL1 in Figure S3.

### Validation of the model

**Figure 4:**
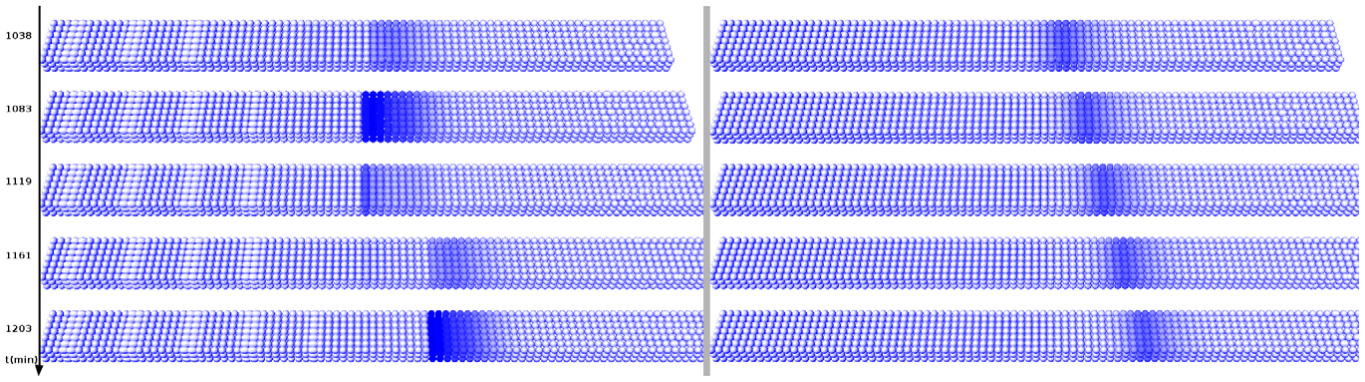
*Mesp2* expression without *Hes7*. The virtual expression patterns for *Mesp2* (cytoplasmic mRNA) are shown at five different time points in one complete and part of the following oscillation cycle. Panels on the left show the wild-type situation, panels on the right show *Mesp2* expression when *Hes7* is eliminated from the GRN (virtual *Hes7* knock-out). The tail bud of the PSM is growing from left to right.

We validated our model system by the *in silico* elimination of *Hes7*, which results in a constant, receding stripe of *Mesp2* expression moving in anterior to posterior direction with the growing PSM (Figure 4, Video S6). This result is consistent with experimentally observed data in mice deficient for *Hes7* [6], [25].

A constantly anterior to posterior moving *Mesp2* expression in the PSM can be also observed when the influence of HES7 on the *Dll1* promoter is eliminated. Although *Hes7* expression is oscillatory it shows no discernible wave in the PSM, because constant DLL1 and NOTCH1 expression result in constant production of NICD. So *Mesp2* is consequently moving within the borders set by TBX6 and FGF8 expression in the growing PSM (data not shown).

A similar *Mesp2* expression pattern was observed *in vivo* in embryos expressing NICD throughout the PSM [30]. We introduced a term for constant cytoplasmic NICD production in our simulation and observed again a constant, receding stripe of *Mesp2* expression moving in anterior to posterior direction (data not shown).

**Figure 5:**
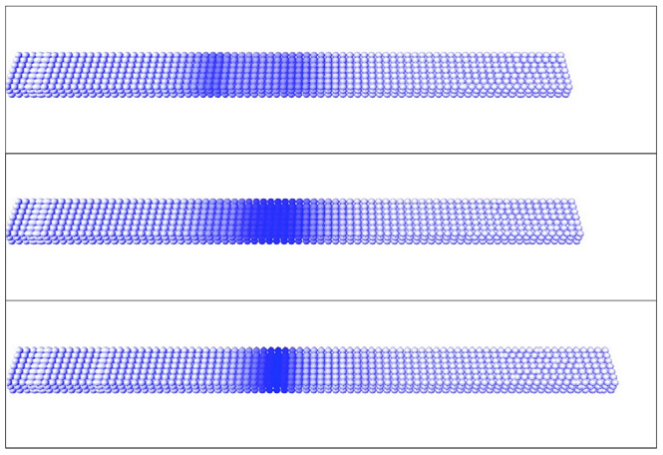
*Mesp2* expression without *Ripply2*. The virtual expression pattern for *Mesp2* (cytoplasmic mRNA) is shown at three different time points in one oscillation cycle when *Ripply2* is eliminated from the GRN. The tail bud of the PSM is growing from left to right.

When we eliminated *Ripply2* from the GRN we observed two stripes of *Mesp2* expression (Figure 5). Similar expression patterns for *Mesp2* were observed in mice deficient for *Ripply2* [31], [32].

**Figure 6:**
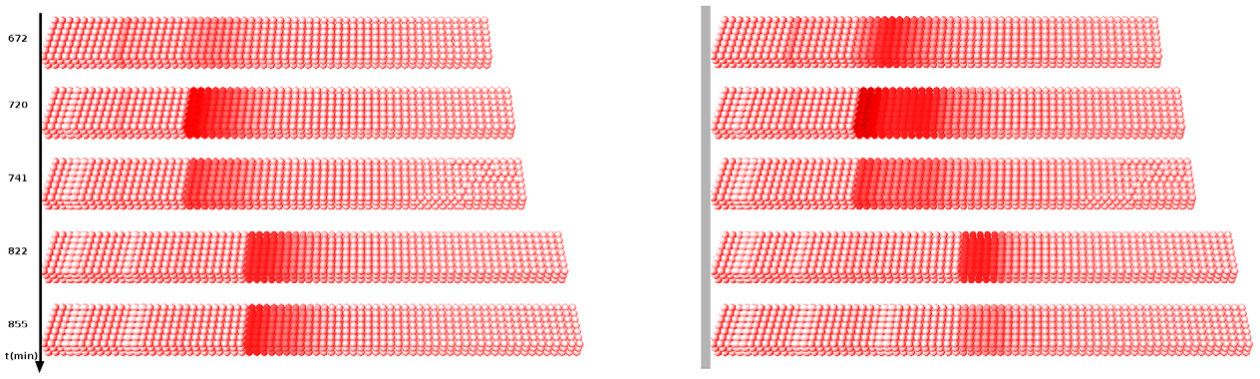
*Mesp2* expression under reduced Fgf signaling. Virtual expression patterns for *Mesp2* (cytoplasmic protein) at five different time points in one complete and part of the following oscillation cycle, when FGF8 protein production rate is reduced by 50%, 600 minutes after the simulation has been started. Panels on the left show the wild-type situation, panels on the right show *Mesp2* expression when FGF8 signaling was reduced. The tail bud of the PSM is growing from left to right.

By the usage of the chemical compound SU5402 to inhibit Fgf signaling, Niwa et al. observed a broadened stripe of *Mesp2* as result of a precociously expression in the next clock cycle [25]. To simulate an inhibition of Fgf signaling we reduced the FGF8 protein production rate during a simulation run by 50% after 600 minutes and observed a similar expanded *Mesp2* expression (Figure 6, Video S7).

### Behavior of the model under parameter variation

#### Variation of the growth rate

Changing the growth rate of the PSM in the model affects the anterior-posterior length of somites, but does not affect the patterning process itself. For instance, halving the growth rate in our model results in somites, which are approximately half as long in the axial direction. In turn, doubling the growth rate leads to somites approximately doubled in anterior-posterior length (supplemental Figure S4).

#### Variation of the clock rate

The anterior-posterior length of the somites in our model changes also when we alter parameters in the negative feedback clock of *Hes7*. There are many ways how to change the clock rate. Varying the oscillation period by changing the cytoplasmic *Hes7* mRNA decay rate and measuring the resulting variation in somite length, we observed a linear relationship (Figure S5). So it seems that the somite size is determined by the product of the clock period times the elongation velocity of the PSM.

#### Systematic exploration of parameters and robustness

We systematically explored the parameter space for the core oscillator in a 2-cell system. As D/N interaction between cells is averaged over the number of neighbors, this reduced system is appropriate to compute the dependency of oscillation frequency and amplitude on parameter variations. This system exhibits oscillations with periods, which vary in a range that is relevant for somitogenesis for a wide range of parameter choices. We show the oscillation period and maximal and minimal amplitudes for both *Hes7* and NICD in a parameter scan for each parameter of the core oscillator system (supplementary Dataset S1).

In the parameter scans one can observe:

- The oscillation period is only weakly dependent on decay rates for Dll1 and Notch1 protein and mRNA.
- Faster decay rates of Dll1 and Notch1 protein and mRNA reduce NICD amplitudes and the differences between maximum and minimum NICD amplitudes, but the HES7 amplitude and max.-min. difference is only weakly affected, as long as the minimal NICD concentration is greater than the threshold in the Hill function for *Hes7*.
- The DLL1-NOTCH1 reaction rate has almost no influence on oscillation period and amplitudes except for very low values, i.e. increasing from zero, the max.-min. amplitude difference increases fast until it becomes almost constant.
- The NICD max. amplitude rises from zero proportional to *Notch1* transcription, translation, and export-to-membrane rates, until Hill functions saturate.
- As is well known for negative feedback oscillators of the Goodwin type, the protein and mRNA degradation rates exert an extraordinary influence on the oscillation period [64]. Higher rates are leading to smaller periods as the self-repressing transcription factors are cleared faster, allowing a new round of transcription. However, the max. amplitude becomes ever smaller with increasing decay rates until the difference between max. and min. amplitude disappears, i.e. a stationary state is reached. The reverse applies for increasing transcription and translation rates: The oscillation period lengthens because the clearance of the repressor proteins takes longer (at fixed decay rate) and the oscillation amplitudes increase.
- The most important parameter scan, showing the dependence on the maximum rate of NICD degradation, is depicted in the last row: Around the value of 2 the period rises from around 120 to 150 min. As the gradient experienced by the cells when coming out of the growth zone is coupled to G_nic, this leads to an increasing value of an effective G_nic. So, the cell oscillators slow down at this distance, which is seen as ‘the wave’.

As a further test for robustness, we examined 100 parameter sets with each parameter randomly chosen from the ranges indicated in part (B) of supplemental Dataset S1 and show the distribution of the oscillation frequencies in part (C). Only one parameter set did show damped instead of undamped oscillations.

Based on these observations we consider our model as rather robust.

#### Decoupling of Hes1/7 and NICD oscillations

Increasing the mRNA decay rate of *Dll1* leads to reduced D/N signaling and, therefore, reduced induction by NICD and subsequently a reduction of the amplitude of *Hes1* mRNA expression (Figure S6). In contrast, extending the *Dll1* mRNA half-life leads to the opposite effect. Hes1 and Hes7 oscillations are slightly enhanced in amplitude and the oscillation period lengthens. However, NICD oscillation ceases and becomes a constant signal in our model. Hes1/7 oscillations continue, because their promoters are described in our model by a multiplication of an activating Hill function with NICD and a repressive Hill function with HES1/7 as inputs. This negative feedback of HES1/7 onto itself enforces *Hes1/7* oscillation under constant NICD signaling, thus decoupling Hes and NICD oscillation.

#### Interference between oscillators

**Figure 7:**
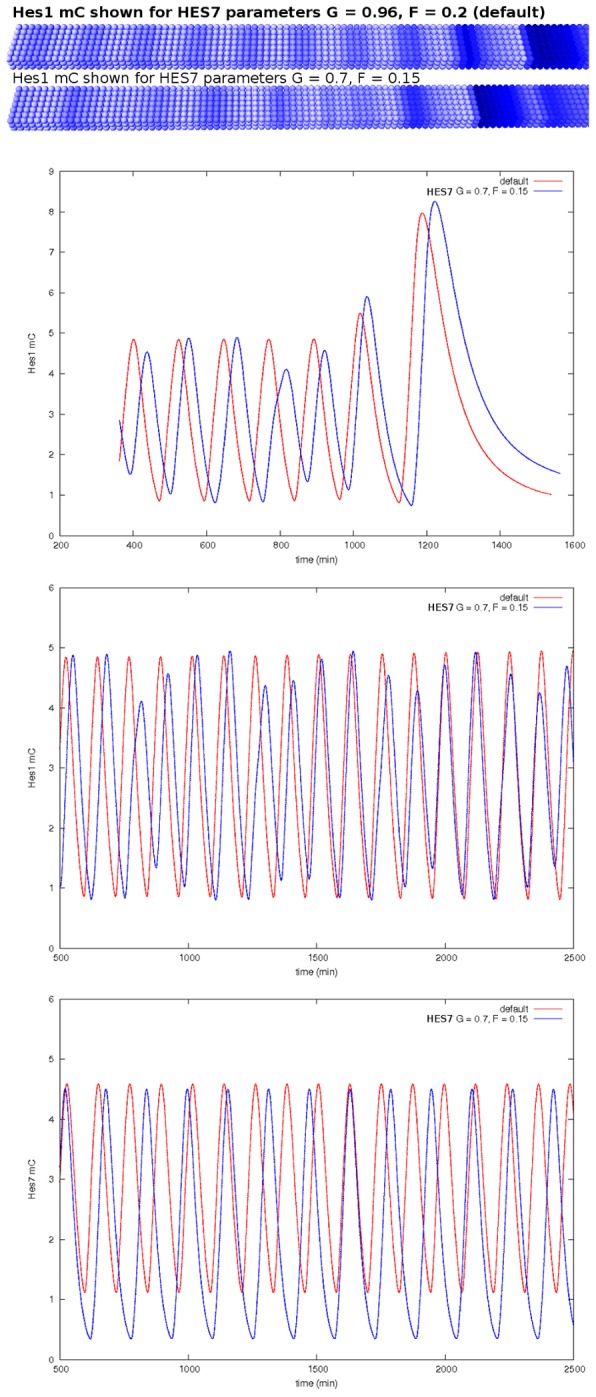
Changing the HES7 nuclear decay rate leads to a disturbance of the *Hes1* oscillator. The top panel shows the virtual expression pattern for *Hes1* (cytoplasmic mRNA) with default parameters values, the panel below shows *Hes1* expression when the nuclear decay rate of HES7 was changed resulting in the occurrence of beats, which are visualized in the uppermost concentration plot over time. The concentration plot in the middle shows the time course without the influence of the gradient decay. The plot at the bottom shows the time course of *Hes7* mRNA for comparison.

An interesting effect arises when the period of the *Hes7* oscillator is changed without altering the parameters in the *Hes1* oscillator. Periodic changes of the amplitude maxima of the *Hes1* oscillations are observed under these conditions (Figure 7). This leads to a disturbance in the somatic *Hes1* stripe pattern. To see this more clearly, we examined the effect in a simplified setting without the Wnt3a gradient limiting the number of oscillations, which a cell executes before becoming part of a somite. The observed oscillation of the maximal amplitude is reminiscent of the physical phenomenon of ‘beat’, which can be observed when two oscillators of slightly different eigenfrequencies are coupled [33]. For two harmonic oscillators of two slightly different frequencies, described by cosine functions, the result of adding both functions is a cosine function with a frequency given by the average of both original frequencies. The amplitude of the sum function is modulated by a cosine function with a frequency with half the difference of the original frequencies. However, genetic oscillators are not harmonic, so there is probably no simple formula describing the beat frequency in the case of Hes1 and Hes7 oscillations.

In our model the *Hes7* oscillation drives the oscillation of D/N signaling via inhibition of *Dll1*, and D/N signaling consequently drives the oscillation of *Hes1*. Now, there is a conflict between the NICD oscillations and the oscillation of the negative feedback of HES1 on its own promoter. Interestingly, in this way there is an effect on the expression pattern of one oscillatory gene, although the change was made to the oscillation of another gene.

As in somitogenesis many oscillators are coupled in each cell of the PSM [15], it would be interesting to search for similar effects *in vivo*. This could be responsible for very subtle effects of alterations in one component of the somitogenesis clock. However, one should check first whether this effect is still detectable in a stochastic simulation of coupled oscillators.

#### Variation of Hill coefficients

We assumed a rather large Hill coefficient of 3 for the control of *Tbx6* by Wnt signaling, reasoning that a sharp drop-off of TBX6 would be required. However, analyzing *Mesp2* expression, we saw no appreciable change in the *Mesp2* dynamics when lowering the Hill coefficient to 2 or 1 (data not shown)

The *Mesp2* promoter is described by a multiplication of activating Hill functions for TBX6 and NICD action on the promoter with Hill coefficients of 2 and inhibiting Hill functions for FGF8 and RIPPLY2 with Hill coefficients of 4 and 2, respectively. When varying the Hill coefficients in the Hill function describing the *Mesp2* promoter we observed the following: Reducing the TBX6 Hill coefficient to 1 does not change the expression pattern of *Mesp2* appreciably. Reducing the Hill coefficient describing NICD action results in a normal pattern, except that the one cell layer broad anterior-most *Mesp2* expression stripe that results from the contraction of the *Mesp2* expression in posterior-to-anterior direction, reappears for a short time after it has disappeared at the end of each *Mesp2* expression. Reducing the Hill coefficient for RIPPLY2 to 1, does not change the dynamics, except that the *Mesp2* expression in the anterior-most cell layer of the *Mesp2* expression domain does not linger longer but disappears seamlessly (data not shown).

Reducing the Hill coefficient describing repression by Fgf8 signaling down to 2, leads to a more broad initial *Mesp2* expression, and the anterior-most cell layer showing *Mesp2* expression holds the expression longer and even broadens to two cell layers. Reducing the Hill coefficients for TBX6 and NICD activation to 1 in this case, leads to a further extension of the time *Mesp2* expression remains visible in the anterior-most cell layer. Reducing the Hill coefficient for FGF8 inhibition even further down to 1 results in very broad, irregular expression of *Mesp2* in the anterior PSM, irrespective whether the Hill coefficients for NICD and TBX6 are 1 or 2 (see Video S8).

Although the computer experiments described above do not exhaust all possible combinations of Hill coefficient variations, it seems obvious that the Hill coefficient describing FGF8 inhibition has the largest impact on *Mesp2* expression.

#### Variation of the Fgf8 and Wnt3a gradients

There are several possibilities of varying parameters for the gradients. However, since the proteins in the gradients are the important downstream effectors it is sufficient to look at their behavior under parameter variation: The main effects of changing either the production rates or the decay rates are a change of the protein level and an extension or shortening of the gradient range. Changing the protein level also changes the range, if the Hill thresholds of the genes controlled by the gradients are not altered. Therefore, to isolate the effect of a pure extension of the gradient range, we changed the mRNA decay rate while fixing protein levels. As expected, a doubling of the *Fgf8* mRNA decay rate, i.e. extending the gradient range, resulted in a severe suppression of *Mesp2* dynamics, which was almost extinguished when the protein level was not adjusted. Halving of the mRNA decay rate caused a much more extended *Mesp2* expression at the anterior end of the PSM (data not shown).

In both cases the Wnt3a gradient was unchanged. More interestingly, when we doubled and quadrupled the half-lifes of both *Wnt3a* and *Fgf8* mRNA, while keeping protein levels in the growth zone at the default values, we observed a lengthening of the ‘wave zone’, which means that a cell in the PSM experiences more oscillations before it becomes incorporated into a somite (Figure S7). The expression of *Mesp2* is normal (as in Video S4). However, the anterior-most plane (or stripe, when viewed from above) of cells, which lingers for a while in the default case, lasts longer and even reappears after a while. This is even more extreme in the case of quadrupled mRNA half-lifes, where two additional planes (stripes) of cells, spaced one somite length apart and expressing *Mesp2*, can appear for a while (data not shown). Probably, the lengthened Wnt3a gradient generates a TBX6 and NICD expression that reaches further into the anterior PSM, and hence can activate an oscillation in the *Mesp2*-*Ripply2* loop. As MESP2 control of *Ripply2* is activating and RIPPLY2 inhibits *Mesp2*, a negative feedback loop is generated, which is sufficient for oscillations to occur.

#### The influence of LFNG on D/N signaling

Increased inhibition of D/N signaling through LFNG: For a further parameter variation, we abandoned our scenario of a minimal influence of LFNG on the Delta-Notch reaction and chose lower Hill-thresholds in our Hill function, which multiplies the D/N reaction rate 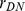 (Supplementary Table S1). As expected, the reduced reaction rate between DLL1 und NOTCH1 results in reduced NICD production, i.e. lower amplitudes in the NICD oscillations and consequently damped HES7 oscillations (Figure S8, A, B).

Enhancement of D/N signaling through LFNG: If the inhibiting factor 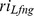 is replaced in all formulas describing D/N signaling by 
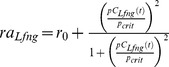
, which is an activating Hill function plus a very small constant 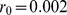 describing a residual reaction of DLL1 with NOTCH1 (unmodified by LFNG within the endoplasmatic reticulum), we observed normal HES7 and NICD oscillations. We chose 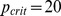. Eliminating LFNG results in damped, smaller amplitude NICD oscillations and consequently damped HES7 oscillations (Figure S8, C, D).

## Discussion

### General remarks on modeling negative-feedback oscillators

Genetic oscillators of the *Hes1/7* type are understood to result from a negative feedback with delays. These delays can be incorporated into a mathematical description in various ways. Either directly as delayed time arguments describing the duration of transcription, translation and transport processes [39], [65], resulting in delay-differential-equations, or, alternatively, as chains of transport equations [64], possibly enhanced by the introduction of nonlinearities like Michaelis-Menten or Hill-type functions [42] describing, for example, saturated decay processes. A third possibility is the explicit modeling of intracellular diffusion of proteins and mRNA in the cytoplasm of a cell [66].

Although delay models require only two equations per gene and the delays, for instance caused by splicing of *Hes7* introns [65], are easier to measure than in our compartment model, we think that delay equations used so far have two drawbacks. First, within delays many steps in gene-expression processes are hidden. As probably most processes like splicing, transport between nucleus and cytoplasm, protein and mRNA decay in the eukaryotic cell are controlled by signal transduction pathways, several modes of cross-talk [67] are neglected. Of course, it is not meaningful to model each step in transcription and translation by one differential equation. The transport steps and the saturated decay implemented in our model should be understood as representative for all these cellular processes. So, a cross-over model restricting the delays to transcription and translation would probably be the best model for future simulations.

Second, our model uses nonlinearities and saturation functions to consider proteasome-effected degradation of transcription factors. Proteasomes are located in the cytoplasm as well as in the nucleus [68], [69]. We introduce nonlinearities to allow a possible saturation of the proteasome machinery in the nucleus but neglect this possibility in the cytoplasm. However, if one simulates explicitly phosphorylation and ubiquitination of a protein before its destruction in the proteasome [70], one could assume saturated decay also in the cytoplasm [43]. For further information concerning different processes involving NICD in the nucleus and cytoplasm see [71], [72]. Because degradation processes in an eukaryotic cell are affected by protein complexes, i.e. molecular machines like proteasomes for protein disposal or exosomes for mRNA destruction, nonlinear descriptions could be appropriate for other processes as well. Every machine has a saturation threshold that can be overwhelmed by substrate molecules when their concentration is too high.

Another important issue, which needs more effort, is the role of transcription cofactors and their influence on the Hill coefficients of associated transcription factors. The higher the Hill coefficient and the degree of cooperation, the higher is the propensity to oscillations [42].

### What drives oscillatory gene expression in somitogenesis?

Many genes were found to oscillate in the PSM of mouse embryos [15], most of them are downstream of the Fgf, Wnt and D/N –pathways. Genes downstream of the Fgf pathway cycle with respect to D/N oscillations, whereas genes belonging to Wnt signaling cycle in anti-phase. Some of the downstream genes in both pathways act as inhibitors along the signal transduction cascade, forming negative feedback loops and allow for oscillations. Two scenarios are conceivable: an oscillator in one pathway acts as master clock and controls the others, or alternatively, no master clock exists and all cycling genes are equally important in the oscillatory network. A model for the latter case was developed by Goldbeter and Pourquie [44]. Genes of all three pathways generate three oscillators, which are coupled and synchronized by genetic interactions between them. We will argue for the first scenario with the D/N pathway as the central driver. First, there is evolutionary conservation. Comparing cycling gene expression in mouse, chick, and zebrafish, Krol et al. [73] found that ‘conservation of cyclic genes for all three species is limited to orthologs of the HES/her transcriptional repressors’. For example, comparing the Wnt pathway between chick and mouse only *Axin2* oscillates in both species [73], and in anolis lizards, only the orthologs of *Hes1*, *Hes7*, *Dll1*, and *Dll3* are dynamic whereas other genes like *Lfng* are not oscillating [74].

Second, several experimental data point to the same direction. In mice, most of the FGF8 targets, which oscillate in phase with D/N cycling genes, are controlled either directly by NICD as co-transcription factor, as in the case of *Dusp6* and *Spry4*, or indirectly via HES7 like *Spry2* and *Dusp4* [6], [36], [75]. Moreover, if one considers also the activation of *Snail* and *Nrarp* by NICD [76], [77], it seems reasonable to suppose that D/N signaling controls all genes that cycle in phase to D/N oscillations [15]. D/N signaling controls also *Nkd1*, which interact with components of the Wnt pathway [78]. One of the cycling genes in this pathway is *Axin2*, which have a negative feedback on Wnt signaling and could be a critical component of a Wnt oscillator [79]. However, it was shown that *Axin2* deficient mice show no somitogenesis phenotype [80].

Of course, all these arguments do not prove conclusively that D/N is the central oscillator in mice. However, it supports the assumptions made in our model, which sets D/N signaling at the center.

### How does the D/N oscillator work?

The complex D/N oscillator consists of two interlocking negative feedback loops. On the one hand, *Lfng*, which is dynamically expressed in the PSM, modulates D/N signaling by glycosylation of the NOTCH receptor. It is induced by NICD and suppressed by HES7 and was therefore considered to be a core molecular mechanism of the somitogenesis clock [81], [82]. On the other hand, the negative feedback of Hes7, which is driven by NICD and FGF8, onto itself, represses also *Lfng* expression. There are *in silico* models that take the Lfng negative feedback loop as the core of the somitogenesis clock [26], [44], while others emphasize the role of Hes7 [25]. Niwa et al., for example, assumed a direct suppression by HES7 on NICD expression [25]. However, both models cannot explain, in our opinion, some crucial experiments.

LFNG protein is secreted from a cell to terminate its action in the secreting cell but probably not as mechanism for cell-cell communication [83]. The ‘*Lfng* only’ models cannot describe the uniformly receding *Mesp2* expression observed in the PSM of *Hes7* knock-out embryos [6], [25]. These models have to postulate a direct influence of NICD on *Dll1* expression in the same cell, otherwise an oscillation in one cell could not be transmitted to neighboring cells, i.e. there would be no information transfer between cells, which is needed for the postulated role of D/N signaling in synchronizing cellular oscillators of the PSM [84].

Furthermore, *Lfng* expression seems not to be required for proper somitogenesis in the late tail bud phase [10], [11], and *Hes7* expression is still dynamic in *Lfng* deficient mice [6]. In addition, *Lfng* shows a constant expression in zebrafish and *Medaka* [85]. Contrary to these findings, Oginuma et al. recently reported a requirement for oscillating *Lfng* expression in the posterior PSM for proper somitogenesis [23], and Niwa et al. observed a damping in the amplitude of HES7 oscillations in *Lfng* deficient compared to wild type mice [25].

A model that postulates a direct interaction of HES7 on NICD has difficulties to explain the fact that *Dll1* was found to be cycling in the PSM [29]. One would have to assume a direct interaction of NICD on *Dll1* or a longer causal chain, in which, for example, NICD controls *Nkd1*, which controls Wnt signaling that controls *Dll1*.

Here, we show that a simple model analogous to the zebrafish network is able to describe the dynamic NICD expression in the PSM as it was shown by Morimoto et al. [4].

#### Our model for the core oscillator

A central point of our model is the repressive action of HES7 on the *Dll1* promoter that induces a wave-like expression of *Dll1*, which in turn leads to the NICD wave. The wave-like expression of NICD is well known [4] and the oscillating expression of *Dll1* in mice was first described by Maruhashi et al. [29]. To synchronize *Hes* oscillators in neighboring cells we introduced a negative feedback of HES7 or HES1 on *Dll1* and observed during simulations that this coupling generates a dynamic *Dll1* expression pattern. This gave us the opportunity to connect our model of gradient-coupled oscillators to Oginuma's model of dynamic *Mesp2* expression [23]. While in zebrafish a negative feedback of *her* genes on *deltaC* is well described [86], evidence in mouse is scarce. Recently, Kobayashi et al. examined HES1 targets in murine ES-cells and found *Dll1* among the genes with the highest score [87]. The fact that in *Hes7* deficient mice NICD expression in the PSM is static [6], [25] is in agreement with the assumption that a HES7-mediated negative feedback drives the core oscillator. However, this does not prove that HES7 and/or HES1 bind to the *Dll1* promoter *in vivo*. Alternatively, NICD expression might be directly suppressed by HES7 [25]. Further experiments are required to decide between the different hypotheses.

As mentioned above, we initially assumed a very weak modulation of D/N signaling by LFNG in our present model. When we enhanced the weak negative feedback of LFNG on D/N, we observed a damping of HES7 oscillations. This is in contradiction to the observation of Niwa et al. that HES7 oscillations are damped without *Lfng* [25]. However, if one takes LFNG not as inhibiting but as activating D/N-signaling, this provides a good description of the *Hes7* gene expression in wild type and

*Lfng* knock-out mice. That LFNG enhances the binding of NOTCH1 to DLL1 was recently shown by Hou et al. in a mammalian cell-culture system [88]. A negative impact of LFNG on D/N signaling in mice could therefore be questioned and needs more detailed work, which considers also the LFNG protein half-life and its degradation mechanism [83].

#### Gradient action on the core oscillator

A second important attribute of our model is the influence of FGF8 and WNT3A protein gradients on the mRNA and protein decays of cycling genes in the core oscillator. Which of the decay processes in the core oscillator are affected? Some information exists on the degradation of NICD and the decisive effects of the WNT3A gradient on somitogenesis [13]. In addition, Gibb et al. have recently shown that an inhibition of CSNK1, which is downstream of Wnt signaling [56] and together with GSK3 constitutes a complex that phosphorylates NICD before its ubiquitination [55], [89], changes the period of the oscillation clock [17]. We therefore included inhibition of the NICD decay in the nucleus by Wnt signaling. However, the degradation process of NICD might be more complicated like, for example, the coupling of Wnt signaling to the decay of SNAIL, which is phosphorylated by GSK3 in the nucleus, transported to the cytoplasm, phosphorylated again and then ubiquitinated and degraded in the proteasome [90].

We also investigated two alternative models in which we coupled the Fgf8 gradient either to the protein or mRNA decay of Hes7 (Figure S1, S2). The results obtained with these models are comparable to the model described above. However, the anterior expression boundary of *Mesp2* is less sharply defined when we coupled the Fgf8 gradient to the HES7 protein decay (Video S9). Furthermore, coupling the Fgf8 gradient to *Hes7* mRNA decay results in *Hes7* expression that does not reflect the *in vivo* situation but instead resembles the expression of *Hes1* (Video S10). To decide between the different models, more experimental data and more detailed modeling on the involved signal transduction pathways is needed.

While our model show dynamic *Dll1* expression in the PSM, in accordance with the *in vivo* situation [29], it cannot reproduce *Dll1* expression in already formed somites, suggesting that important information is missing. This is not unexpected, as we did not include mechanisms for the establishment [23], [24] and maintenance of somite polarity. Genes responsible for the latter process seem to be *Tbx15*, *Tbx18*, *Pax3*, and *Uncx4.1* [91]–[93], among others, forming a mutual inhibitory feedback loop (‘flip-flop’) including *Dll1*. Our current simulations consider the control of the *Dll1* promoter term only by WNT3A and TBX6. However, recent findings suggest that also integrin-linked kinase (ILK) affects the expression of *Dll1* in the rostral PSM [94], which might be of major importance and should be integrated in future simulations. The role of NOTCH2 expression in the somitic mesoderm and its interaction with DLL1 [95] remains also to be clarified. Furthermore, the *Dll3* gene, which shows uniform expression in the PSM, but rostral expression in already formed somites, and its interactions with *Hes5*, *Hes1* and *Lfng* [96], [97] have to be considered.

### Retinoic acid gradient

Retinoic acid (RA) is important for synchronizing and balancing the development of left and right halves of the PSM [98]. RA is expressed in the somites and forms an anterior to posterior gradient opposing the posterior to anterior gradient of Fgf8. The opposing action of both gradients was modeled by Goldbeter et al. [99] and results in a sharp expression cut-off at the anterior boundary of the FGF8 gradient.

Because there is not enough information about the control of RA in somites, which would allow us to incorporate RA signaling in our GRN, it is currently not included in our simulations. However, we modeled an inhibitory action of FGF8 on *Mesp2* expression with a high hill coefficient to ensure a steep drop-off at the anterior end of FGF8 expression.

Apart from these considerations, our model is intended to model somitogenesis in the tail bud phase. Cunningham et al. [100] have shown that during mouse embryogenesis from E9.5 to E13.5 *Mesp2* expression and somitogenesis was not changed in *Raldh2* knock-out mice, which are unable to produce RA in the somites. They concluded ‘that as early as E9.5 *Raldh2* is not required to limit the anterior extent of the caudal *Fgf8* expression zone.’

### Outlook

While it is known, for example, that *Dusp6* is controlled by FGF8-ERK1/2 via the Ets family of transcription factors [101], it is yet unknown which of the FGF8 downstream factors activate *Hes7* and inhibit *Mesp2* [22], [36]. Once this is known, we could extend our model by more detailed modeling of Fgf8 and Wnt3a signaling and the corresponding downstream genes, some of which show also cycling expression [15] due to the various negative feedbacks that are discussed for these signal transduction pathways [102]. For a single-cell-simulation this was done by Goldbeter and Pourquie [44], and for a 2-D simulation of chicken somitogenesis by Hester et al. [26]. Recently, Niwa et al. [25] observed that the expression of pERK and DUSP4, which are downstream of FGF8, is not constantly receding but periodically covers and frees the MESP2 expressing region of the PSM. This could be a result of the negative feedback of DUSP4, driven by HES7 oscillations, on Fgf8 signaling and be important for the control of *Mesp2* expression in the future somite [25]. Furthermore, besides refining the *Mesp2*, *Ripply2*, and *Tbx6* expression patterns by incorporating the mechanisms proposed by Takahashi et al. [24] a much more detailed modeling of the Ephrin/Eph receptor system with forward as well as backward signaling components [103] should be considered, as also other genes in this network cycle or are controlled by HES7 [36], [73].

In our simulations, all cells start synchronously in the growth zone and remain synchronized because daughter cells inherit the oscillation phase of their mother cells. So, synchronization of cellular oscillators by D/N coupling is not needed. We achieved a partial solution of the synchronization problem in an earlier version of our model with a growth zone comprising only one single cell layer. When we coupled the HES1 transcription factor as inhibitor to the *Dll1* promoter while Notch signaling activates *Hes1*, we observed similar synchronized cellular oscillations in neighboring cells as for a delay differentiation model of zebrafish [39], [86] or the *Hes7* oscillator of mouse [58]. However, we achieved synchronization only when we consider the binding of NICD as dimer [38] and when the relative phases of *Hes* oscillations in all cells generated in the one cell-layered growth zone differ not more than 25% (Figure S9). Uriu et al. have demonstrated that one has to include random cell movements into the PSM to achieve faster synchronization [104]. Such a random cell motility gradient downstream of FGF8 was recently described for chick embryos [105]. Furthermore, the assumption of D/N signaling to function exclusively through nearest neighbor communication could be reconsidered. In *Drosophila*, for example, dynamic filopodia transmit D/N signals during bristle formation [106]. Similar cellular extensions bearing Delta ligands were observed in zebrafish [107]. An even more radical deviation from the common view on D/N signaling might be considered by the finding that DLL4 incorporated in endothelial exosomes might be transferred and integrated into the cell membrane of distant cells [108].

Somitogenesis describes not only border formation, but also comprises the mesenchymal to epithelial transition in the outer layer of cells of the forming somite. *Snail* and *Zeb2* are genes that are well known to be involved in epithelial-to-mesenchymal transition (EMT) by controlling *E-cadherin* [109] but also show cyclic expression in the PSM [29], [76]. Finally, somitogenesis requires an extra-cellular matrix (ECM) of fibronectin surrounding the PSM [110]. Jülich et al. demonstrated that in zebrafish reverse signaling by EphrinB2a is sufficient to initiate Itgα5 clustering, alleviates non-cell-autonomous transinhibition, which prevents fibronectin activation within the PSM, but induces fibronectin matrix assembly along somite borders [111]. Taken together, these findings reinforce the need to include ECM and integrin signaling into the modeling of somitogenesis. However, the translation of gene expression patterns into the control of cell adhesion and cell shape, as well as the interaction with the extracellular matrix would require the development of a model for the cell skeleton and other features, which should not only be realistic enough but also computable in a simulation with thousands of cells.

### Conclusion

The influence of the NICD wave on the dynamics of *Mesp2* and *Ripply2* expression, activated by TBX6 and repressed posteriorly by FGF8, was recently simulated in several mathematical models. To reproduce NICD expression as experimentally observed the wave was generated either by a wave function [23], by a modulo function on an Fgf gradient [24], or by a Boolean variable for NICD which is repressed by the action of a *Hes7* oscillator with an empirically adjusted oscillation period [25].

Here, like in Hester et al.[26], we propose a model in which the NICD wave in the PSM results from a GRN. However, in our model of the mouse core oscillator for somitogenesis a negative feedback of the HES7 oscillator on the expression of *Dll1* leads to periodic expression of NICD, while Lfng is considered not as central part of the core oscillator but as a modulator of the *Hes7*-D/N oscillator. Its oscillation slows down because the degradation of NICD is influenced by a Wnt3a gradient in the posterior PSM. Since quantitative information on rate constants for production and degradation of mRNAs and proteins is mostly missing, we classify the genes in our model with fast or slow dynamics. However, our model is able to reproduce a great deal of experimentally observed data in mice. Encouraged by the agreement between experiment and our simulation, we hypothesize that HES7 binds to the *Dll1* promoter. To prove this hypothesis experimentalists could perform *in vivo* chromatin immunoprecipitation (ChIP) analyses according to a protocol previously described by Bessho et al. 2003 [5].

In the following, we will summarize the achievements but also the deficiencies of our model in bullet-point form:

Achievements

- Dynamic *Mesp2* and NICD expression as described in [4]
- *Mesp2* expression starts in a somite-wide segment and then contracts in posterior to anterior direction
- Dynamic *Dll1* expression as described in [29]
- NICD ‘wave’ as result of the control of the NICD decay by the Wnt3a gradient
- Expression waves stop as result of the genetic interaction and not by ‘manual intervention’, i.e. program triggered by some threshold
- *Hes1* ‘wave and stripe’ pattern as described in [112]
- Dynamic *Hes7* and *Lfng* expression in the PSM
- Correct description of *Mesp2* expression in *Hes7* knock-out mice [6], [25]
- Scaling of somite size with the growth rate of the PSM
- Scaling of somite size with the oscillation period of the core oscillator
- Prediction of beat in oscillatory gene expression due to interference effects between oscillators with different eigenfrequencies

Deficiencies

- No induction of *Hes7* by Fgf signaling in the tail bud
- No anterior most expression stripe of *Lfng*
- Incorrect expression of *Dll1* in already formed somites
- Negative feedbacks by Mesp2 on Tbx6 and Maml [113] not included
- No detailed modeling of Wnt and Fgf pathway
- Retinoic acid gradient not included
- Full Ephrin/Eph receptor system not included
- Epithelialization mediated by *Snail* and *Zeb* genes not included

However, our model can answer at least theoretically some of the questions listed in a recent review [79] concerning the nature of the somitogenesis oscillator and its interaction with the Wnt3a gradient. Furthermore, we now have a model for the generation of dynamic NICD expression in the PSM, which is also robust to our parameter variations. In addition, we can connect our model to the model of somite border formation as formulated by Oginuma et al. [22], which can explain important aspects of somitogenesis. Furthermore, our programming framework makes it easy to expand the model in future works.

Finally, we are pleading for *Hes7* as the central pacemaker of the somitogenesis clock with Lfng having only a modulatory role. This scenario can explain some observations other models cannot, and is in accordance with the evolutionary conservation of the somitogenesis clock. This plea is made not only by arguments, but also by demonstrating its viability in computer simulations. However, in the end the *in vivo* experiment has to decide whether HES7 binds to the *Dll1* promoter – as our model assumes – or not.

## Materials and Methods

The GRN is represented by 38 differential equations. The equations, the specific rate constants for the decay, production, as well as import of proteins and mRNAs for each gene are given in the supporting information (Table S1). The program is written in Java with the 3D-extension. For rendering the concentrations of the different gene products their values have to be normalized to lie in a range between zero and one. The differential equations are numerically solved with a fourth order Runge-Kutta-algorithm. Videos were made using camstudio software. The program writes all data of user selected cells to file. Plots were performed with the GNU-plot software. This is especially useful if one does not know beforehand the concentration range of a gene product that is selected to view in the simulation. The program can be downloaded at www.helmholtz-muenchen.de/en/ieg/downloads/simulation11.

As the program requires the Java 3D API, which for legal reasons cannot be packaged into the downloadable file by us, users must ensure that it is installed on their computers before trying to run the program. The source code is available upon personal request.

## Supporting Information

Dataset S1 **Parameter discussion for the core oscillator (HES7 and D/N).** (A) Influence of parameter variations on the HES7 oscillation amplitude (first column) (maximum in blue, minimum in red), HES7 oscillation period (second column), cytoplasmic NICD oscillation amplitude (third column), and NICD oscillation period (fourth column). (B) Parameter ranges in percentage (orange rectangles) out of which individual parameters were drawn from a uniform distribution. Vertical red line at 100% denotes default parameter values. Boundaries of the rectangles correspond to the minimal and maximal parameter variations. (C) Histogram: Distribution of period lengths of PN_Hes7 for 100 randomly drawn parameter configurations.
(PDF)

Figure S1 **GRN for an alternative model in which FGF8 is coupled to the HES7 protein decay.**
(TIF)

Figure S2 **GRN for an alternative model in which FGF8 is coupled to the** ***Hes7*** **mRNA decay.**
(TIF)

Figure S3 **Virtual double staining.** Snapshots at three different time points showing the double expression of cytoplasmic HES7 (green) and NICD (magenta) at the top, cytoplasmic DLL1 (green) and HES7 (magenta) in the middle, and cytoplasmic MESP2 (green) and NICD (magenta) at the bottom. As emissive colors were used, overlapping expression results in a white hue when the expression of both proteins is roughly equally strong.
(TIF)

Figure S4 **Variation of the PSM growth rate.** Doubling and halving of the growth rate of the PSM results in doubling and halving of the length of a somite. Somite length is measured from the middle of one stripe of deformed cells to the middle of the next stripe. Shown is the *Mesp2* expression.
(TIF)

Figure S5 **Variation of the clock rate by varying the HES7 decay rate.** Varying the oscillation period by changing the cytoplasmic *Hes7* mRNA decay rate and measuring the resulting variation in somite length, one observes a linear relationship between clock period and somite length, measured in cell numbers in the axial direction starting with and including the left deformed cell. The EPH4A threshold was set so high that mostly only one cell stripe deforms. To achieve clock periods smaller than the default case, not only the *Hes7* mRNA decay rate had to be rescaled but also all other parameters of the differential equations, except those occurring in a denominator, which is equivalent to a rescaling of time.
(TIF)

Figure S6 **Varying the mRNA decay rate of** ***Dll1*** **.** Increasing the *Dll1* mRNA decay rate leads to damped D/N signaling and consequently damped *Hes1* oscillations (top panels and plot in the middle). Decreasing the *Dll1* mRNA rate leads to roughly constant NICD expression (red panel). However, HES7 still oscillates due to the negative feedback on its own promoter (bottom plot).
(TIF)

Figure S7 **Number of oscillation periods for different gradient decay lengths.** Shown are time courses for *Hes7* oscillations in one cell when *Fgf8* as well as *Wnt3a* mRNA decay rates are changed, i.e. the gradient is lengthened or shortened. The number of oscillations a cell executes before becoming part of a somite depends on gradient length.
(TIF)

Figure S8 **Results** **for modulation of D/N signaling by LFNG.** Shown on the top row is the time course for cytoplasmic HES7 (left) and NICD (right) when the inhibition threshold in the Hill function reducing the D/N coupling between cells is lowered. This means, when the inhibition by LFNG is increased one observes an increasing damping of the oscillation amplitude. Shown on the bottom row is the time course for cytoplasmic HES7 (left) and NICD (right) with LFNG activating D/N signaling for two different threshold values in the activating function and the damped expression when activating LFNG action is abolished and only the very small residual rate of unmodified NOTCH1 binding to DLL1 is left.
(TIF)

Figure S9 **Snapshots of cytoplasmic** ***Hes1*** **mRNA concentration without (left panel) and with (right panel) D/N synchronization.** In this simplified model, *Hes1* is the core oscillator and its mRNA decay is coupled to the FGF8 gradient. Here, the PSM growth zone comprises only one layer. Newborn cells start their oscillations with a random phase difference (maximally 25%). Color intensity in each cell indicates high (dark) or low (light) mRNA concentration.
(TIF)

Table S1 **Parameter values and differential equations of the model.**
(PDF)

Text S1 **Mini manual for the simulation program.**
(PDF)

Video S1 **Time evolution of cytoplasmic NICD concentration for the model shown in** **Figure 2** **.**
(AVI)

Video S2 **Time evolution of cytoplasmic** ***Hes7*** **mRNA concentration for the model shown in** **Figure 2** **.**
(AVI)

Video S3 **Time evolution of cytoplasmic** ***Dll1*** **mRNA concentration for the model shown in** **Figure 2** **.**
(AVI)

Video S4 **Time evolution of cytoplasmic** ***Mesp2*** **mRNA concentration for the model with GRN shown in** **Figure 2** **.**
(AVI)

Video S5 **Time evolution of cytoplasmic RIPPLY2 protein concentration for the model shown in** **Figure 2** **.**
(AVI)

Video S6 **Time evolution of cytoplasmic** ***Mesp2*** **mRNA concentration for the model shown in Figure1 when** ***Hes7*** **is eliminated.**
(AVI)

Video S7 **Time evolution of cytoplasmic** ***Mesp2*** **mRNA concentration for the model shown in** **Figure 2** **when FGF8 production is reduced to 50%.**
(AVI)

Video S8 **Time evolution of cytoplasmic** ***Mesp2*** **mRNA concentration for the model shown in** **Figure 2** **when the Hill coefficient of FGF8 inhibition in the** ***Mesp2*** **promoter is set to 1.**
(AVI)

Video S9 **Time evolution of cytoplasmic** ***Mesp2*** **mRNA concentration for the model shown in Figure S1.**
(AVI)

Video S10 **Time evolution of cytoplasmic** ***Hes7*** **mRNA concentration for the model shown in Figure S2.**
(AVI)

## References

[1] Gomez C, Ozbudak EM, Wunderlich J, Baumann D, Lewis J (2008). Control of segment number in vertebrate embryos.. Nature.

[2] Saga Y, Takeda H (2001). The making of the somite: molecular events in vertebrate segmentation.. Nat Rev Genet.

[3] Fortini ME (2009). Notch signaling: the core pathway and its posttranslational regulation.. Dev Cell.

[4] Morimoto M, Takahashi Y, Endo M, Saga Y (2005). The Mesp2 transcription factor establishes segmental borders by suppressing Notch activity.. Nature.

[5] Bessho Y, Hirata H, Masamizu Y, Kageyama R (2003). Periodic repression by the bHLH factor Hes7 is an essential mechanism for the somite segmentation clock.. Genes Dev.

[6] Ferjentsik Z, Hayashi S, Dale JK, Bessho Y, Herreman A (2009). Notch is a critical component of the mouse somitogenesis oscillator and is essential for the formation of the somites.. PLoS Genet.

[7] Conlon RA, Reaume AG, Rossant J (1995). Notch1 is required for the coordinate segmentation of somites.. Development.

[8] Hrabe de Angelis M, McIntyre J, Gossler A (1997). Maintenance of somite borders in mice requires the Delta homologue DII1.. Nature.

[9] Bessho Y, Sakata R, Komatsu S, Shiota K, Yamada S (2001). Dynamic expression and essential functions of Hes7 in somite segmentation.. Genes Dev.

[10] Shifley ET, Vanhorn KM, Perez-Balaguer A, Franklin JD, Weinstein M (2008). Oscillatory lunatic fringe activity is crucial for segmentation of the anterior but not posterior skeleton.. Development.

[11] Stauber M, Sachidanandan C, Morgenstern C, Ish-Horowicz D (2009). Differential axial requirements for lunatic fringe and Hes7 transcription during mouse somitogenesis.. PLoS ONE.

[12] Dubrulle J, Pourquie O (2004). fgf8 mRNA decay establishes a gradient that couples axial elongation to patterning in the vertebrate embryo.. Nature.

[13] Aulehla A, Wiegraebe W, Baubet V, Wahl MB, Deng C (2008). A beta-catenin gradient links the clock and wavefront systems in mouse embryo segmentation.. Nat Cell Biol.

[14] Diez del Corral R, Olivera-Martinez I, Goriely A, Gale E, Maden M (2003). Opposing FGF and retinoid pathways control ventral neural pattern, neuronal differentiation, and segmentation during body axis extension.. Neuron.

[15] Dequeant ML, Glynn E, Gaudenz K, Wahl M, Chen J (2006). A complex oscillating network of signaling genes underlies the mouse segmentation clock.. Science.

[16] Dubrulle J, McGrew MJ, Pourquie O (2001). FGF signaling controls somite boundary position and regulates segmentation clock control of spatiotemporal Hox gene activation.. Cell.

[17] Gibb S, Zagorska A, Melton K, Tenin G, Vacca I (2009). Interfering with Wnt signalling alters the periodicity of the segmentation clock.. Dev Biol.

[18] Saga Y, Hata N, Koseki H, Taketo MM (1997). Mesp2: a novel mouse gene expressed in the presegmented mesoderm and essential for segmentation initiation.. Genes Dev.

[19] Nakajima Y, Morimoto M, Takahashi Y, Koseki H, Saga Y (2006). Identification of Epha4 enhancer required for segmental expression and the regulation by Mesp2.. Development.

[20] Watanabe T, Sato Y, Saito D, Tadokoro R, Takahashi Y (2009). EphrinB2 coordinates the formation of a morphological boundary and cell epithelialization during somite segmentation.. Proc Natl Acad Sci U S A.

[21] Yasuhiko Y, Kitajima S, Takahashi Y, Oginuma M, Kagiwada H (2008). Functional importance of evolutionally conserved Tbx6 binding sites in the presomitic mesoderm-specific enhancer of Mesp2.. Development.

[22] Oginuma M, Niwa Y, Chapman DL, Saga Y (2008). Mesp2 and Tbx6 cooperatively create periodic patterns coupled with the clock machinery during mouse somitogenesis.. Development.

[23] Oginuma M, Takahashi Y, Kitajima S, Kiso M, Kanno J (2010). The oscillation of Notch activation, but not its boundary, is required for somite border formation and rostral-caudal patterning within a somite.. Development.

[24] Takahashi J, Ohbayashi A, Oginuma M, Saito D, Mochizuki A (2010). Analysis of Ripply1/2-deficient mouse embryos reveals a mechanism underlying the rostro-caudal patterning within a somite.. Dev Biol.

[25] Niwa Y, Shimojo H, Isomura A, Gonzalez A, Miyachi H (2011). Different types of oscillations in Notch and Fgf signaling regulate the spatiotemporal periodicity of somitogenesis.. Genes Dev.

[26] Hester SD, Belmonte JM, Gens JS, Clendenon SG, Glazier JA (2011). A multi-cell, multi-scale model of vertebrate segmentation and somite formation.. PLoS Comput Biol.

[27] Zeiser S, Liebscher HV, Tiedemann H, Rubio-Aliaga I, Przemeck GK (2006). Number of active transcription factor binding sites is essential for the Hes7 oscillator.. Theor Biol Med Model.

[28] Tiedemann HB, Schneltzer E, Zeiser S, Rubio-Aliaga I, Wurst W (2007). Cell-based simulation of dynamic expression patterns in the presomitic mesoderm.. J Theor Biol.

[29] Maruhashi M, Van De Putte T, Huylebroeck D, Kondoh H, Higashi Y (2005). Involvement of SIP1 in positioning of somite boundaries in the mouse embryo.. Dev Dyn.

[30] Feller J, Schneider A, Schuster-Gossler K, Gossler A (2008). Noncyclic Notch activity in the presomitic mesoderm demonstrates uncoupling of somite compartmentalization and boundary formation.. Genes Dev.

[31] Chan T, Kondow A, Hosoya A, Hitachi K, Yukita A (2007). Ripply2 is essential for precise somite formation during mouse early development.. FEBS Lett.

[32] Morimoto M, Sasaki N, Oginuma M, Kiso M, Igarashi K (2007). The negative regulation of Mesp2 by mouse Ripply2 is required to establish the rostro-caudal patterning within a somite.. Development.

[33] Walker JD, Halliday D, Resnick R (2008). Fundamental of Physics, 8^th^ edition.

[34] Gonzalez A, Kageyama R (2010). Automatic reconstruction of the mouse segmentation network from an experimental evidence database.. Biosystems.

[35] Scholpp S, Brand M (2004). Endocytosis controls spreading and effective signaling range of Fgf8 protein.. Curr Biol.

[36] Niwa Y, Masamizu Y, Liu T, Nakayama R, Deng CX (2007). The initiation and propagation of Hes7 oscillation are cooperatively regulated by Fgf and notch signaling in the somite segmentation clock.. Dev Cell.

[37] Kageyama R, Masamizu Y, Niwa Y (2007). Oscillator mechanism of Notch pathway in the segmentation clock.. Dev Dyn.

[38] Nam Y, Sliz P, Pear WS, Aster JC, Blacklow SC (2007). Cooperative assembly of higher-order Notch complexes functions as a switch to induce transcription.. Proc Natl Acad Sci U S A.

[39] Lewis J (2003). Autoinhibition with transcriptional delay: a simple mechanism for the zebrafish somitogenesis oscillator.. Curr Biol.

[40] Monk NA (2003). Oscillatory expression of Hes1, p53, and NF-kappaB driven by transcriptional time delays.. Curr Biol.

[41] Goodwin BC (1965). Oscillatory behavior in enzymatic control processes.. Adv Enzyme Regul.

[42] Murray JD (2002). Mathematical biology, 3^rd^ ed.

[43] Uriu K, Morishita Y, Iwasa Y (2009). Traveling wave formation in vertebrate segmentation.. J Theor Biol.

[44] Goldbeter A, Pourquie O (2008). Modeling the segmentation clock as a network of coupled oscillations in the Notch, Wnt and FGF signaling pathways.. J Theor Biol.

[45] Audibert A, Weil D, Dautry F (2002). In vivo kinetics of mRNA splicing and transport in mammalian cells.. Mol Cell Biol.

[46] Takebayashi K, Sasai Y, Sakai Y, Watanabe T, Nakanishi S (1994). Structure, chromosomal locus, and promoter analysis of the gene encoding the mouse helix-loop-helix factor HES-1. Negative autoregulation through the multiple N box elements.. J Biol Chem.

[47] Bessho Y, Miyoshi G, Sakata R, Kageyama R (2001). Hes7: a bHLH-type repressor gene regulated by Notch and expressed in the presomitic mesoderm.. Genes Cells.

[48] Chen J, Kang L, Zhang N (2005). Negative feedback loop formed by Lunatic fringe and Hes7 controls their oscillatory expression during somitogenesis.. Genesis.

[49] Bernard S, Cajavec B, Pujo-Menjouet L, Mackey MC, Herzel H (2006). Modelling transcriptional feedback loops: the role of Gro/TLE1 in Hes1 oscillations.. Philos Transact A Math Phys Eng Sci.

[50] Rida PC, Le Minh N, Jiang YJ (2004). A Notch feeling of somite segmentation and beyond.. Dev Biol.

[51] Galceran J, Sustmann C, Hsu SC, Folberth S, Grosschedl R (2004). LEF1-mediated regulation of Delta-like1 links Wnt and Notch signaling in somitogenesis.. Genes Dev.

[52] Hofmann M, Schuster-Gossler K, Watabe-Rudolph M, Aulehla A, Herrmann BG (2004). WNT signaling, in synergy with T/TBX6, controls Notch signaling by regulating Dll1 expression in the presomitic mesoderm of mouse embryos.. Genes Dev.

[53] White PH, Chapman DL (2005). Dll1 is a downstream target of Tbx6 in the paraxial mesoderm.. Genesis.

[54] Le Bras S, Loyer N, Le Borgne R (2011). The multiple facets of ubiquitination in the regulation of notch signaling pathway.. Traffic.

[55] Jin YH, Kim H, Oh M, Ki H, Kim K (2009). Regulation of Notch1/NICD and Hes1 expressions by GSK-3alpha/beta.. Mol Cells.

[56] MacDonald BT, Tamai K, He X (2009). Wnt/beta-catenin signaling: components, mechanisms, and diseases.. Dev Cell.

[57] Hirata H, Yoshiura S, Ohtsuka T, Bessho Y, Harada T (2002). Oscillatory expression of the bHLH factor Hes1 regulated by a negative feedback loop.. Science.

[58] Hirata H, Bessho Y, Kokubu H, Masamizu Y, Yamada S (2004). Instability of Hes7 protein is crucial for the somite segmentation clock.. Nat Genet.

[59] Schweisguth F (2004). Regulation of notch signaling activity.. Curr Biol.

[60] Biris KK, Dunty WC, Yamaguchi TP (2007). Mouse Ripply2 is downstream of Wnt3a and is dynamically expressed during somitogenesis.. Dev Dyn.

[61] Dunty WC, Biris KK, Chalamalasetty RB, Taketo MM, Lewandoski M (2008). Wnt3a/beta-catenin signaling controls posterior body development by coordinating mesoderm formation and segmentation.. Development.

[62] Hitachi K, Danno H, Kondow A, Ohnuma K, Uchiyama H (2008). Physical interaction between Tbx6 and mespb is indispensable for the activation of bowline expression during Xenopus somitogenesis.. Biochem Biophys Res Commun.

[63] Bettenhausen B, Hrabe de Angelis M, Simon D, Guenet JL, Gossler A (1995). Transient and restricted expression during mouse embryogenesis of Dll1, a murine gene closely related to Drosophila Delta.. Development.

[64] Zeiser S, Muller J, Liebscher V (2007). Modeling the Hes1 oscillator.. J Comput Biol.

[65] Takashima Y, Ohtsuka T, Gonzalez A, Miyachi H, Kageyama R (2011). Intronic delay is essential for oscillatory expression in the segmentation clock.. Proc Natl Acad Sci U S A.

[66] Terry AJ, Sturrock M, Dale JK, Maroto M, Chaplain MA (2011). A spatio-temporal model of Notch signalling in the zebrafish segmentation clock: conditions for synchronised oscillatory dynamics.. PLoS ONE.

[67] Hurlbut GD, Kankel MW, Lake RJ, Artavanis-Tsakonas S (2007). Crossing paths with Notch in the hyper-network.. Curr Opin Cell Biol.

[68] Rivett AJ (1998). Intracellular distribution of proteasomes.. Curr Opin Immunol.

[69] Muratani M, Tansey WP (2003). How the ubiquitin-proteasome system controls transcription.. Nat Rev Mol Cell Biol.

[70] Zeiser S, Rivera O, Kuttler C, Hense B, Lasser R (2008). Oscillations of Hes7 caused by negative autoregulation and ubiquitination.. Comput Biol Chem.

[71] Ranganathan P, Vasquez-Del Carpio R, Kaplan FM, Wang H, Gupta A (2011). Hierarchical phosphorylation within the ankyrin repeat domain defines a phosphoregulatory loop that regulates Notch transcriptional activity.. J Biol Chem.

[72] Vasquez-Del Carpio R, Kaplan FM, Weaver KL, VanWye JD, Alves-Guerra MC (2011). Assembly of a Notch transcriptional activation complex requires multimerization.. Mol Cell Biol.

[73] Krol AJ, Roellig D, Dequeant ML, Tassy O, Glynn E (2011). Evolutionary plasticity of segmentation clock networks.. Development.

[74] Eckalbar WL (2011). Major shifts in the evolution of somitogenesis: the reptile Anolis carolinensis represents a fourth type of segmentation clock among vertebrates.. Dev Biol.

[75] Hayashi S, Shimoda T, Nakajima M, Tsukada Y, Sakumura Y (2009). Sprouty4, an FGF inhibitor, displays cyclic gene expression under the control of the notch segmentation clock in the mouse PSM.. PLoS ONE.

[76] Dale JK, Malapert P, Chal J, Vilhais-Neto G, Maroto M (2006). Oscillations of the snail genes in the presomitic mesoderm coordinate segmental patterning and morphogenesis in vertebrate somitogenesis.. Dev Cell.

[77] Pirot P, van Grunsven LA, Marine JC, Huylebroeck D, Bellefroid EJ (2004). Direct regulation of the Nrarp gene promoter by the Notch signaling pathway.. Biochem Biophys Res Commun.

[78] Ishikawa A, Kitajima S, Takahashi Y, Kokubo H, Kanno J (2004). Mouse Nkd1, a Wnt antagonist, exhibits oscillatory gene expression in the PSM under the control of Notch signaling.. Mech Dev.

[79] Gibb S, Maroto M, Dale JK (2010). The segmentation clock mechanism moves up a notch.. Trends Cell Biol.

[80] Yu HM, Jerchow B, Sheu TJ, Liu B, Costantini F (2005). The role of Axin2 in calvarial morphogenesis and craniosynostosis.. Development.

[81] Serth K, Schuster-Gossler K, Cordes R, Gossler A (2003). Transcriptional oscillation of lunatic fringe is essential for somitogenesis.. Genes Dev.

[82] Dale JK, Maroto M, Dequeant ML, Malapert P, McGrew M (2003). Periodic notch inhibition by lunatic fringe underlies the chick segmentation clock.. Nature.

[83] Shifley ET, Cole SE (2008). Lunatic fringe protein processing by proprotein convertases may contribute to the short protein half-life in the segmentation clock.. Biochim Biophys Acta.

[84] Jiang YJ, Aerne BL, Smithers L, Haddon C, Ish-Horowicz D (2000). Notch signalling and the synchronization of the somite segmentation clock.. Nature.

[85] Elmasri H, Liedtke D, Lucking G, Volff JN, Gessler M (2004). her7 and hey1, but not lunatic fringe show dynamic expression during somitogenesis in medaka (Oryzias latipes).. Gene Expr Patterns.

[86] Horikawa K, Ishimatsu K, Yoshimoto E, Kondo S, Takeda H (2006). Noise-resistant and synchronized oscillation of the segmentation clock.. Nature.

[87] Kobayashi T, Mizuno H, Imayoshi I, Furusawa C, Shirahige K (2009). The cyclic gene Hes1 contributes to diverse differentiation responses of embryonic stem cells.. Genes Dev.

[88] Hou X, Tashima Y, Stanley P (2012). Galactose differentially modulates lunatic and manic fringe effects on Delta1-induced NOTCH signaling.. J Biol Chem.

[89] Oberg C, Li J, Pauley A, Wolf E, Gurney M (2001). The Notch intracellular domain is ubiquitinated and negatively regulated by the mammalian Sel-10 homolog.. J Biol Chem.

[90] Zhou BP, Deng J, Xia W, Xu J, Li YM (2004). Dual regulation of Snail by GSK-3beta-mediated phosphorylation in control of epithelial-mesenchymal transition.. Nat Cell Biol.

[91] Farin HF, Bussen M, Schmidt MK, Singh MK, Schuster-Gossler K (2007). Transcriptional repression by the T-box proteins Tbx18 and Tbx15 depends on Groucho corepressors.. J Biol Chem.

[92] Farin HF, Mansouri A, Petry M, Kispert A (2008). T-box protein Tbx18 interacts with the paired box protein Pax3 in the development of the paraxial mesoderm.. J Biol Chem.

[93] Bussen M, Petry M, Schuster-Gossler K, Leitges M, Gossler A (2004). The T-box transcription factor Tbx18 maintains the separation of anterior and posterior somite compartments.. Genes Dev.

[94] Rallis C, Pinchin SM, Ish-Horowicz D (2010). Cell-autonomous integrin control of Wnt and Notch signalling during somitogenesis.. Development.

[95] Koizumi K, Nakajima M, Yuasa S, Saga Y, Sakai T (2001). The role of presenilin 1 during somite segmentation.. Development.

[96] Kusumi K, Mimoto MS, Covello KL, Beddington RS, Krumlauf R (2004). Dll3 pudgy mutation differentially disrupts dynamic expression of somite genes.. Genesis.

[97] Sewell W, Sparrow DB, Smith AJ, Gonzalez DM, Rappaport EF (2009). Cyclical expression of the Notch/Wnt regulator Nrarp requires modulation by Dll3 in somitogenesis.. Dev Biol.

[98] Vermot J, Gallego Llamas J, Fraulob V, Niederreither K, Chambon P (2005). Retinoic acid controls the bilateral symmetry of somite formation in the mouse embryo.. Science.

[99] Goldbeter A, Gonze D, Pourquie O (2007). Sharp developmental thresholds defined through bistability by antagonistic gradients of retinoic acid and FGF signaling.. Dev Dyn.

[100] Cunningham TJ, Zhao X, Duester G (2011). Uncoupling of retinoic acid signaling from tailbud development before termination of body axis extension.. Genesis.

[101] Ekerot M, Stavridis MP, Delavaine L, Mitchell MP, Staples C (2008). Negative-feedback regulation of FGF signalling by DUSP6/MKP-3 is driven by ERK1/2 and mediated by Ets factor binding to a conserved site within the DUSP6/MKP-3 gene promoter.. Biochem J.

[102] Dequeant ML, Pourquie O (2008). Segmental patterning of the vertebrate embryonic axis.. Nat Rev Genet.

[103] Jorgensen C, Sherman A, Chen GI, Pasculescu A, Poliakov A (2009). Cell-specific information processing in segregating populations of Eph receptor ephrin-expressing cells.. Science.

[104] Uriu K, Morishita Y, Iwasa Y (2010). Random cell movement promotes synchronization of the segmentation clock.. Proc Natl Acad Sci U S A.

[105] Benazeraf B, Francois P, Baker RE, Denans N, Little CD (2010). A random cell motility gradient downstream of FGF controls elongation of an amniote embryo.. Nature.

[106] Cohen M, Georgiou M, Stevenson NL, Miodownik M, Baum B (2010). Dynamic filopodia transmit intermittent Delta-Notch signaling to drive pattern refinement during lateral inhibition.. Dev Cell.

[107] Riedel-Kruse IH, Muller C, Oates AC (2007). Synchrony dynamics during initiation, failure, and rescue of the segmentation clock.. Science.

[108] Sheldon H, Heikamp E, Turley H, Dragovic R, Thomas P (2010). New mechanism for Notch signaling to endothelium at a distance by Delta-like 4 incorporation into exosomes.. Blood.

[109] Thiery JP, Acloque H, Huang RY, Nieto MA (2009). Epithelial-mesenchymal transitions in development and disease.. Cell.

[110] Rifes P, Carvalho L, Lopes C, Andrade RP, Rodrigues G (2007). Redefining the role of ectoderm in somitogenesis: a player in the formation of the fibronectin matrix of presomitic mesoderm.. Development.

[111] Julich D, Mould AP, Koper E, Holley SA (2009). Control of extracellular matrix assembly along tissue boundaries via Integrin and Eph/Ephrin signaling.. Development.

[112] Jouve C, Palmeirim I, Henrique D, Beckers J, Gossler A (2000). Notch signalling is required for cyclic expression of the hairy-like gene HES1 in the presomitic mesoderm.. Development.

[113] Sasaki N, Kiso M, Kitagawa M, Saga Y (2011). The repression of Notch signaling occurs via the destabilization of mastermind-like 1 by Mesp2 and is essential for somitogenesis.. Development.

