# Correction: From Dynamic Expression Patterns to Boundary Formation in the Presomitic Mesoderm

**DOI:** 10.1371/journal.pcbi.1007191

**Published:** 2019-07-02

**Authors:** Hendrik B. Tiedemann, Elida Schneltzer, Stefan Zeiser, Bastian Hoesel, Johannes Beckers, Gerhard K. H. Przemeck, Martin Hrabě de Angelis

S10 Video File is incorrect on the published article. It can be viewed below.

## Supporting information

S10 Video File Time evolution of cytoplasmic *Hes7* mRNA concentration for the model shown in Figure S2. (AVI)
