# Supplementary material for: From Dynamic Expression Patterns to Boundary Formation in the Presomitic Mesoderm
Source: PLoS Comput Biol. 2012 Jun 28;8(6):e1002586. doi: 10.1371/journal.pcbi.1002586 (PMC3386180; doi:10.1371/journal.pcbi.1002586)
Supplement: Dataset S1 — Parameter discussion for the core oscillator (HES7 and D/N). (A) Influence of parameter variations on the HES7 oscillation amplitude (first column) (maximum in blue, minimum in red), HES7 oscillation period (second column), cytoplasmic NICD oscillation amplitude (third column), and NICD oscillation period (fourth column). (B) Parameter ranges in percentage (orange rectangles) out of which individual parameters were drawn from a uniform distribution. Vertical red line at 100% denotes default parameter values. Boundaries of the rectangles correspond to the minimal and maximal parameter variations. (C) Histogram: Distribution of period lengths of PN_Hes7 for 100 randomly drawn parameter configurations. (PDF) [file pcbi.1002586.s001.pdf]

(A)

Maximum rate of NICD degradation = 0.02				
	**Minimum and maximum concentration of HES7 pC (*y axis*)**	**Oscillation period of HES7 pC (*y axis*)**	**Minimum and maximum concentration of NICD pC (*y axis*)**	**Oscillation period of NICD pC (*y axis*)**
***Dll1* mC** decay **(*x axis*)**	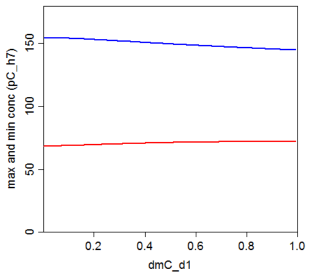	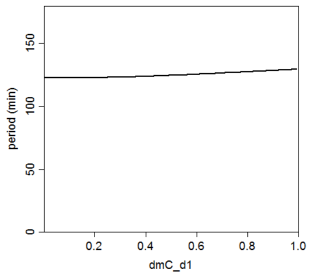	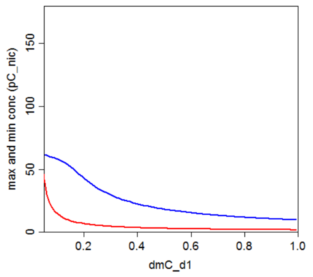	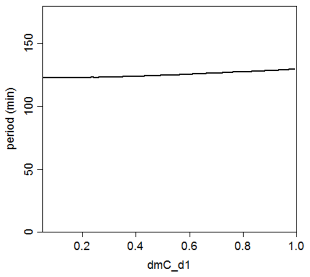
***Dll1* mN** decay **(*x axis*)**	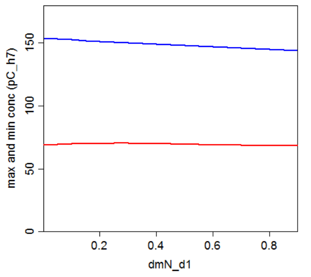	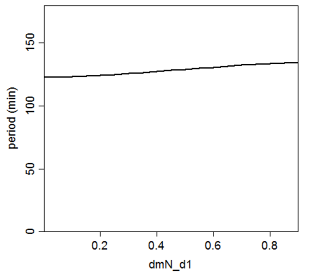	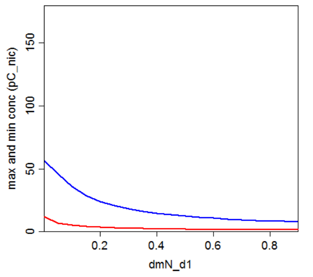	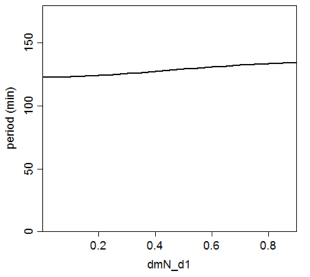
**DLL1 pC** decay **(*x axis*)**	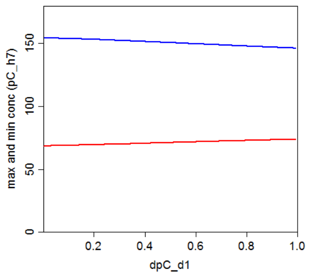	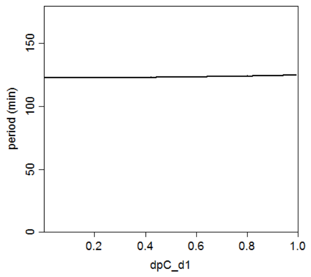	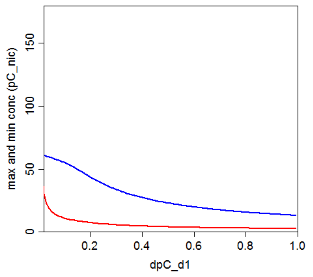	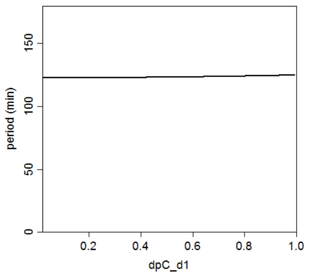

m: mRNA
mC: mRNA in cytoplasm
mN: mRNA in nucleus

p: protein
pC: protein in cytoplasm
pN: protein in nucleus

___: minimal oscillation amplitude
___: maximal oscillation amplitude
___: time period in minutes

<table>
<tr><th colspan="5">Maximum rate of NICD degradation = 0.02</th></tr>
<tr><th></th><th>Minimum and maximum concentration of HES7 pC (y axis)</th><th>Oscillation period of HES7 pC (y axis)</th><th>Minimum and maximum concentration of NICD pC (y axis)</th><th>Oscillation period of NICD pC (y axis)</th></tr>
<tr><td>Dll1 mN export (x axis)</td>
<td>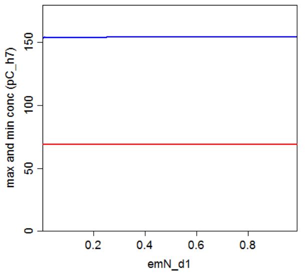</td>
<td>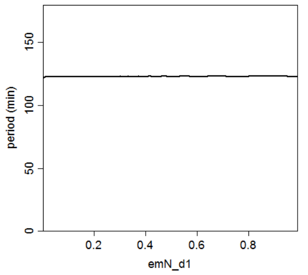</td>
<td>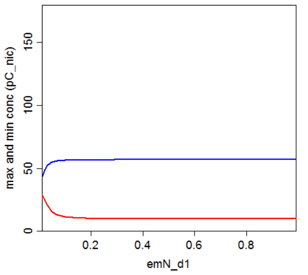</td>
<td>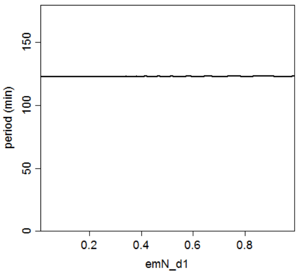</td></tr>
<tr><td>DLL1 pC export (x axis)</td>
<td>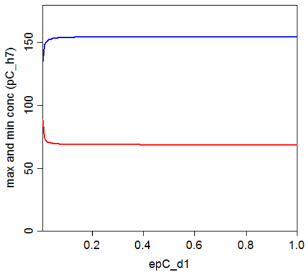</td>
<td>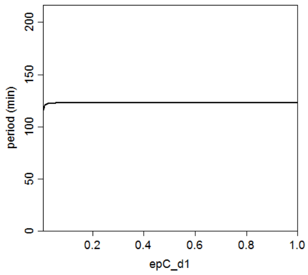</td>
<td>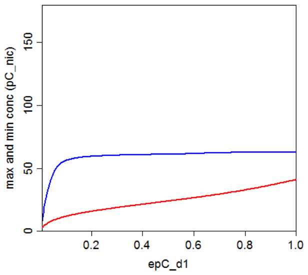</td>
<td>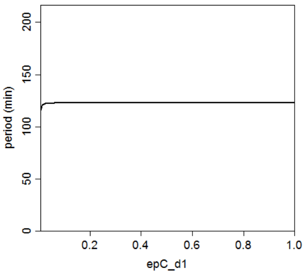</td></tr>
<tr><td>DLL1 pM export (x axis)</td>
<td>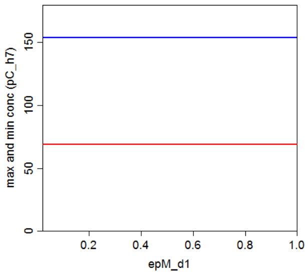</td>
<td>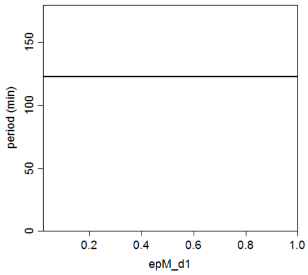</td>
<td>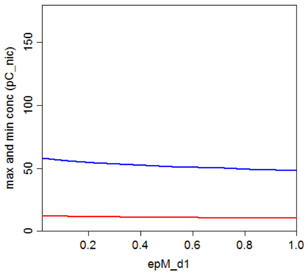</td>
<td>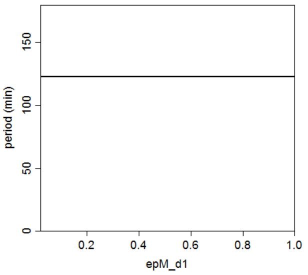</td></tr>
<tr><td>DLL1/ NOTCH1 reaction factor (x axis)</td>
<td>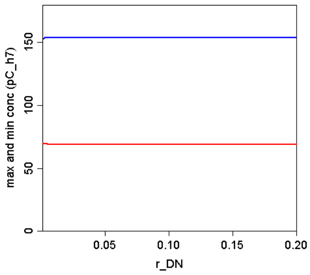</td>
<td>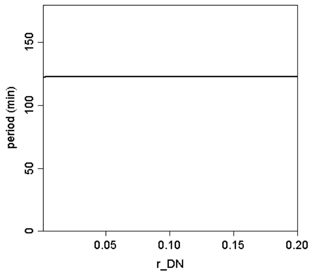</td>
<td>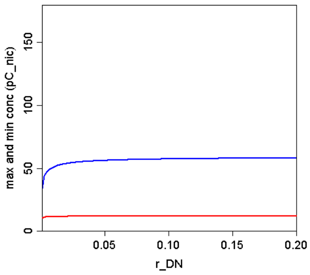</td>
<td>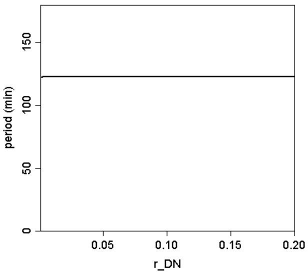</td></tr>
</table>

m: mRNA
mC: mRNA in cytoplasm
mN: mRNA in nucleus

p: protein
pC: protein in cytoplasm
pN: protein in nucleus

___ (red): minimal oscillation amplitude
___ (blue): maximal oscillation amplitude
___ (black): time period in minutes

Maximum rate of NICD degradation = 0.02				
	**Minimum and maximum concentration of HES7 pC (*y axis*)**	**Oscillation period of HES7 pC (*y axis*)**	**Minimum and maximum concentration of NICD pC (*y axis*)**	**Oscillation period of NICD pC (*y axis*)**
***Notch1* m** decay **(*x axis*)**	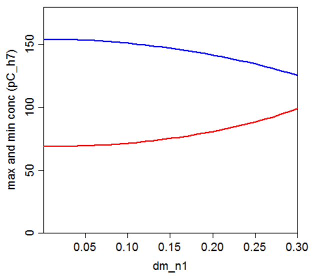	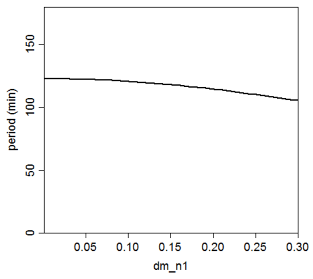	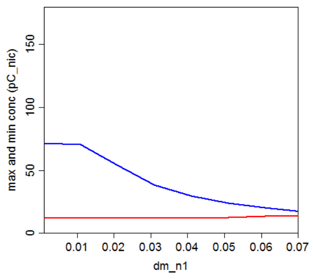	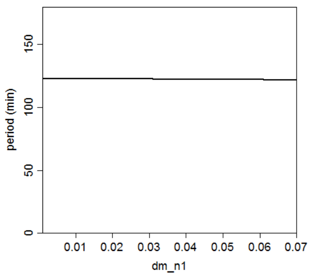
**NOTCH1 pC** decay **(*x axis*)**	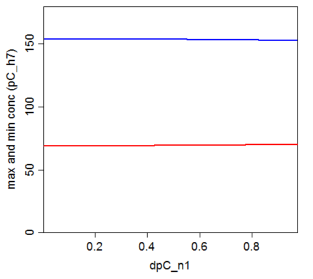	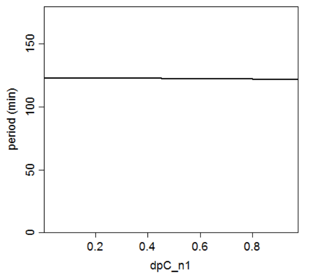	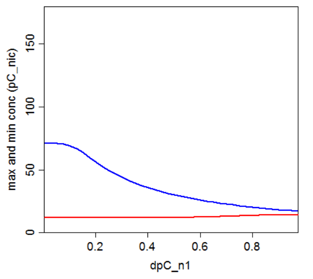	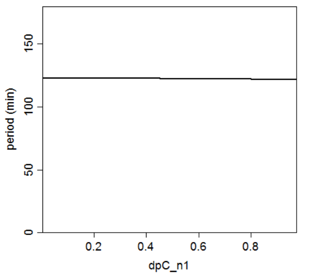
**NOTCH1 pM** decay **(*x axis*)**	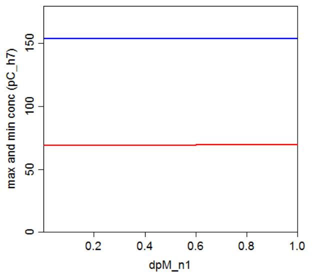	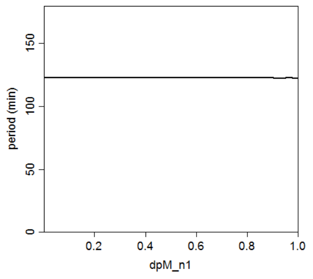	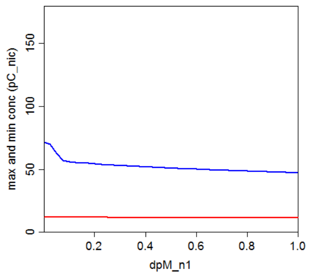	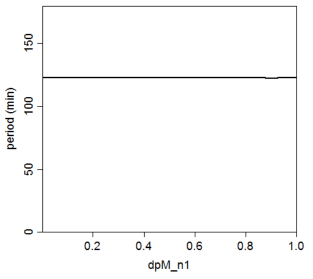
**NOTCH1 pC** export **(*x axis*)**	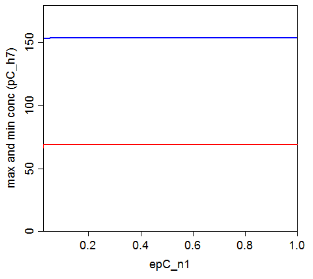	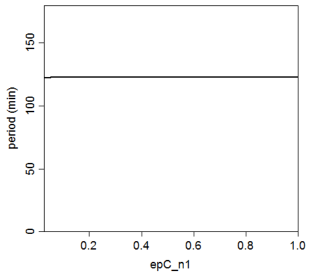	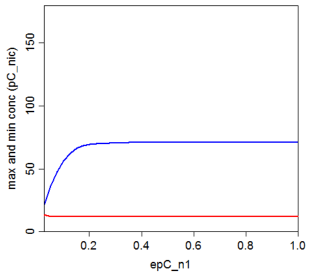	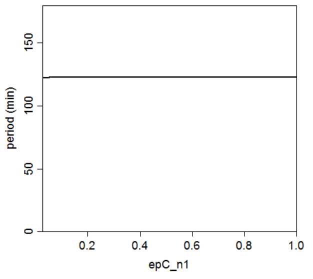

m: mRNA
mC: mRNA in cytoplasm
mN: mRNA in nucleus

p: protein
pC: protein in cytoplasm
pN: protein in nucleus

___ (red): minimal oscillation amplitude
___ (blue): maximal oscillation amplitude
___ (black): time period in minutes

Maximum rate of NICD degradation = 0.02				
	**Minimum and maximum concentration of HES7 pC (*y axis*)**	**Oscillation period of HES7 pC (*y axis*)**	**Minimum and maximum concentration of NICD pC (*y axis*)**	**Oscillation period of NICD pC (*y axis*)**
**NOTCH1** maximal trans-lation rate **(*x axis*)**	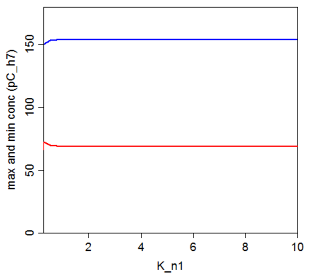	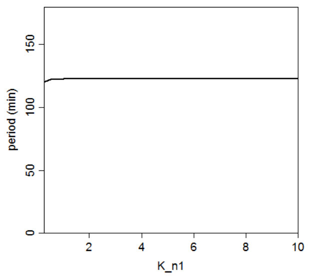	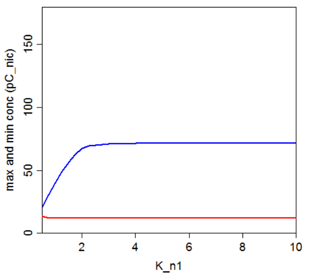	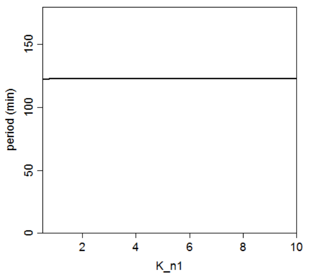
Notch1 trans-cription rate **(*x axis*)**	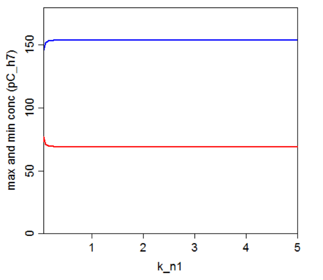	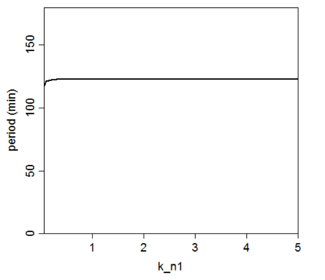	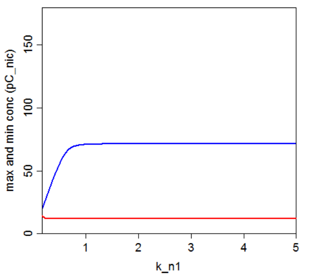	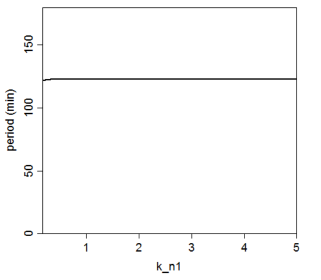
Hes7 **mC** decay **(*x axis*)**	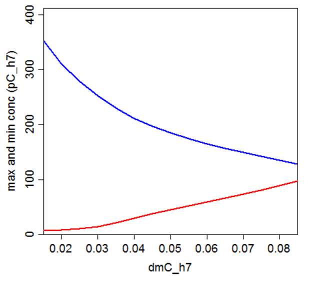	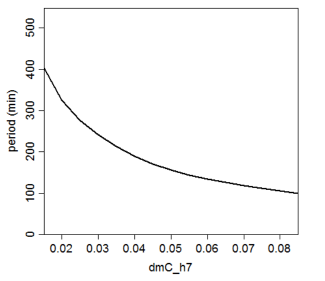	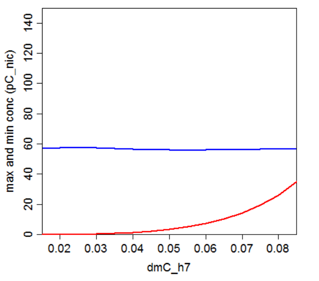	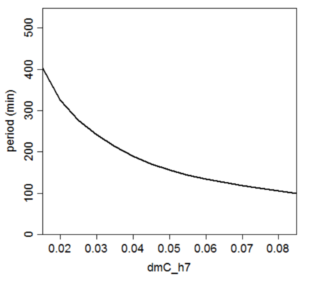
Hes7 **mN** decay **(*x axis*)**	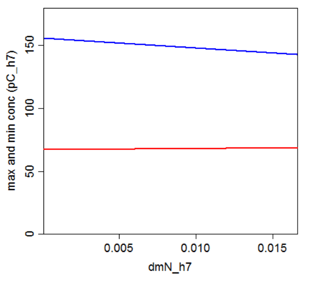	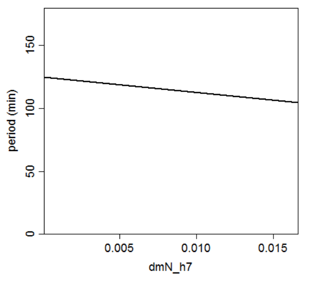	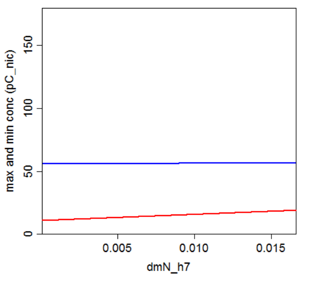	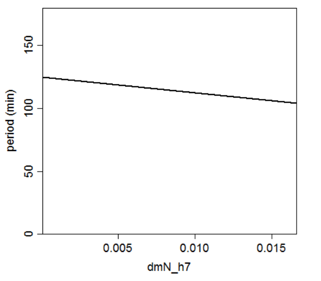

m: mRNA
mC: mRNA in cytoplasm
mN: mRNA in nucleus

p: protein
pC: protein in cytoplasm
pN: protein in nucleus

___: minimal oscillation amplitude
___: maximal oscillation amplitude
___: time period in minutes

Maximum rate of NICD degradation = 0.02				
	**Minimum and maximum concentration of HES7 pC (*y axis*)**	**Oscillation period of HES7 pC (*y axis*)**	**Minimum and maximum concentration of NICD pC (*y axis*)**	**Oscillation period of NICD pC (*y axis*)**
**HES7 pC** decay **(*x axis*)**	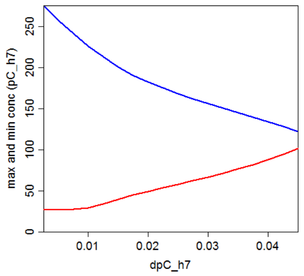	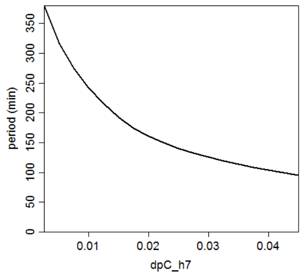	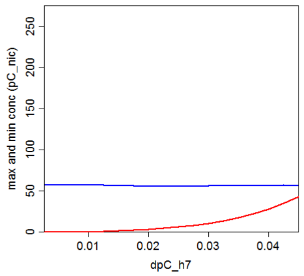	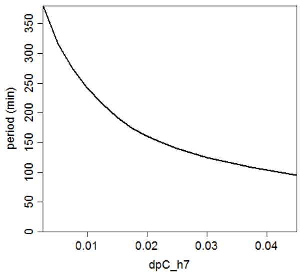
***Hes7* mN** export **(*x axis*)**	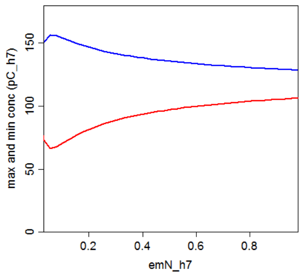	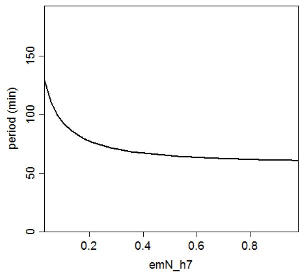	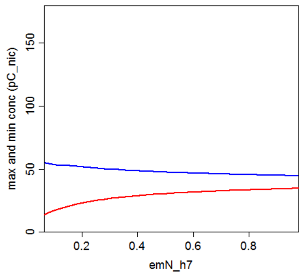	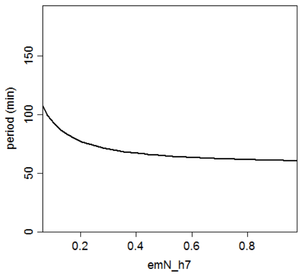
**HES7 pC** export **(*x axis*)**	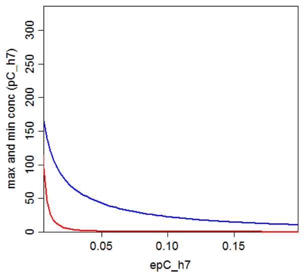	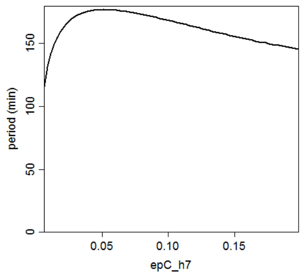	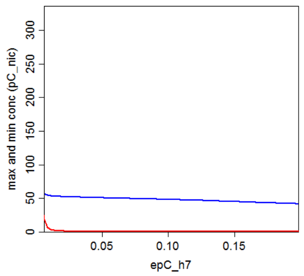	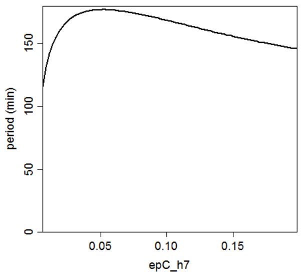
**HES7 pN** export **(*x axis*)**	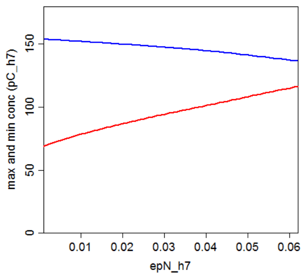	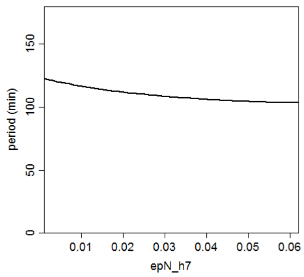	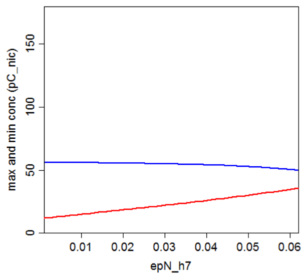	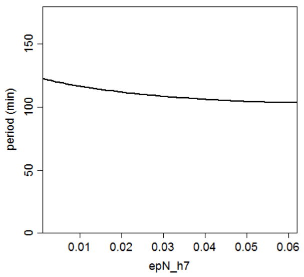

m: mRNA — **p**: protein — ___: minimal oscillation amplitude (red)

mC: mRNA in cytoplasm — **pC**: protein in cytoplasm — ___: maximal oscillation amplitude (blue)

mN: mRNA in nucleus — **pN**: protein in nucleus — ___: time period in minutes (black)

Maximum rate of NICD degradation = 0.02				
	**Minimum and maximum concentration of HES7 pC (*y axis*)**	**Oscillation period of HES7 pC (*y axis*)**	**Minimum and maximum concentration of NICD pC (*y axis*)**	**Oscillation period of NICD pC (*y axis*)**
Michaelis constant of **HES7** degra-dation **(*x axis*)**	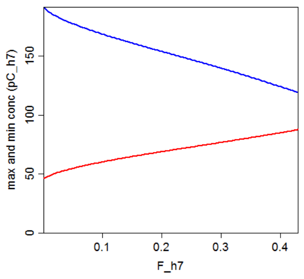	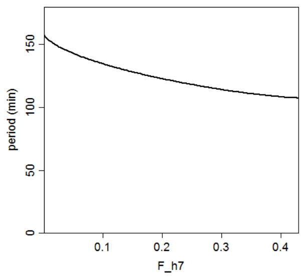	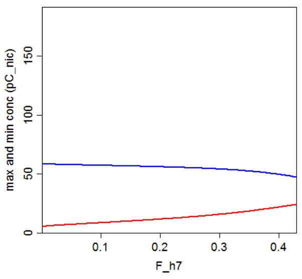	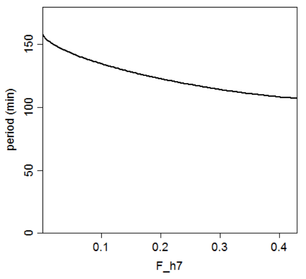
Maximal rate of **HES7** degra-dation **(*x axis*)**	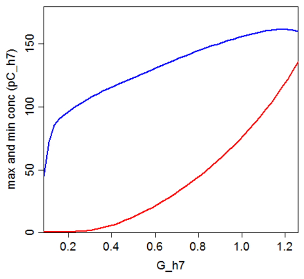	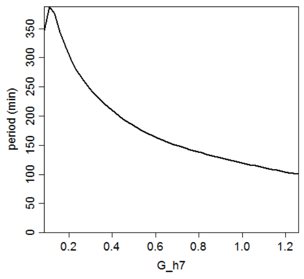	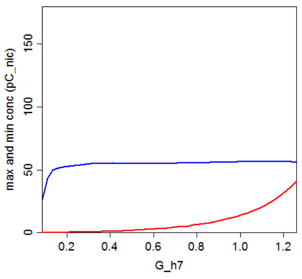	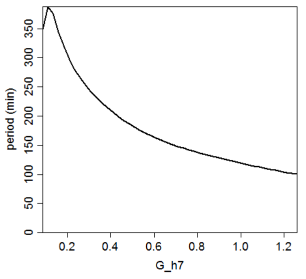
**HES7** trans-lation rate **(*x axis*)**	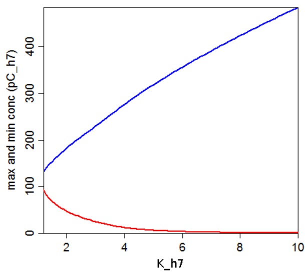	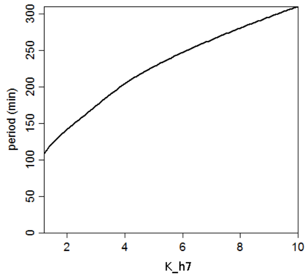	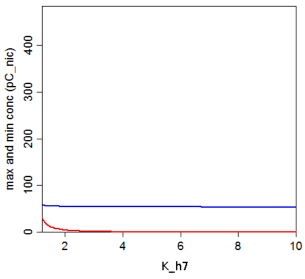	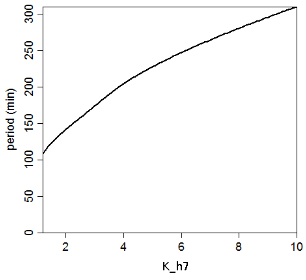
Hes7 trans-cription rate **(*x axis*)**	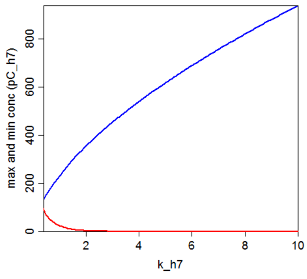	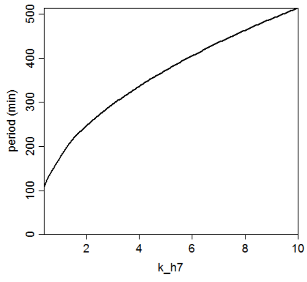	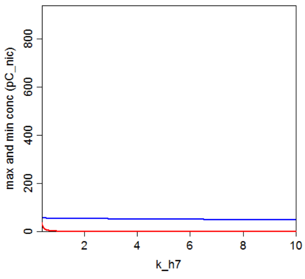	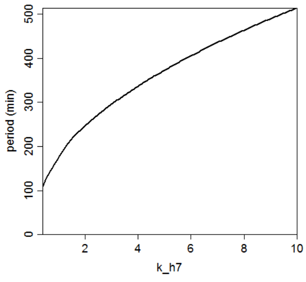

m: mRNA
mC: mRNA in cytoplasm
mN: mRNA in nucleus

p: protein
pC: protein in cytoplasm
pN: protein in nucleus

___: minimal oscillation amplitude
___: maximal oscillation amplitude
___: time period in minutes

Maximum rate of NICD degradation = 0.02				
	**Minimum and maximum concentration of HES7 pC (*y axis*)**	**Oscillation period of HES7 pC (*y axis*)**	**Minimum and maximum concentration of NICD pC (*y axis*)**	**Oscillation period of NICD pC (*y axis*)**
**NICD pC** decay **(*x axis*)**	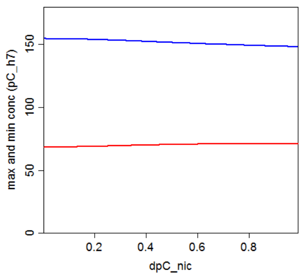	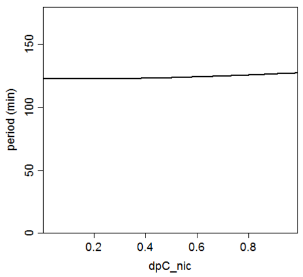	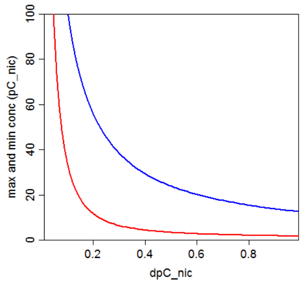	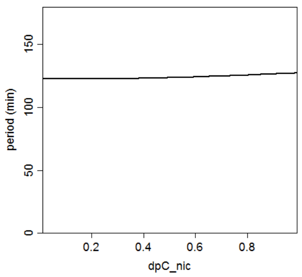
**NICD pC** export **(*x axis*)**	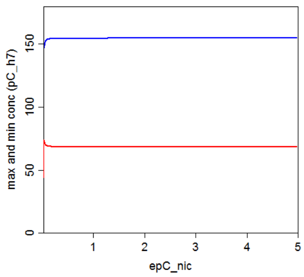	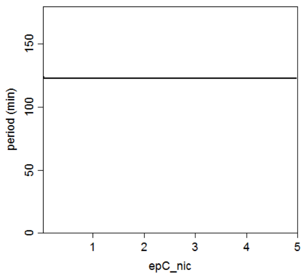	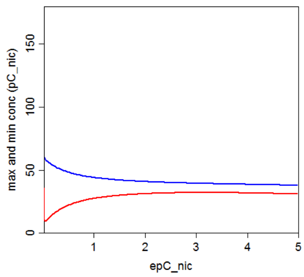	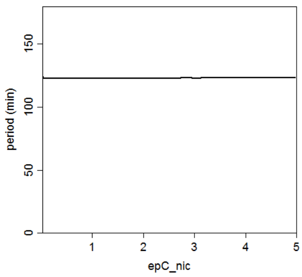
**NICD pN** export **(*x axis*)**	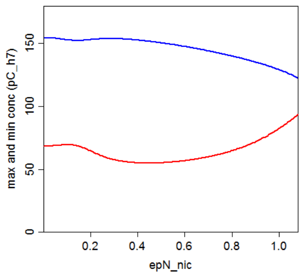	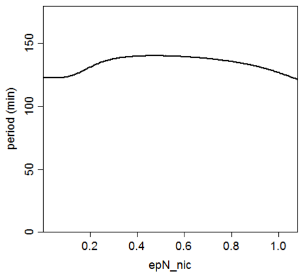	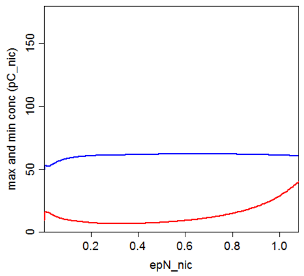	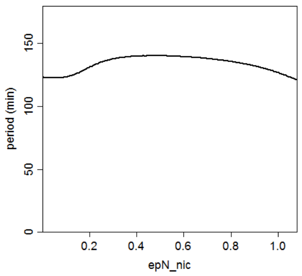
Michaelis constant of **NICD** degra-dation **(*x axis*)**	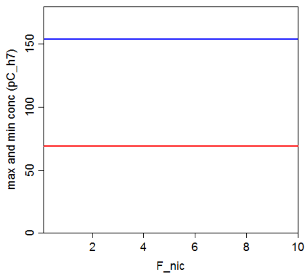	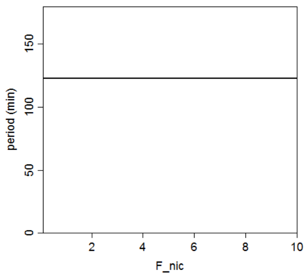	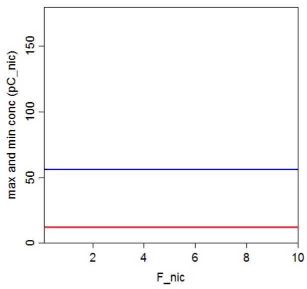	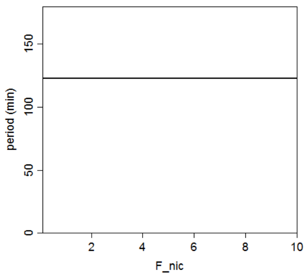

m: mRNA
mC: mRNA in cytoplasm
mN: mRNA in nucleus

p: protein
pC: protein in cytoplasm
pN: protein in nucleus

___: minimal oscillation amplitude
___: maximal oscillation amplitude
___: time period in minutes

Maximum rate of NICD degradation = 0.02

	Minimum and maximum concentration of HES7 pC (*y axis*)	Oscillation period of HES7 pC (*y axis*)	Minimum and maximum concentration of NICD pC (*y axis*)	Oscillation period of NICD pC (*y axis*)
**NICD** *Hill threshold* **(*x axis*)**	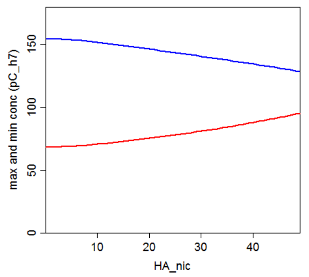	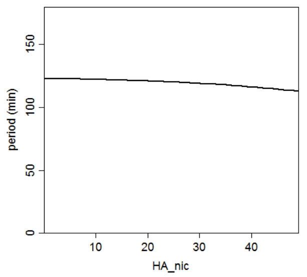	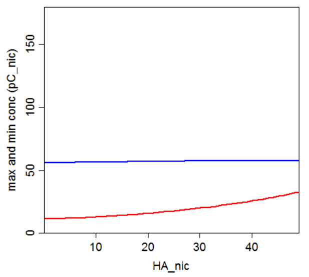	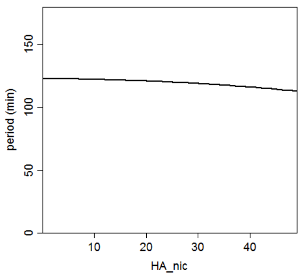
**Hes7** *Hill threshold* **(*x axis*)**	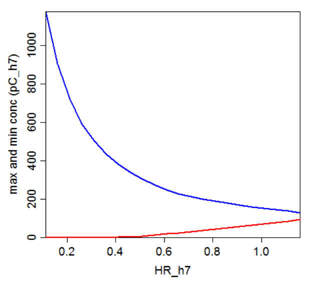	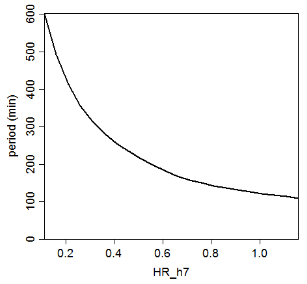	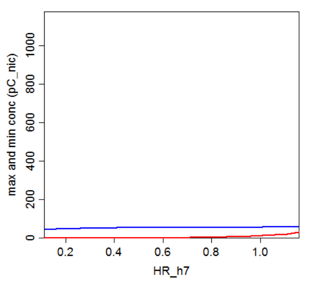	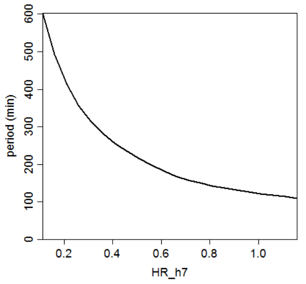

Maximum rate of NICD degradation = 5.0

	Minimum and maximum concentration of HES7 pC (*y axis*)	Oscillation period of HES7 pC (y axis)	Minimum and maximum concentration of NICD pC (*y axis*)	Oscillation period of NICD pC (y axis)
***Dll1* mC** decay **(*x axis*)**	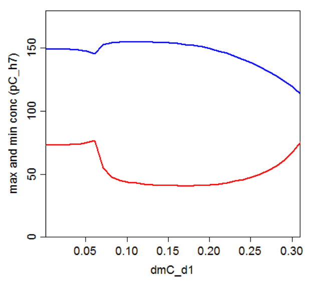	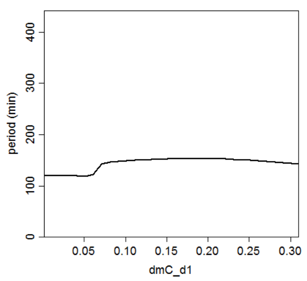	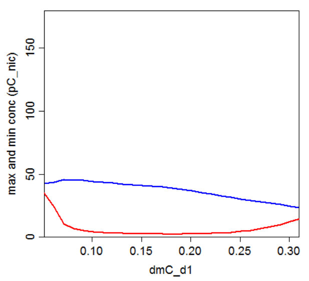	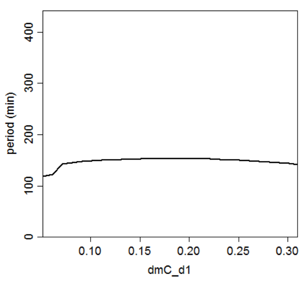

m: mRNA
mC: mRNA in cytoplasm
mN: mRNA in nucleus

p: protein
pC: protein in cytoplasm
pN: protein in nucleus

___: minimal oscillation amplitude
___: maximal oscillation amplitude
___: time period in minutes

Maximum rate of NICD degradation = 5.0				
	**Minimum and maximum concentration of HES7 pC (*y axis*)**	**Oscillation period of HES7 pC (y axis)**	**Minimum and maximum concentration of NICD pC (*y axis*)**	**Oscillation period of NICD pC (y axis)**
***Dll1* mN** decay **(*x axis*)**	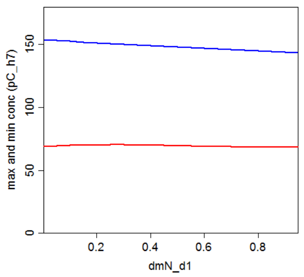	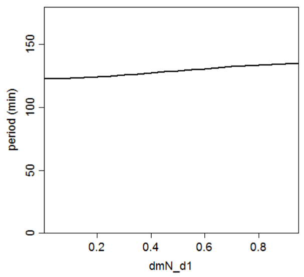	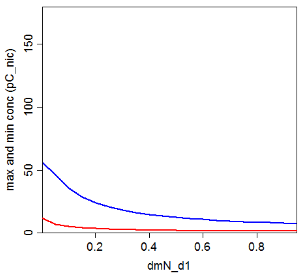	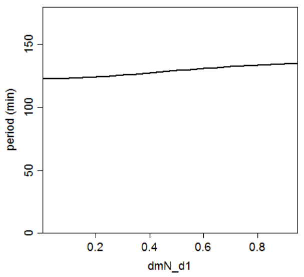
**DLL1 pC** decay **(*x axis*)**	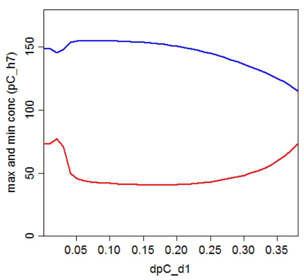	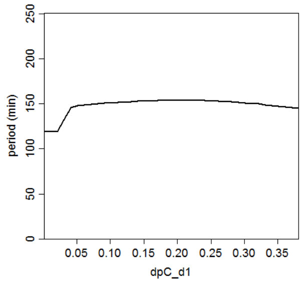	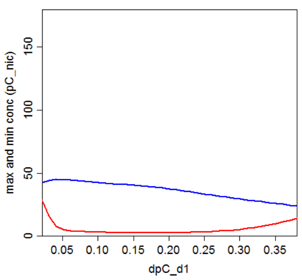	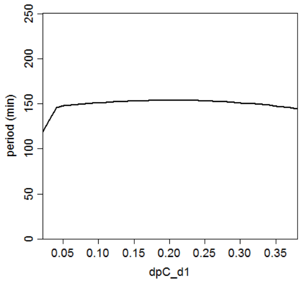
***Dll1* mN** export **(*x axis*)**	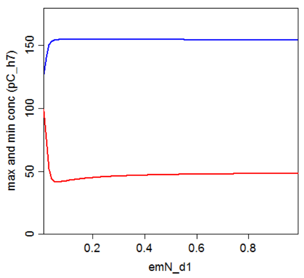	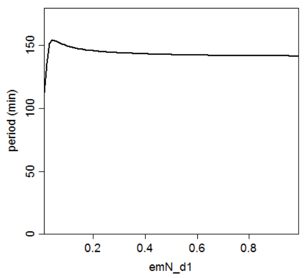	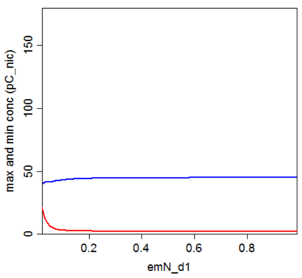	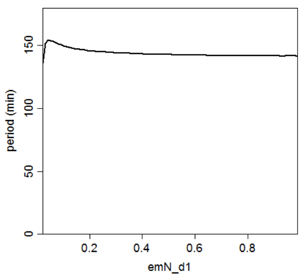
**DLL1 pC** export **(*x axis*)**	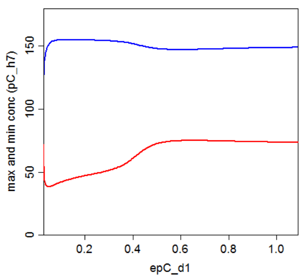	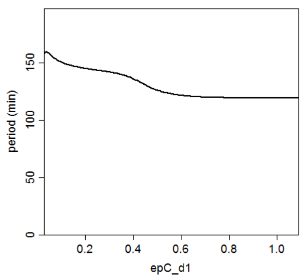	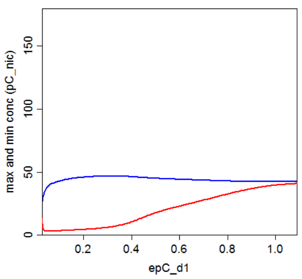	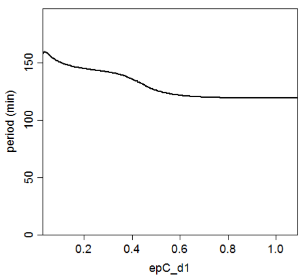

m: mRNA — **p**: protein — ___ (red): minimal oscillation amplitude

mC: mRNA in cytoplasm — **pC**: protein in cytoplasm — ___ (blue): maximal oscillation amplitude

mN: mRNA in nucleus — **pN**: protein in nucleus — ___ (black): time period in minutes

<table>
<tr><th colspan="5">Maximum rate of NICD degradation = 5.0</th></tr>
<tr><th></th><th>Minimum and maximum concentration of HES7 pC (y axis)</th><th>Oscillation period of HES7 pC (y axis)</th><th>Minimum and maximum concentration of NICD pC (y axis)</th><th>Oscillation period of NICD pC (y axis)</th></tr>
<tr><td>DLL1 pM export (x axis)</td>
<td>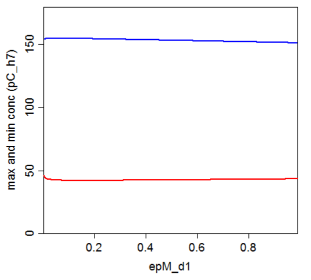</td>
<td>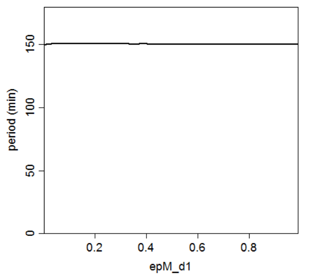</td>
<td>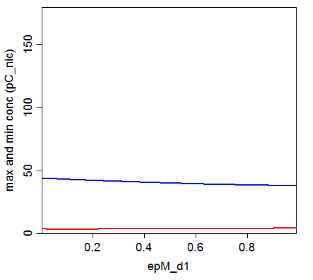</td>
<td>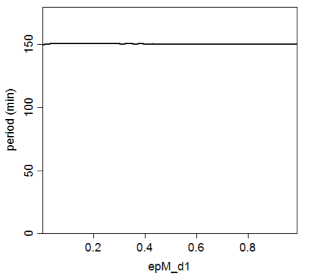</td></tr>
<tr><td>DLL1/ NOTCH1 reaction factor (x axis)</td>
<td>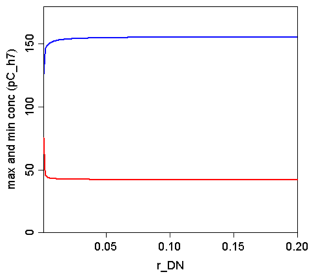</td>
<td>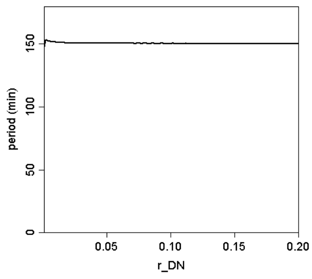</td>
<td>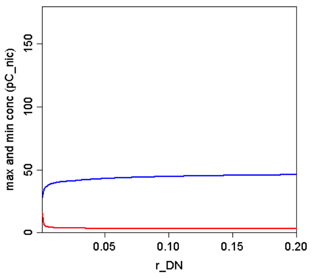</td>
<td>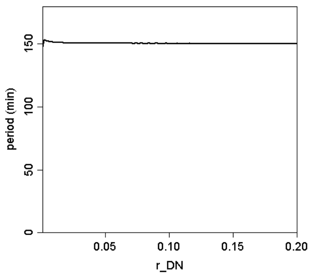</td></tr>
<tr><td>Notch1 m decay (x axis)</td>
<td>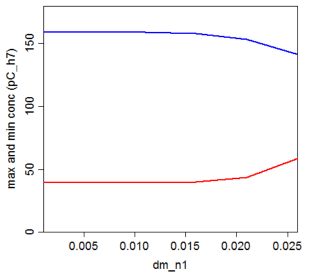</td>
<td>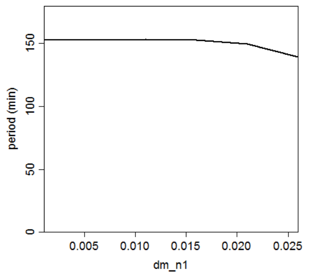</td>
<td>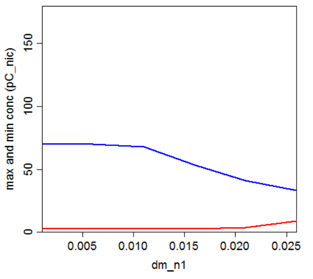</td>
<td>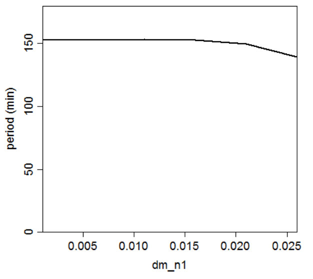</td></tr>
<tr><td>NOTCH1 pC decay (x axis)</td>
<td>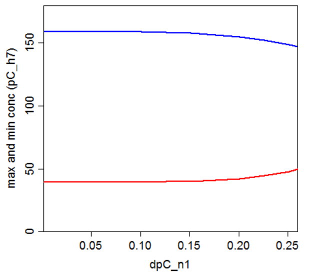</td>
<td>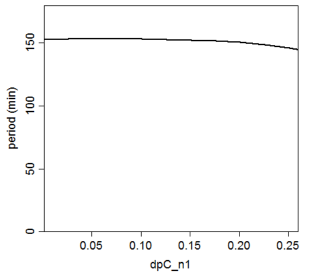</td>
<td>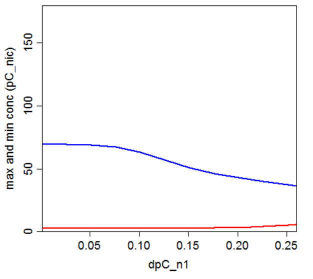</td>
<td>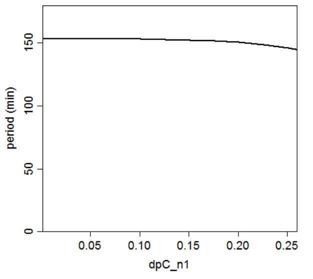</td></tr>
</table>

m: mRNA
mC: mRNA in cytoplasm
mN: mRNA in nucleus

p: protein
pC: protein in cytoplasm
pN: protein in nucleus

___: minimal oscillation amplitude
___: maximal oscillation amplitude
___: time period in minutes

Maximum rate of NICD degradation = 5.0				
	**Minimum and maximum concentration of HES7 pC (*y axis*)**	**Oscillation period of HES7 pC (y axis)**	**Minimum and maximum concentration of NICD pC (*y axis*)**	**Oscillation period of NICD pC (y axis)**
**NOTCH1 pM** decay **(*x axis*)**	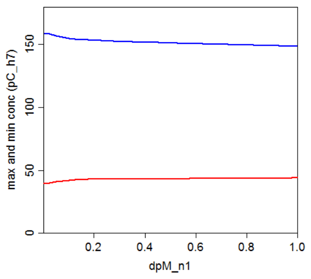	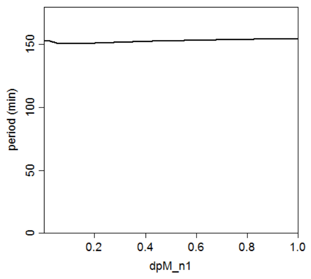	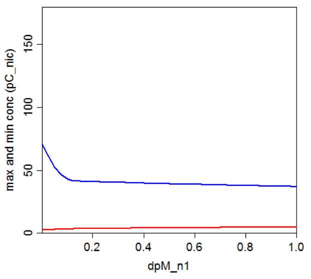	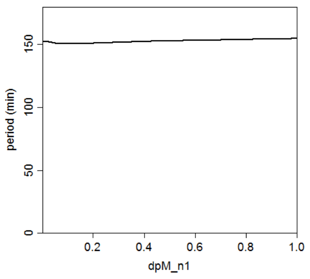
**NOTCH1 pC** export **(*x axis*)**	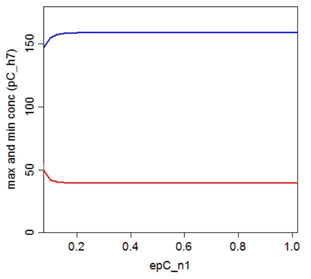	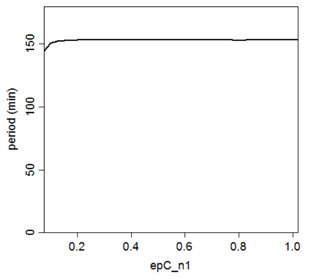	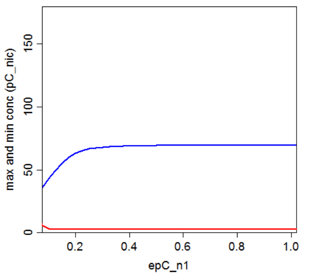	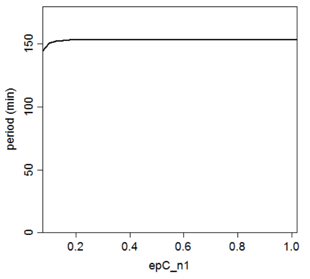
**NOTCH1** maximal trans-lation rate **(*x axis*)**	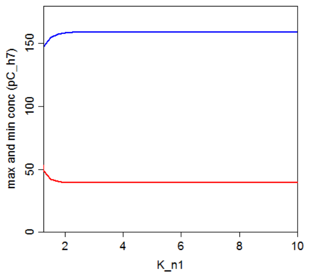	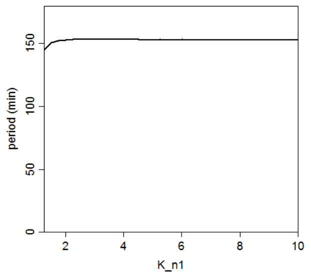	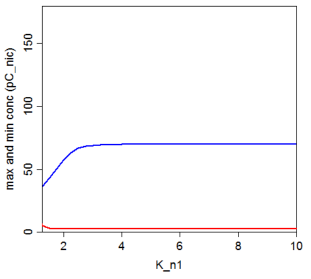	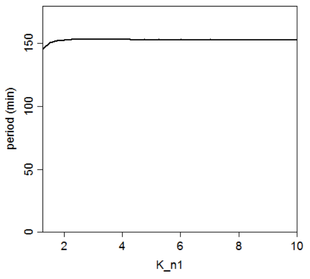
Notch1 trans-cription rate **(*x axis*)**	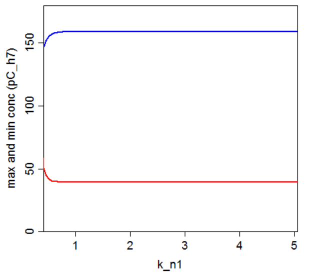	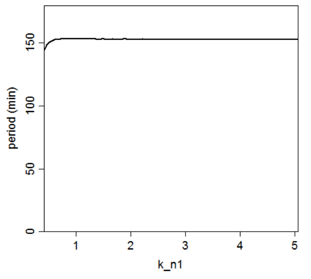	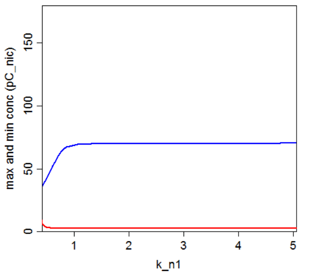	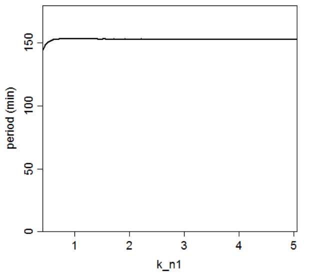

m: mRNA
mC: mRNA in cytoplasm
mN: mRNA in nucleus

p: protein
pC: protein in cytoplasm
pN: protein in nucleus

___: minimal oscillation amplitude
___: maximal oscillation amplitude
___: time period in minutes

Maximum rate of NICD degradation = 5.0				
	**Minimum and maximum concentration of HES7 pC (*y axis*)**	**Oscillation period of HES7 pC (y axis)**	**Minimum and maximum concentration of NICD pC (*y axis*)**	**Oscillation period of NICD pC (y axis)**
***Hes7* mC** decay **(*x axis*)**	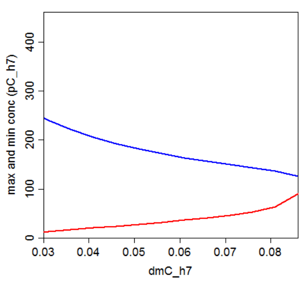	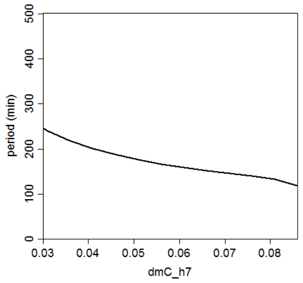	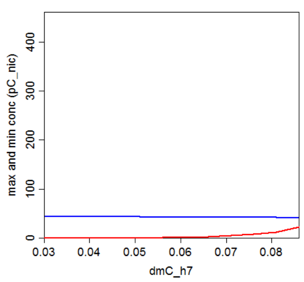	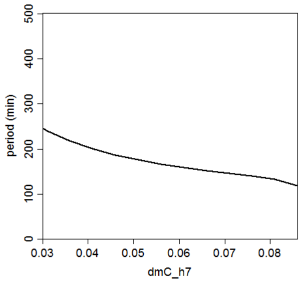
***Hes7* mN** decay **(*x axis*)**	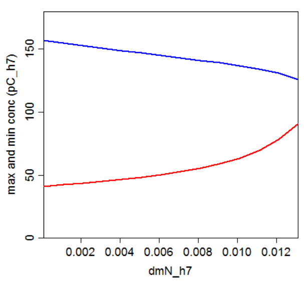	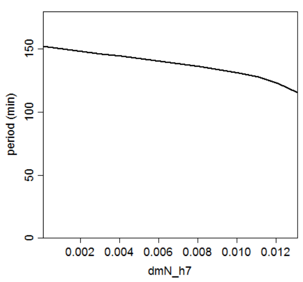	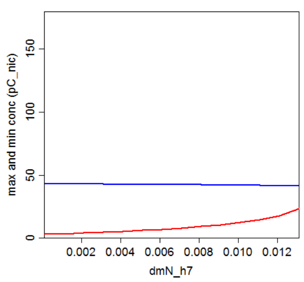	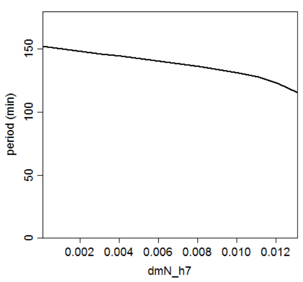
**HES7 pC** decay **(*x axis*)**	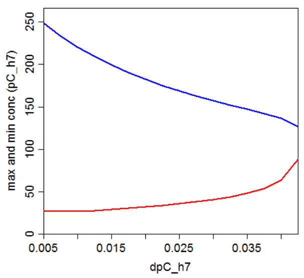	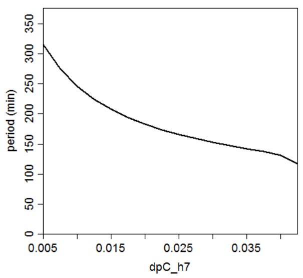	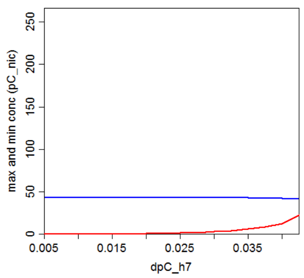	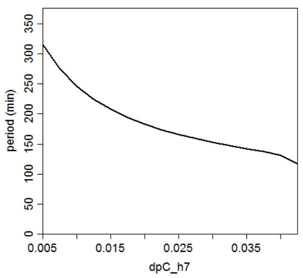
***Hes7* mN** export **(*x axis*)**	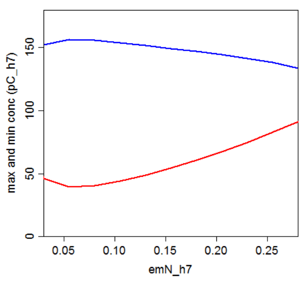	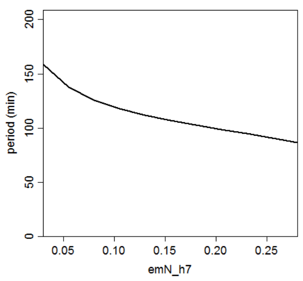	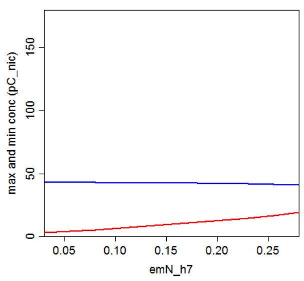	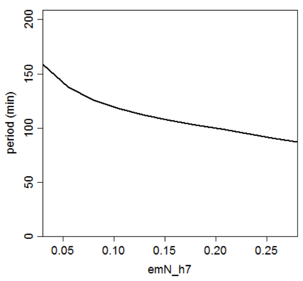

m: mRNA
mC: mRNA in cytoplasm
mN: mRNA in nucleus

p: protein
pC: protein in cytoplasm
pN: protein in nucleus

___: minimal oscillation amplitude
___: maximal oscillation amplitude
___: time period in minutes

Maximum rate of NICD degradation = 5.0				
	**Minimum and maximum concentration of HES7 pC (*y axis*)**	**Oscillation period of HES7 pC (y axis)**	**Minimum and maximum concentration of NICD pC (*y axis*)**	**Oscillation period of NICD pC (y axis)**
**HES7 pC** export **(*x axis*)**	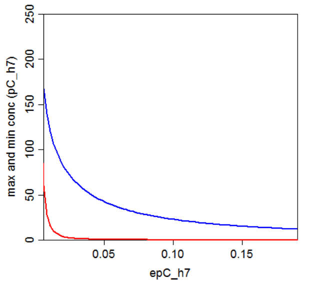	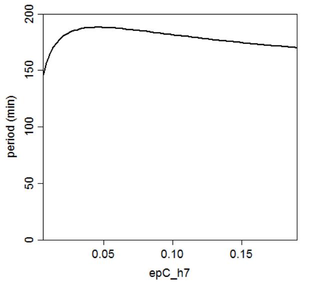	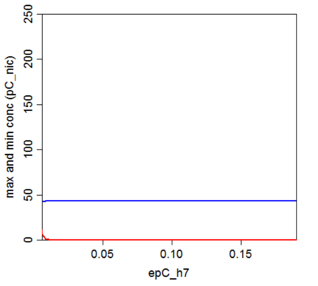	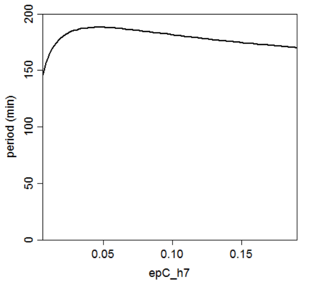
**HES7 pN** export **(*x axis*)**	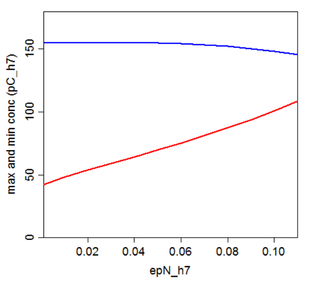	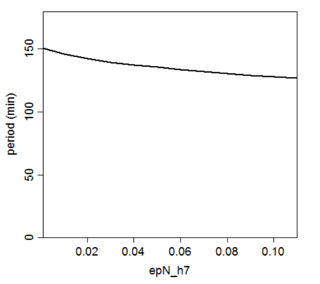	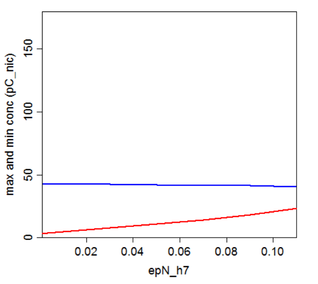	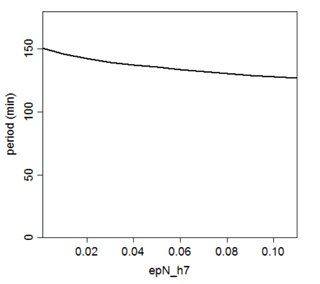
Michaelis constant of **HES7** degra-dation **(*x axis*)**	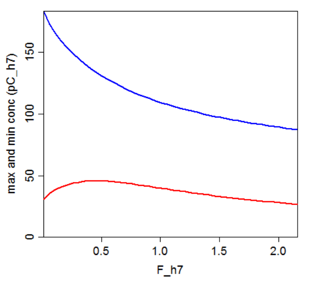	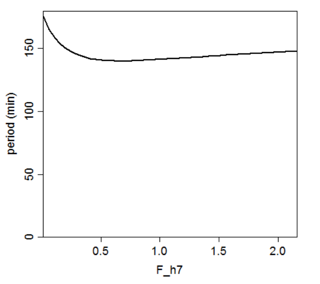	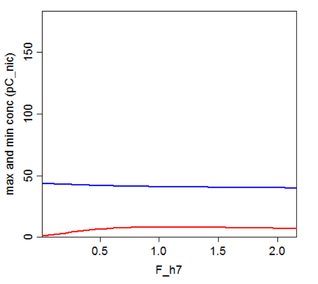	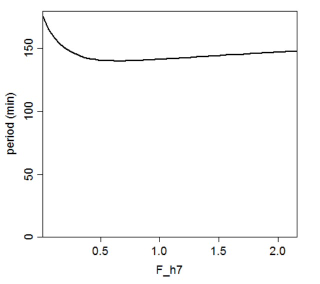
Maximal rate of **HES7** degra-dation **(*x axis*)**	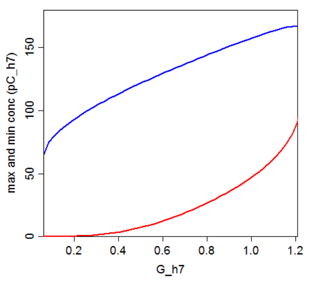	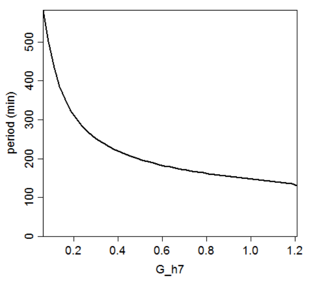	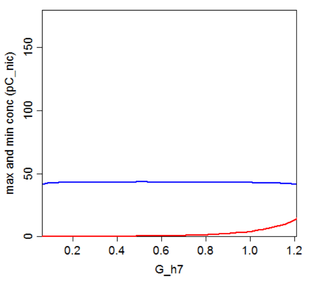	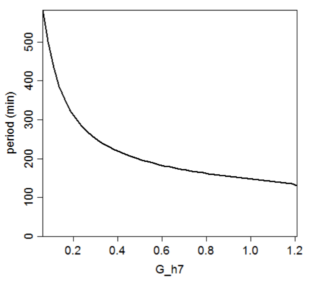

m: mRNA — **p**: protein — ___: minimal oscillation amplitude

mC: mRNA in cytoplasm — **pC**: protein in cytoplasm — ___: maximal oscillation amplitude

mN: mRNA in nucleus — **pN**: protein in nucleus — ___: time period in minutes

Maximum rate of NICD degradation = 5.0				
	**Minimum and maximum concentration of HES7 pC (*y axis*)**	**Oscillation period of HES7 pC (y axis)**	**Minimum and maximum concentration of NICD pC (*y axis*)**	**Oscillation period of NICD pC (y axis)**
**HES7** trans-lation rate **(*x axis*)**	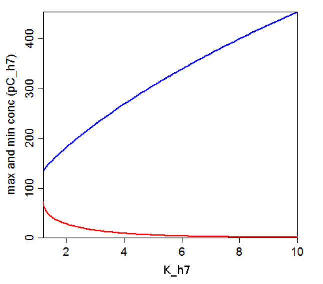	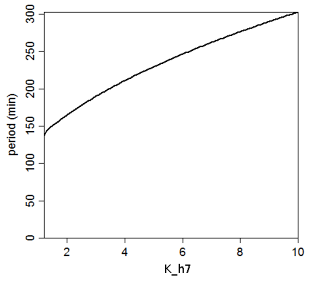	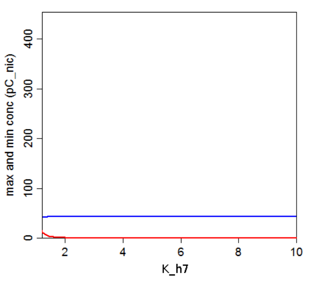	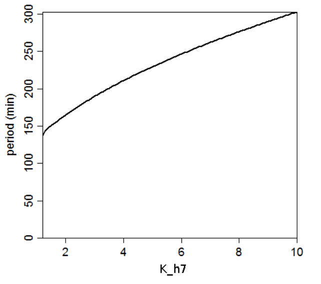
Hes7 trans-cription rate **(*x axis*)**	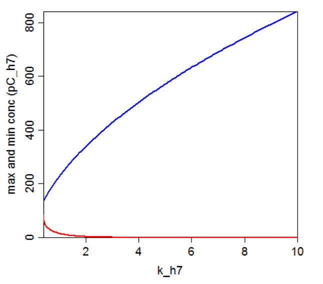	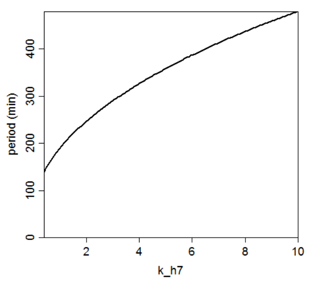	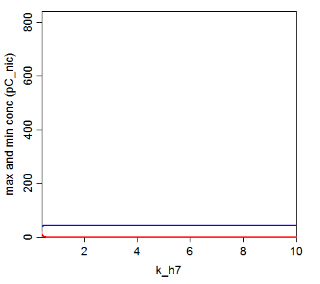	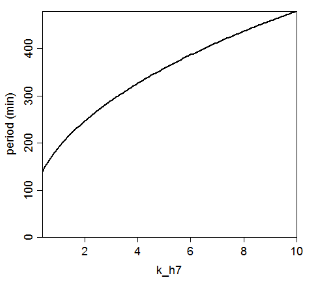
**NICD pC** decay **(*x axis*)**	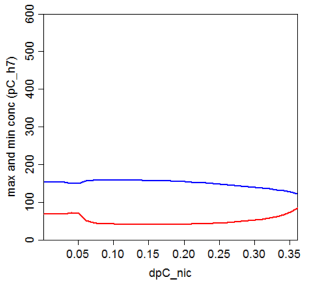	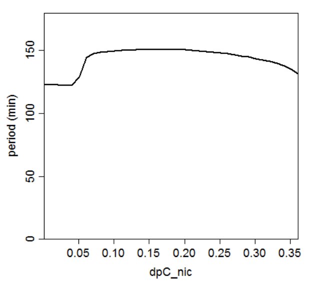	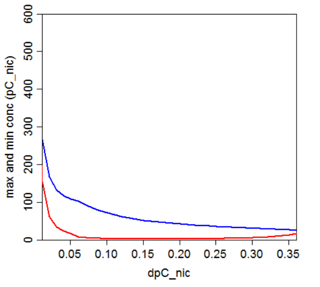	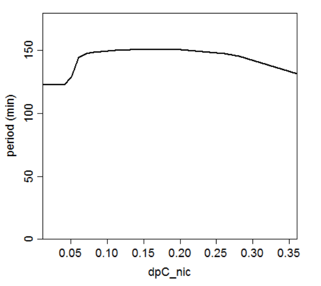
**NICD pC** export **(*x axis*)**	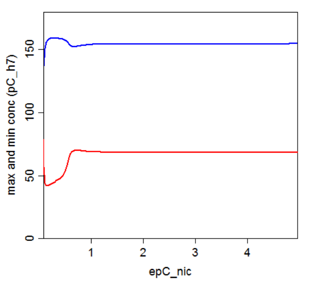	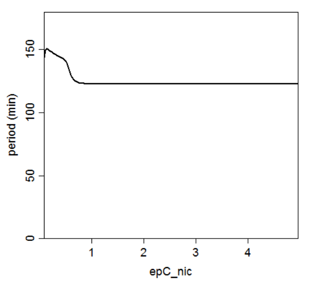	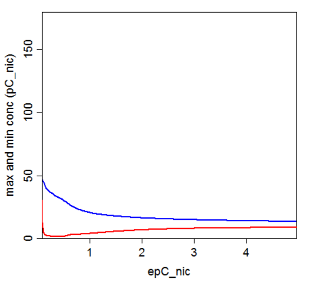	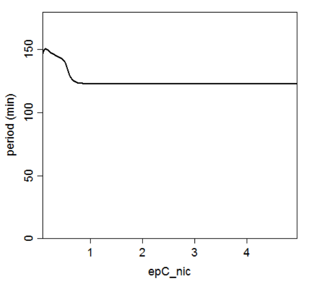

m: mRNA **p**: protein ___: minimal oscillation amplitude

mC: mRNA in cytoplasm **pC**: protein in cytoplasm ___: maximal oscillation amplitude

mN: mRNA in nucleus **pN**: protein in nucleus ___: time period in minutes

Maximum rate of NICD degradation = 5.0				
	**Minimum and maximum concentration of HES7 pC (*y axis*)**	**Oscillation period of HES7 pC (y axis)**	**Minimum and maximum concentration of NICD pC (*y axis*)**	**Oscillation period of NICD pC (y axis)**
**NICD pN** export **(*x axis*)**	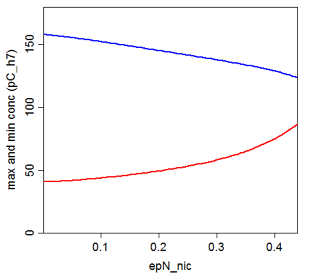	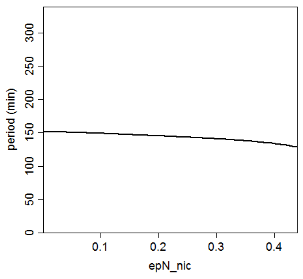	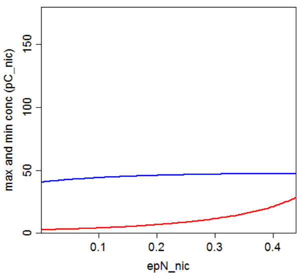	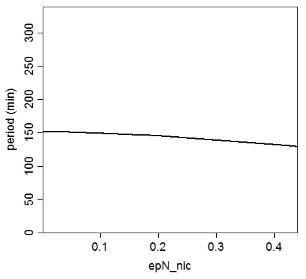
Michaelis constant of **NICD** degra-dation **(*x axis*)**	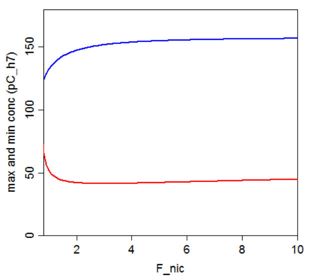	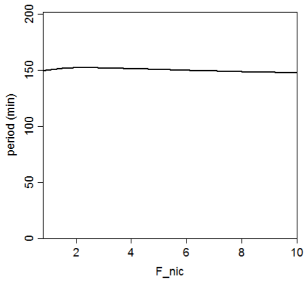	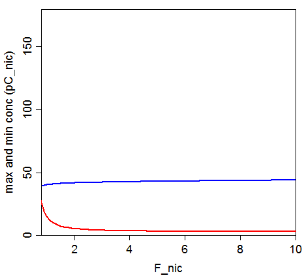	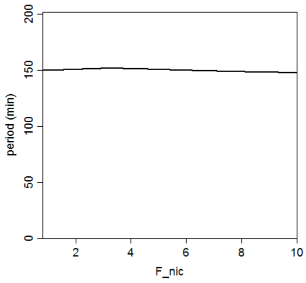
**NICD** *Hill threshold* **(*x axis*)**	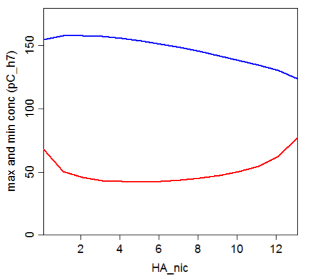	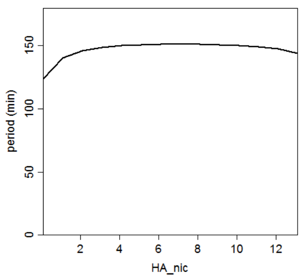	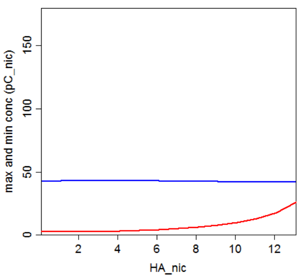	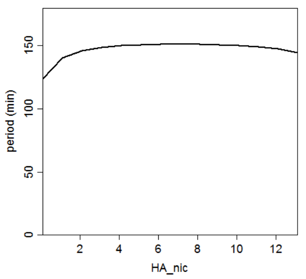
**Hes7** *Hill threshold* **(*x axis*)**	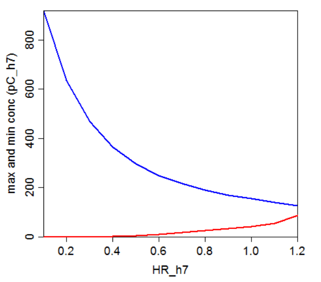	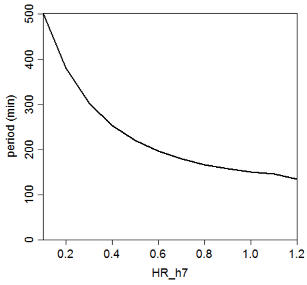	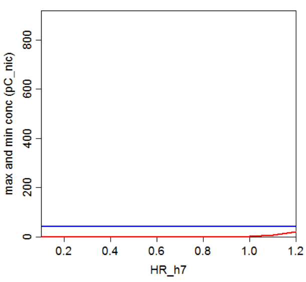	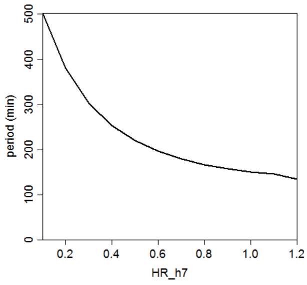

m: mRNA **p**: protein ___: minimal oscillation amplitude

mC: mRNA in cytoplasm **pC**: protein in cytoplasm ___: maximal oscillation amplitude

mN: mRNA in nucleus **pN**: protein in nucleus ___: time period in minutes

Maximal rate of **NICD** degradation ***(x axis)***	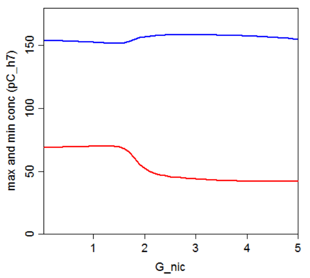	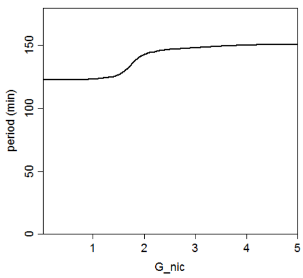	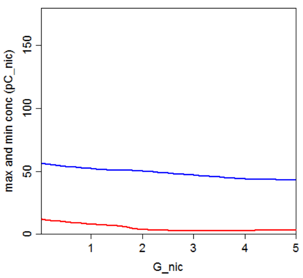	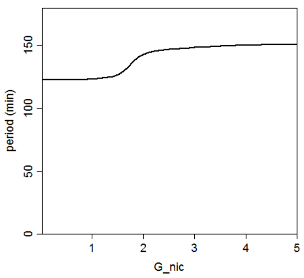

m: mRNA
mC: mRNA in cytoplasm
mN: mRNA in nucleus

p: protein
pC: protein in cytoplasm
pN: protein in nucleus

___: minimal oscillation amplitude
___: maximal oscillation amplitude
___: time period in minutes

(B)

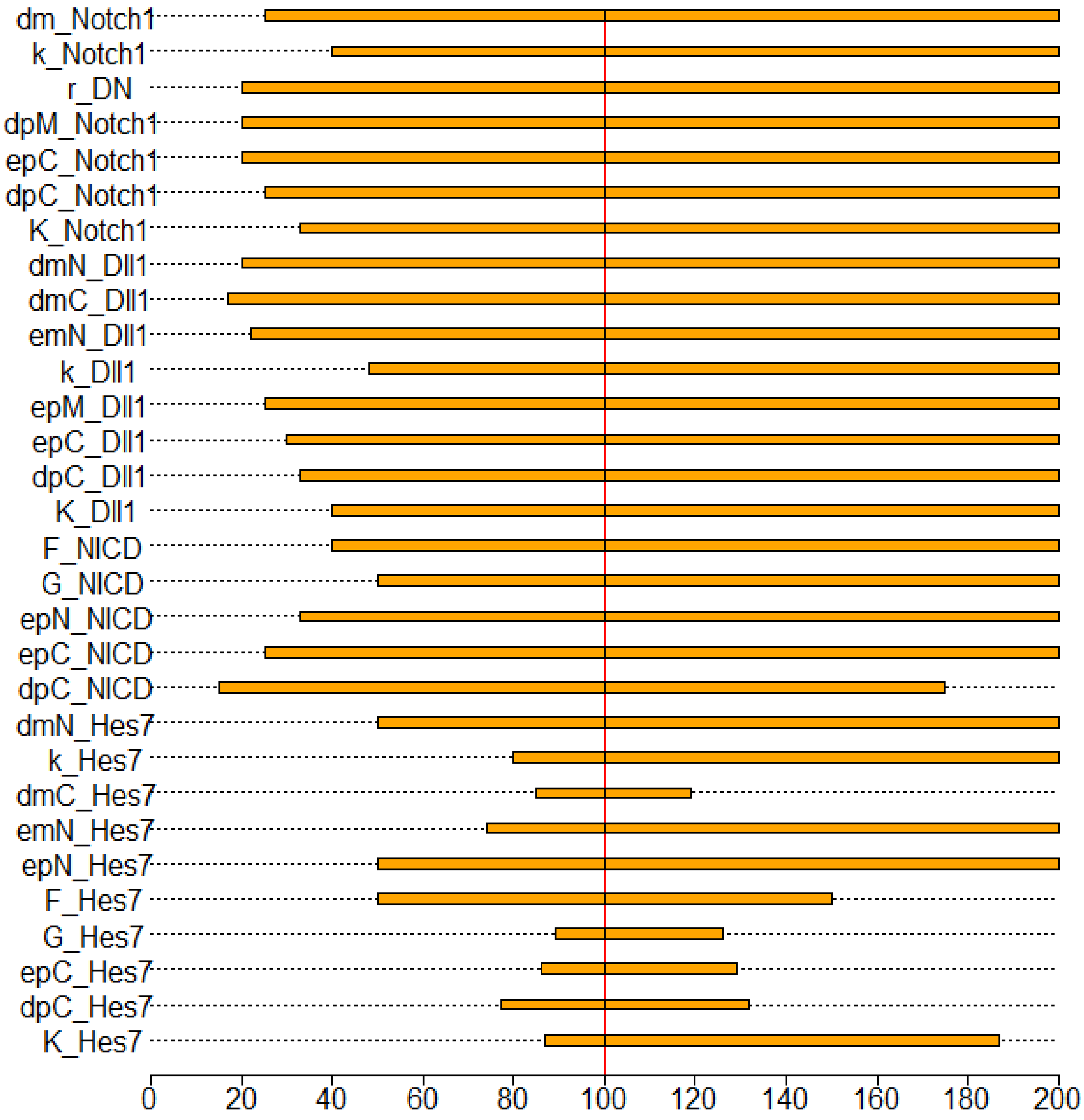

Parameter range with undamped oscillations.

(C)

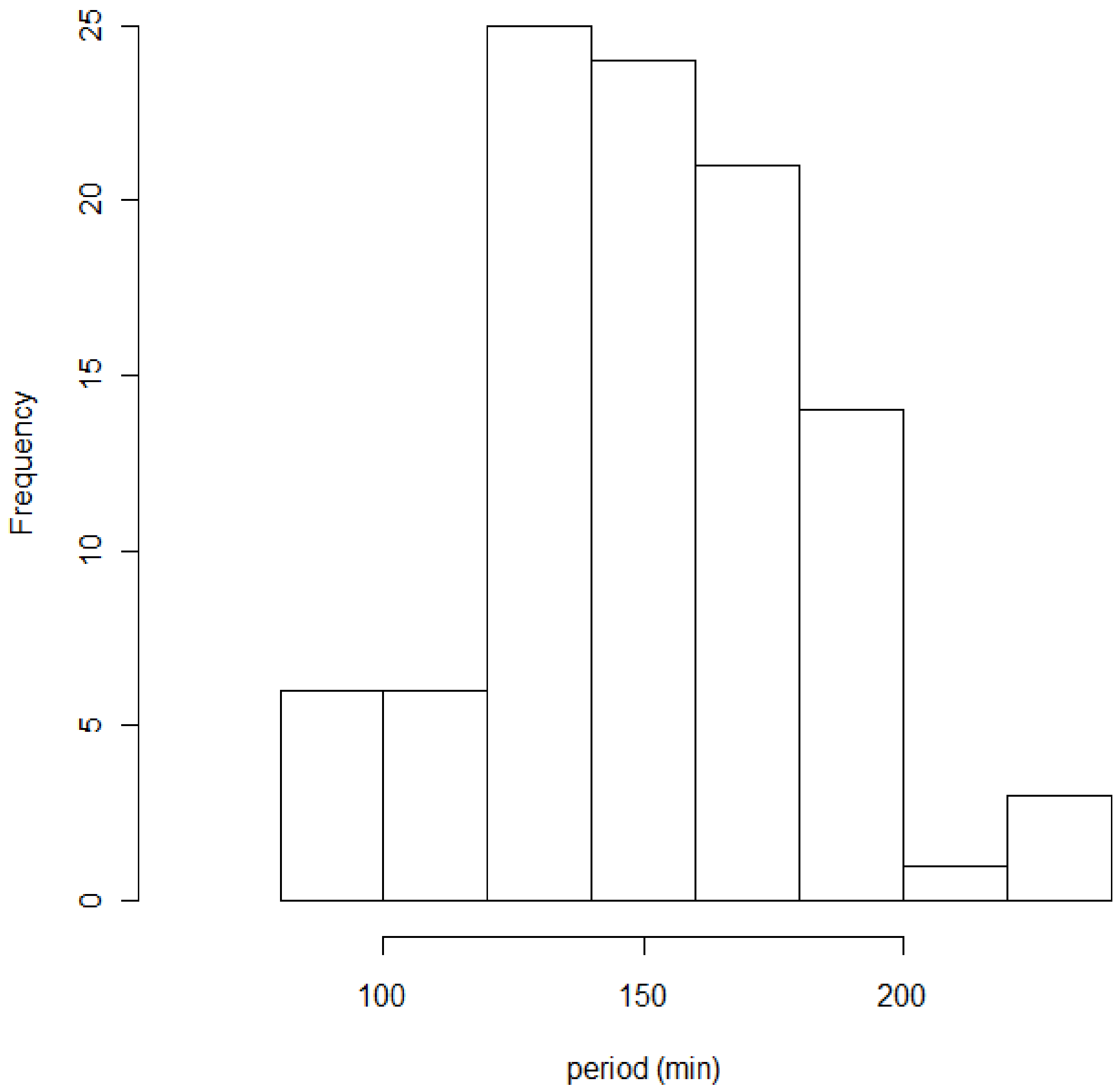

Distribution of period lengths for 100 parameter sets with parameters chosen at random from the ranges shown above.
