# Supplementary figures and images for: From Dynamic Expression Patterns to Boundary Formation in the Presomitic Mesoderm
Source: PLoS Comput Biol. 2012 Jun 28;8(6):e1002586. doi: 10.1371/journal.pcbi.1002586 (PMC3386180; doi:10.1371/journal.pcbi.1002586)

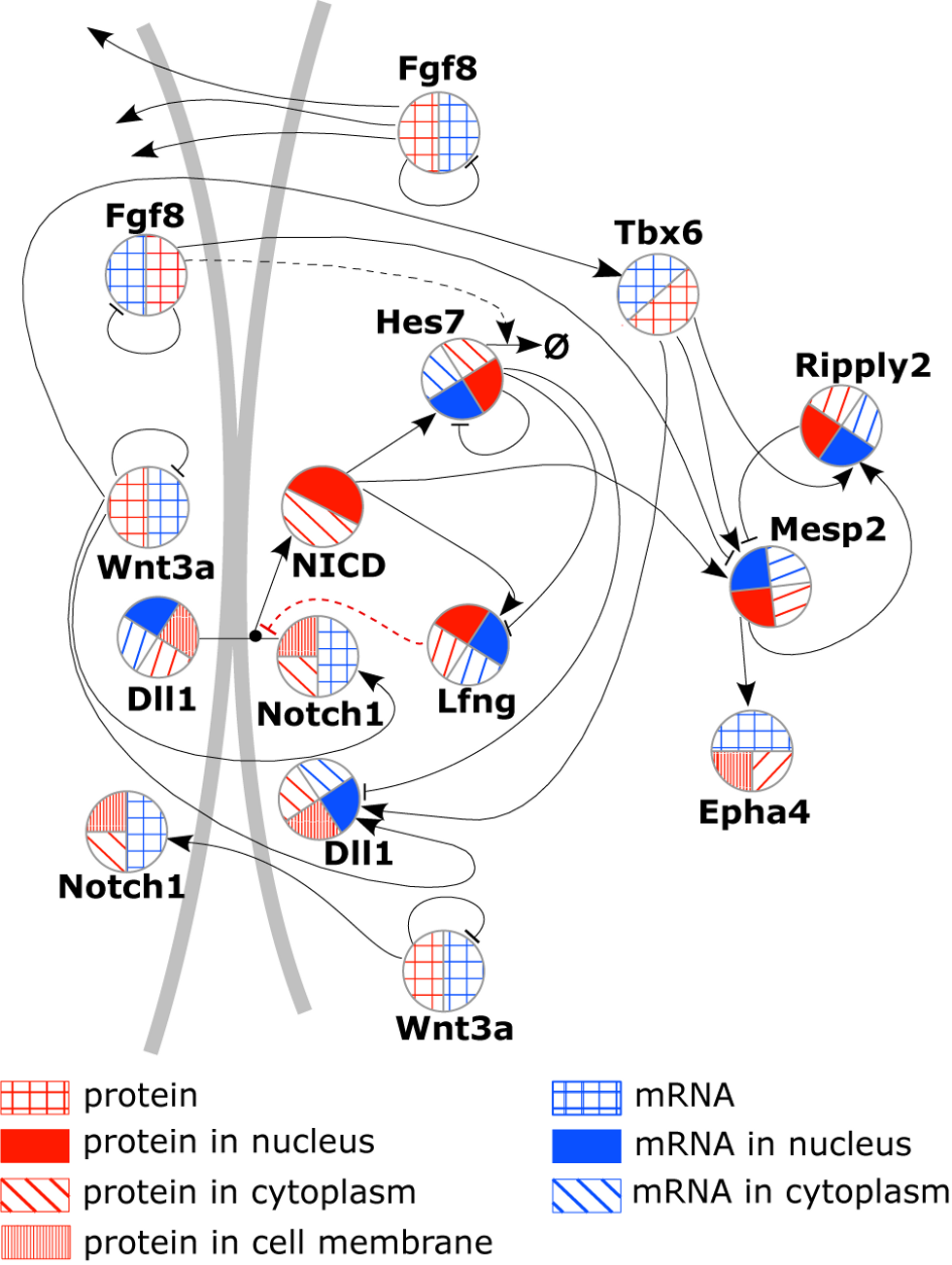

Supplement: Figure S1 — GRN for an alternative model in which FGF8 is coupled to the HES7 protein decay. (TIF) [file pcbi.1002586.s002.tif]

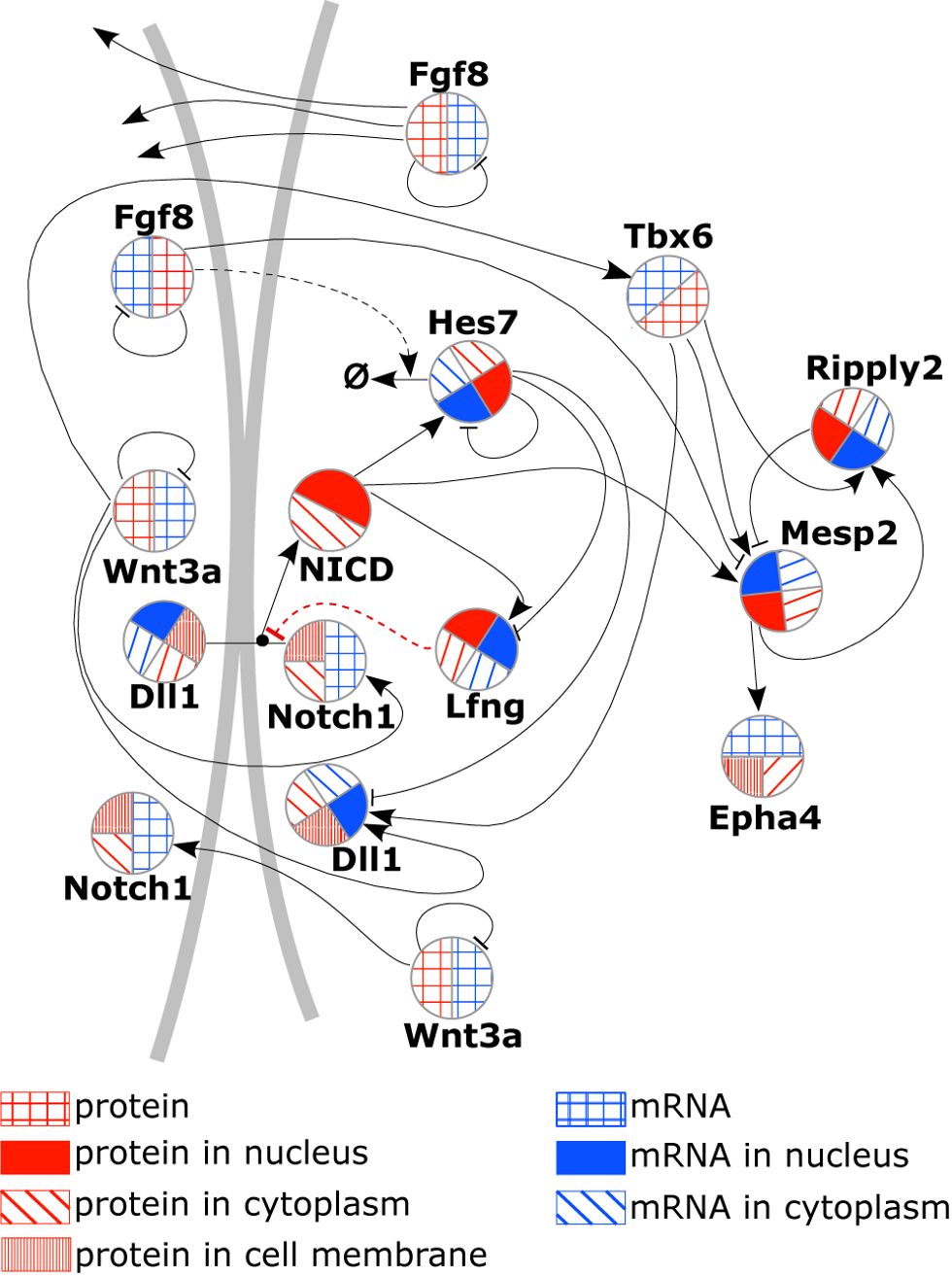

Supplement: Figure S2 — GRN for an alternative model in which FGF8 is coupled to the Hes7 mRNA decay. (TIF) [file pcbi.1002586.s003.tif]

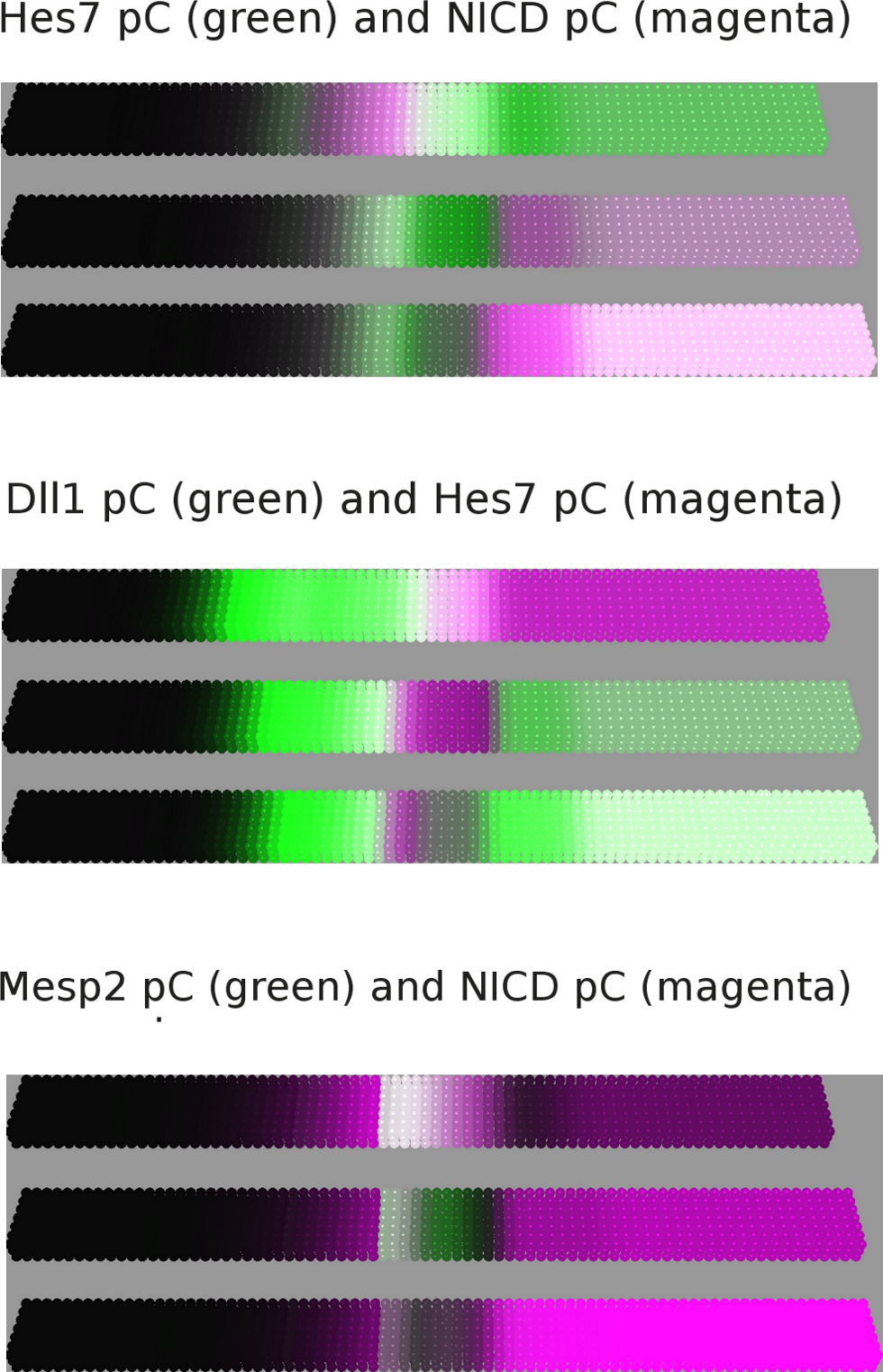

Supplement: Figure S3 — Virtual double staining. Snapshots at three different time points showing the double expression of cytoplasmic HES7 (green) and NICD (magenta) at the top, cytoplasmic DLL1 (green) and HES7 (magenta) in the middle, and cytoplasmic MESP2 (green) and NICD (magenta) at the bottom. As emissive colors were used, overlapping expression results in a white hue when the expression of both proteins is roughly equally strong. (TIF) [file pcbi.1002586.s004.tif]

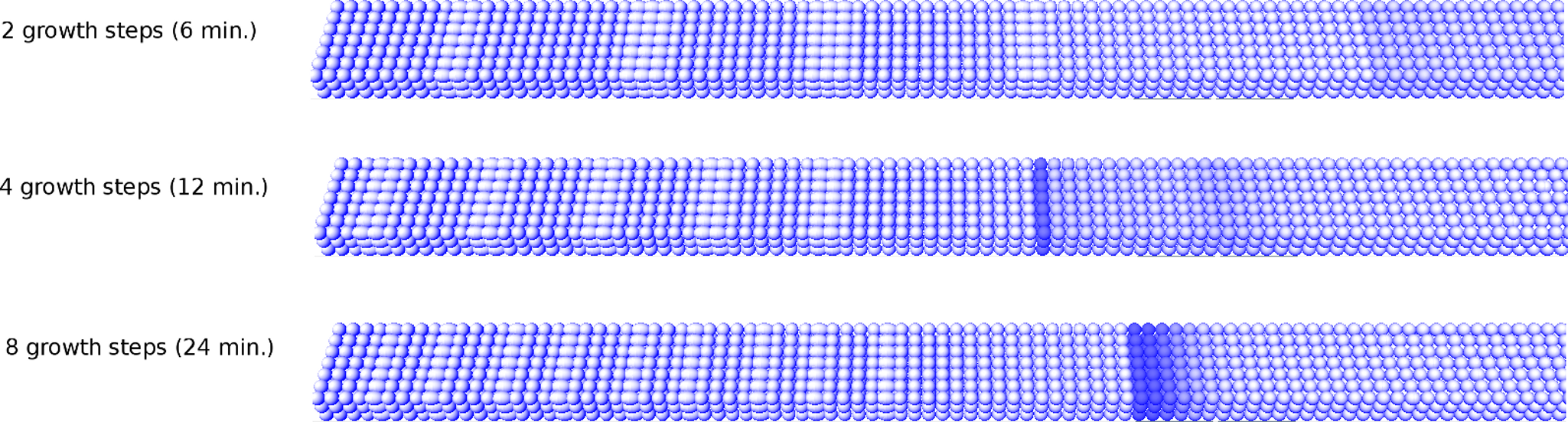

Supplement: Figure S4 — Variation of the PSM growth rate. Doubling and halving of the growth rate of the PSM results in doubling and halving of the length of a somite. Somite length is measured from the middle of one stripe of deformed cells to the middle of the next stripe. Shown is the Mesp2 expression. (TIF) [file pcbi.1002586.s005.tif]

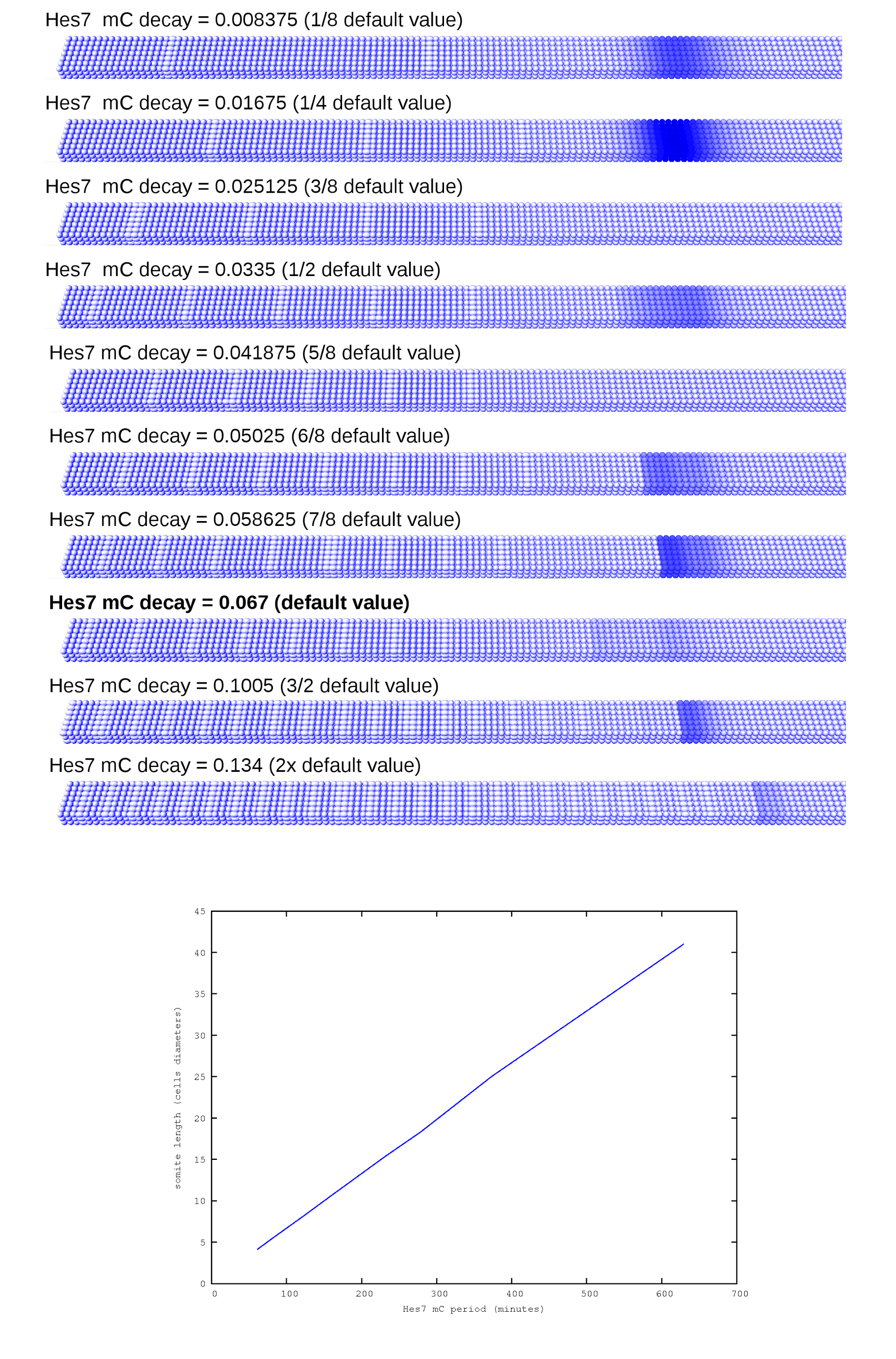

Supplement: Figure S5 — Variation of the clock rate by varying the HES7 decay rate. Varying the oscillation period by changing the cytoplasmic Hes7 mRNA decay rate and measuring the resulting variation in somite length, one observes a linear relationship between clock period and somite length, measured in cell numbers in the axial direction starting with and including the left deformed cell. The EPH4A threshold was set so high that mostly only one cell stripe deforms. To achieve clock periods smaller than the default case, not only the Hes7 mRNA decay rate had to be rescaled but also all other parameters of the differential equations, except those occurring in a denominator, which is equivalent to a rescaling of time. (TIF) [file pcbi.1002586.s006.tif]

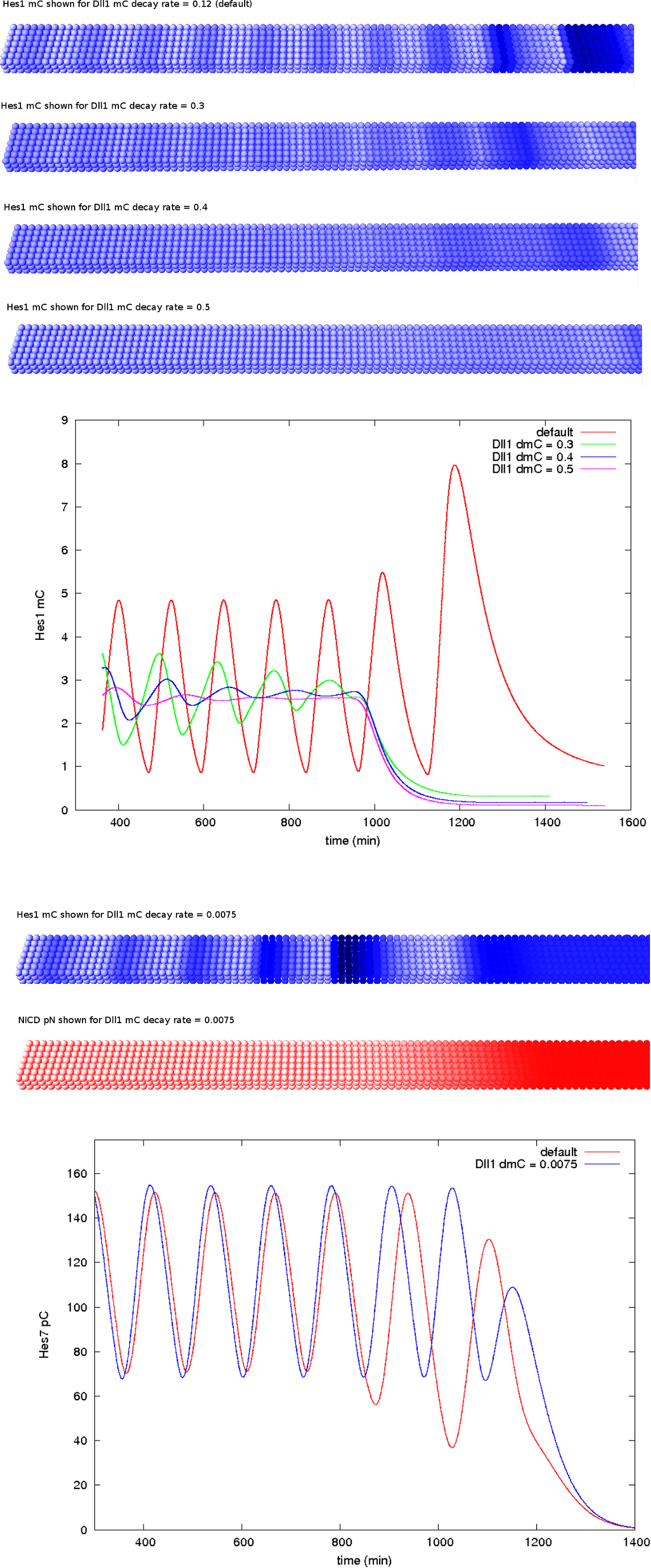

Supplement: Figure S6 — Varying the mRNA decay rate of Dll1 . Increasing the Dll1 mRNA decay rate leads to damped D/N signaling and consequently damped Hes1 oscillations (top panels and plot in the middle). Decreasing the Dll1 mRNA rate leads to roughly constant NICD expression (red panel). However, HES7 still oscillates due to the negative feedback on its own promoter (bottom plot). (TIF) [file pcbi.1002586.s007.tif]

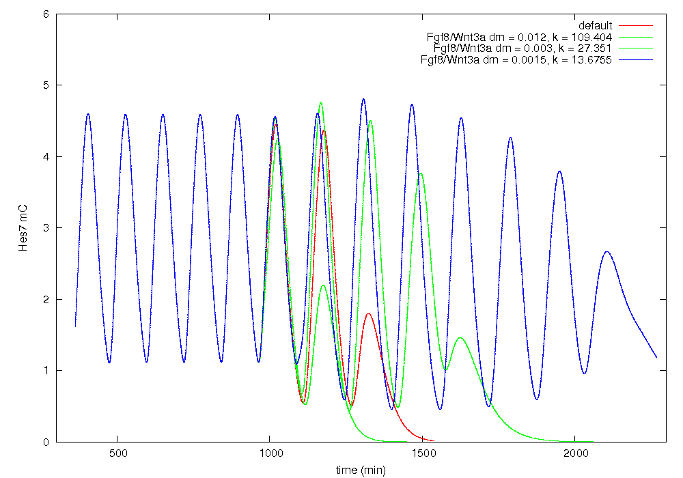

Supplement: Figure S7 — Number of oscillation periods for different gradient decay lengths. Shown are time courses for Hes7 oscillations in one cell when Fgf8 as well as Wnt3a mRNA decay rates are changed, i.e. the gradient is lengthened or shortened. The number of oscillations a cell executes before becoming part of a somite depends on gradient length. (TIF) [file pcbi.1002586.s008.tif]

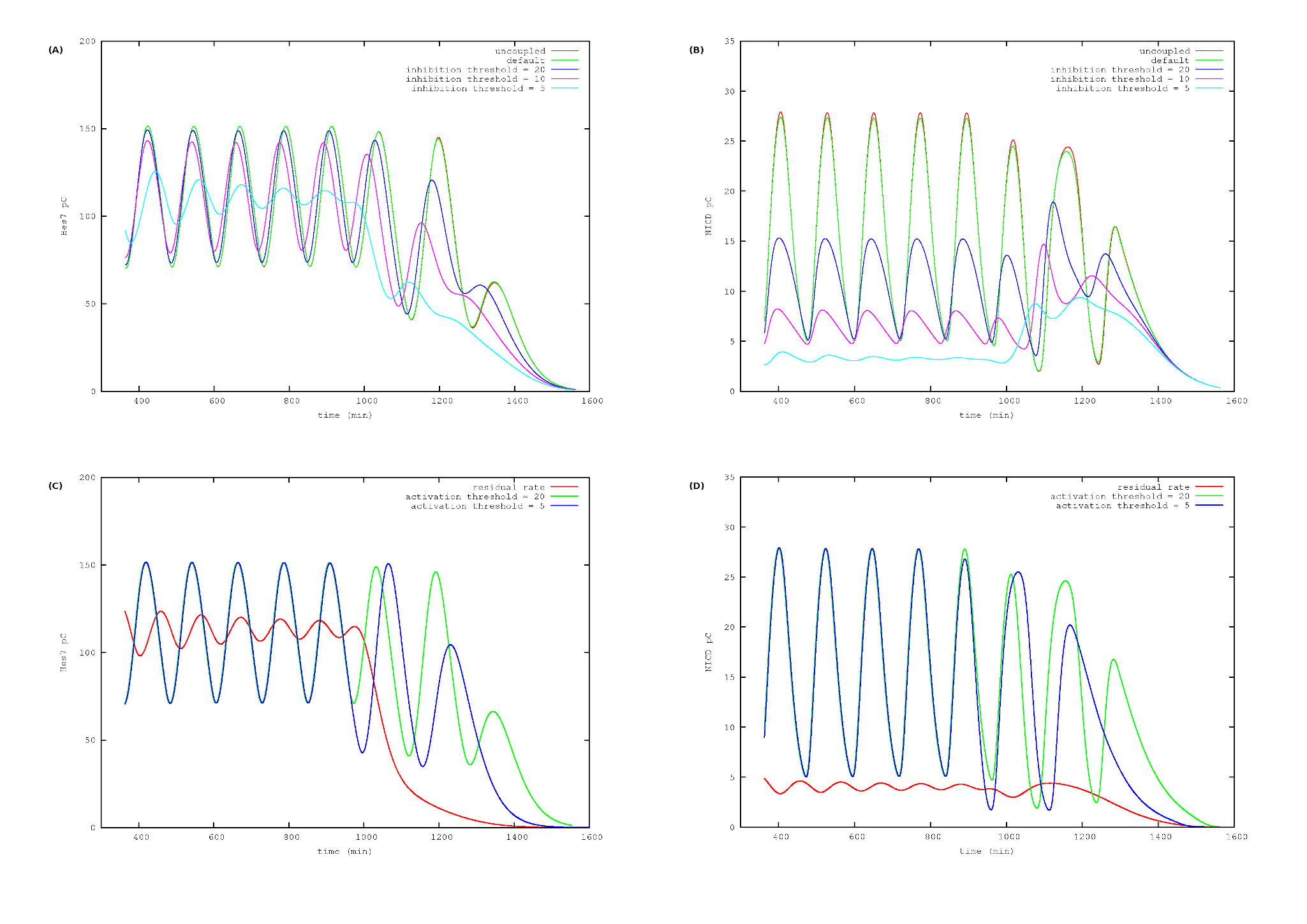

Supplement: Figure S8 — Results for modulation of D/N signaling by LFNG. Shown on the top row is the time course for cytoplasmic HES7 (left) and NICD (right) when the inhibition threshold in the Hill function reducing the D/N coupling between cells is lowered. This means, when the inhibition by LFNG is increased one observes an increasing damping of the oscillation amplitude. Shown on the bottom row is the time course for cytoplasmic HES7 (left) and NICD (right) with LFNG activating D/N signaling for two different threshold values in the activating function and the damped expression when activating LFNG action is abolished and only the very small residual rate of unmodified NOTCH1 binding to DLL1 is left. (TIF) [file pcbi.1002586.s009.tif]

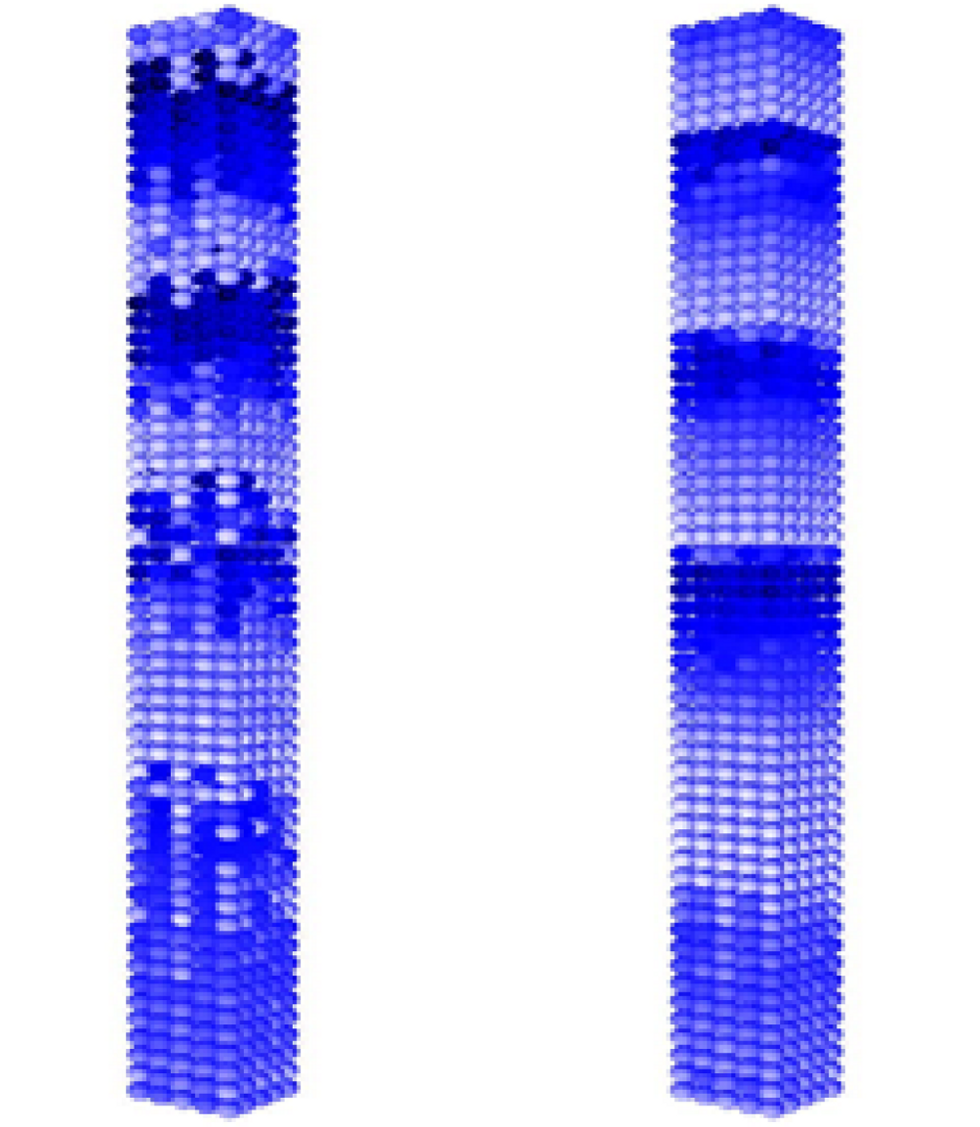

Supplement: Figure S9 — Snapshots of cytoplasmic Hes1 mRNA concentration without (left panel) and with (right panel) D/N synchronization. In this simplified model, Hes1 is the core oscillator and its mRNA decay is coupled to the FGF8 gradient. Here, the PSM growth zone comprises only one layer. Newborn cells start their oscillations with a random phase difference (maximally 25%). Color intensity in each cell indicates high (dark) or low (light) mRNA concentration. (TIF) [file pcbi.1002586.s010.tif]
