# Supplementary material for: From Dynamic Expression Patterns to Boundary Formation in the Presomitic Mesoderm
Source: PLoS Comput Biol. 2012 Jun 28;8(6):e1002586. doi: 10.1371/journal.pcbi.1002586 (PMC3386180; doi:10.1371/journal.pcbi.1002586)
Supplement: Table S1 — Parameter values and differential equations of the model. (PDF) [file pcbi.1002586.s011.pdf]

Gene Name	Differential Equations	Hill Function (H_h)	Initial Values	Parameter Values
**Hes7**	$\frac{d(pC(t))}{dt} = K\cdot mC(t) - dpC\cdot pC(t) - epC\cdot pC(t) + epN\cdot pN(t)$<br>$\frac{d(pN(t))}{dt} = epC\cdot pC(t) - \frac{G\cdot pN(t)}{F+pN(t)} - epN\cdot pN(t)$<br>$\frac{d(mC(t))}{dt} = emN\cdot mN(t) - dmC\cdot mC(t)$<br>$\frac{d(mN(t))}{dt} = k\cdot H_h(t) - dmN\cdot mN(t) - emN\cdot mN(t)$	$\frac{1}{1+pN^2_{Hes7}}\cdot\frac{(\frac{pN_{NICD}}{4.5})^2}{1+(\frac{pN_{NICD}}{4.5})^2}$	$pC(0) = 105.5$<br>$pN(0) = 0.53$<br>$mC(0) = 4.47$<br>$mN(0) = 12.47$	$K = 1.5$<br>$dpC = 0.031$<br>$epC = 0.007$<br>$epN = 0.001$<br>$G = 0.96$<br>$F = 0.2$<br>$emN = 0.038$<br>$dmC = 0.067$<br>$k = 0.5$<br>$dmN = 0.001$
**NICD**	$\frac{d(pC(t))}{dt} = r_{DN}\cdot ri_{Lfng}\cdot \overline{pM_{Dll1}}(t)\cdot pM_{Notch1}(t) - dpC\cdot pC(t) - epC\cdot pC(t) + epN\cdot pN(t)$<br>$\frac{d(pN(t))}{dt} = epC\cdot pC(t) - \frac{((G-base)\cdot(1-\frac{p_{Wnt3a}(t)}{p_{Wnt3a}(0)})+base)\cdot pN(t)}{F+pN(t)} - epN\cdot pN(t)$<br>$\overline{pM_{Dll1}}(t) = \frac{1}{n}\cdot\sum^{n} pM_{Dll1}(t)$, $n = number\ of\ neighbors$<br>$ri_{Lfng} = \frac{1}{1+(\frac{pC_{Lfng}(t)}{p_{crit}})^2}$<br>$ra_{Lfng} = r0 + \frac{(\frac{pC_{Lfng}(t)}{p_{crit}})^2}{1+(\frac{pC_{Lfng}(t)}{p_{crit}})^2}$ *replaces* ri_{Lfng} *when Lfng activates D/N signaling*<br>$r0 =$ *residual signaling without Lfng activation*		$pC(0) = 5$<br>$pN(0) = 5$	$r_{DN} = 0.05$<br>$dpC = 0.2$<br>$epC = 0.12$<br>$epN = 0.06$<br>$G = 5$<br>$base = 0.02$<br>$F = 5$<br>$p_{crit} = 170$<br>$r0 = 0.002$

Gene Name	Differential Equations	Hill Function (H_h)	Initial Values	Parameter Values
**Dll1**	$\frac{d(pM(t))}{dt} = epC\cdot pC(t) - epM\cdot pM(t) - dpM\cdot pM(t) - r_{DN}\cdot ri_{Lfng}\cdot \overline{pM_{Notch1}}(t)\cdot pM(t)$<br>$\frac{d(pC(t))}{dt} = K\cdot mC(t) - dpC\cdot pC(t) - epC\cdot pC(t) + epM\cdot pM(t)$<br>$\frac{d(mC(t))}{dt} = emN\cdot mN - dmC\cdot mC(t)$<br>$\frac{d(mN(t))}{dt} = k\cdot H_h(t) - dmN\cdot mN(t) - emN\cdot mN(t)$<br>$\overline{pM_{Notch1}}(t) = \frac{1}{n}\cdot\sum^{n} pM_{Notch1}(t)$, n = number of neighbors<br>$ri_{Lfng} = \frac{1}{1+\left(\frac{pC_{Lfng}(t)}{p_{crit}}\right)^2}$<br>$ra_{Lfng} = r0 + \frac{\left(\frac{pC_{Lfng}(t)}{p_{crit}}\right)^2}{1+\left(\frac{pC_{Lfng}(t)}{p_{crit}}\right)^2}$ replaces ri_{Lfng} when Lfng activates D/N signaling<br>$r0$ = residual signaling without Lfng activation	$\frac{1}{1+pN_{Hes7}^2}\cdot\frac{\left(\frac{p_{Tbx6}}{10}\right)^3}{1+\left(\frac{p_{Tbx6}}{10}\right)^3}\cdot\frac{p_{Wnt3a}}{1+p_{Wnt3a}}$	$pC(0) = 5$<br>$pM(0) = 5$<br>$mC(0) = 1$<br>$mN(0) = 5$	$K = 1.5$<br>$dpC = 0.09$<br>$epC = 0.1$<br>$epM = 0.1$<br>$dpM = 0$<br>$r_{DN} = 0.05$<br>$dmC = 0.12$<br>$emN = 0.09$<br>$k = 1.25$<br>$dmN = 0.001$<br>$p_{crit} = 170$<br>$r0 = 0.002$
**Notch1**	$\frac{d(pM(t))}{dt} = epC\cdot pC(t) - epM\cdot pM(t) - dpM\cdot pM(t) - r_{DN}\cdot ri_{Lfng}\cdot \overline{pM_{Dll1}}(t)\cdot pM(t)$<br>$\frac{d(pC(t))}{dt} = K\cdot m(t) - dpC\cdot pC(t) - epC\cdot pC(t) + epM\cdot pM(t)$<br>$\frac{d(m(t))}{dt} = k\cdot H_h(t) - dm\cdot m(t)$<br>$\overline{pM_{Dll1}}(t) = \frac{1}{n}\cdot\sum^{n} pM_{Dll1}(t)$, n = number of neighbors<br>$ri_{Lfng} = \frac{1}{1+\left(\frac{pC_{Lfng}(t)}{p_{crit}}\right)^2}$<br>$ra_{Lfng} = r0 + \frac{\left(\frac{pC_{Lfng}(t)}{p_{crit}}\right)^2}{1+\left(\frac{pC_{Lfng}(t)}{p_{crit}}\right)^2}$ replaces ri_{Lfng} when Lfng activates D/N signaling<br>$r0$ = residual signaling without Lfng activation	$\frac{\left(\frac{p_{Wnt3a}}{10}\right)^3}{1+\left(\frac{p_{Wnt3a}}{10}\right)^3}$	$pC(0) = 10$<br>$pM(0) = 5$<br>$m(0) = 10$	$K = 1.5$<br>$dpC = 0.2$<br>$epC = 0.1$<br>$epM = 0.0$<br>$dpM = 0.1$<br>$r_{DN} = 0.05$<br>$dm = 0.02$<br>$k = 0.5$<br>$p_{crit} = 170$<br>$r0 = 0.002$

Gene Name	Differential Equations	Hill Function (H_h)	Initial Values	Parameter Values
**Tbx6**	$\frac{d(p(t))}{dt} = K \cdot m(t) - dp \cdot p(t)$ $\frac{d(m(t))}{dt} = k \cdot H_h(t) - dm \cdot m(t)$	$\frac{(\frac{p_{Wnt3a}}{11})^3}{1+(\frac{p_{Wnt3a}}{11})^3}$	$p(0) = 150$ $m(0) = 20$	$K = 1.5$ $dp = 0.2$ $dm = 0.02$ $k = 0.5$
**Fgf8**	$\frac{d(p(t))}{dt} = K \cdot m(t) - dp \cdot p(t)$ $\frac{d(m(t))}{dt} = k \cdot H_h(t) - dm \cdot m(t)$	$\frac{1}{1+p^2}$	$p(0) = 45$ $m(0) = 4.5$	$K = 0.3$ $dp = 0.03$ $dm = 0.006$ $k = \begin{matrix} 54.702, growth\ zone \\ 0, outside\ growth\ zone \end{matrix}$
**Wnt3a**	$\frac{d(p(t))}{dt} = K \cdot m(t) - dp \cdot p(t)$ $\frac{d(m(t))}{dt} = k \cdot H_h(t) - dm \cdot m(t)$	$\frac{1}{1+p^2}$	$p(0) = 45$ $m(0) = 4.5$	$K = 0.3$ $dp = 0.03$ $dm = 0.006$ $k = \begin{matrix} 54.702, growth\ zone \\ 0, outside\ growth\ zone \end{matrix}$
**Mesp2**	$\frac{d(pC(t))}{dt} = K \cdot mC(t) - dpC \cdot pC(t) - epC \cdot pC(t) + epN \cdot pN(t)$ $\frac{d(pN(t))}{dt} = epC \cdot pC(t) - \frac{G \cdot pN(t)}{F + pN(t)} - epN \cdot pN(t)$ $\frac{d(mC(t))}{dt} = emN \cdot mN - dmC \cdot mC(t)$ $\frac{d(mN(t))}{dt} = k \cdot H_h(t) - dmN \cdot mN(t) - emN \cdot mN(t)$	$\frac{(\frac{pN_{NICD}}{5})^2}{1+(\frac{pN_{NICD}}{5})^2} \cdot \frac{(\frac{p_{Tbx6}}{10})^2}{1+(\frac{p_{Tbx6}}{10})^2} \cdot \frac{1}{1+(\frac{p_{Fgf8}}{11})^4} \cdot \frac{1}{1+pN_{Ripply2}^{2}}$	$pC(0) = 0$ $pN(0) = 0$ $mC(0) = 0$ $mN(0) = 0$	$K = 1.5$ $dpC = 0.031$ $epC = 0.1$ $epN = 0.001$ $G = 0.96$ $F = 0.2$ $emN = 0.05$ $dmC = 0.067$ $k = 0.5$ $dmN = 0.001$
**Ripply2**	$\frac{d(pC(t))}{dt} = K \cdot mC(t) - dpC \cdot pC(t) - epC \cdot pC(t) + epN \cdot pN(t)$ $\frac{d(pN(t))}{dt} = epC \cdot pC(t) - \frac{G \cdot pN(t)}{F + pN(t)} - epN \cdot pN(t)$ $\frac{d(mC(t))}{dt} = -dmC \cdot mC(t) + emN \cdot mN$ $\frac{d(mN(t))}{dt} = k \cdot H_h(t) - dmN \cdot mN(t) - emN \cdot mN(t)$	$\frac{pN^2{}_{Mesp2}}{1+pN^2{}_{Mesp2}} \cdot \frac{\frac{p_{Tbx6}}{10}}{1+\frac{p_{Tbx6}}{10}}$	$pC(0) = 0$ $pN(0) = 0$ $mC(0) = 0$ $mN(0) = 0$	$K = 1.5$ $dpC = 0.031$ $epC = 0.05$ $epN = 0.001$ $G = 0.96$ $F = 0.2$ $emN = 0.038$ $dmC = 0.067$ $k = 0.5$ $dmN = 0.001$

Gene Name	Differential Equations	Hill Function (H_h)	Initial Values	Parameter Values
**Hes1**	$\frac{d(pC(t))}{dt} = K\cdot mC(t) - dpC\cdot pC(t) - epC\cdot pC(t) + epN\cdot pN(t)$<br>$\frac{d(pN(t))}{dt} = epC\cdot pC(t) - \frac{G\cdot pN(t)}{F+pN(t)} - epN\cdot pN(t)$<br>$\frac{d(mC(t))}{dt} = emN\cdot mN(t) - \frac{p_{Fgf8}(t)}{p_{Fgf8}(0)}\cdot dmC\cdot mC(t)$<br>$\frac{d(mN(t))}{dt} = k\cdot H_h(t) - dmN\cdot mN(t) - emN\cdot mN(t)$	$\frac{1}{1+pN^3_{Hes1}}\cdot\frac{(\frac{pN_{NICD}}{4.5})^2}{1+(\frac{pN_{NICD}}{4.5})^2}$	$pC(0) = 105.5$<br>$pN(0) = 0.53$<br>$mC(0) = 4.47$<br>$mN(0) = 12.47$	$K = 1.5$<br>$dpC = 0.031$<br>$epC = 0.007$<br>$epN = 0.001$<br>$G = 0.96$<br>$F = 0.2$<br>$emN = 0.038$<br>$dmC = 0.067$<br>$k = 0.5$<br>$dmN = 0.001$
**Lfng**	$\frac{d(pC(t))}{dt} = K\cdot mC(t) - dpC\cdot pC(t) - epC\cdot pC(t) + epN\cdot pN(t)$<br>$\frac{d(pN(t))}{dt} = epC\cdot pC(t) - \frac{G\cdot pN(t)}{F+pN(t)} - epN\cdot pN(t)$<br>$\frac{d(mC(t))}{dt} = emN\cdot mN(t) - dmC\cdot mC(t)$<br>$\frac{d(mN(t))}{dt} = k\cdot H_h(t) - dmN\cdot mN(t) - emN\cdot mN(t)$	$\frac{1}{1+pN^2_{Hes7}}\cdot\frac{(\frac{pN_{NICD}}{4.5})^2}{1+(\frac{pN_{NICD}}{4.5})^2}$	$pC(0) = 0$<br>$pN(0) = 0$<br>$mC(0) = 0$<br>$mN(0) = 0$	$K = 1.5$<br>$dpC = 0.031$<br>$epC = 0$<br>$epN = 0$<br>$G = 0.96$<br>$F = 0.2$<br>$emN = 0.038$<br>$dmC = 0.067$<br>$k = 0.5$<br>$dmN = 0.001$
**Epha4**	$\frac{d(pM(t))}{dt} = epC\cdot pC(t) - epM\cdot pM(t)$<br>$\frac{d(pC(t))}{dt} = K\cdot m(t) - dpC\cdot pC(t) - epC\cdot pC(t)$<br>$\frac{d(m(t))}{dt} = k\cdot H_h(t) - dm\cdot m(t)$	$\frac{(\frac{pN_{Mesp2}}{10})^2}{1+(\frac{pN_{Mesp2}}{10})^2}$	$pC(0) = 0$<br>$pM(0) = 0$<br>$m(0) = 0$	$K = 1.5$<br>$dpC = 0$<br>$epC = 0.1$<br>$epM = 0.0$<br>$dm = 0.02$<br>$k = 0.5$
