# Supplementary material for: From Dynamic Expression Patterns to Boundary Formation in the Presomitic Mesoderm
Source: PLoS Comput Biol. 2012 Jun 28;8(6):e1002586. doi: 10.1371/journal.pcbi.1002586 (PMC3386180; doi:10.1371/journal.pcbi.1002586)
Supplement: Text S1 — Mini manual for the simulation program. (PDF) [file pcbi.1002586.s012.pdf]

From Dynamic Expression Patterns to Boundary Formation in the Presomitic Mesoderm

Authors: Hendrik B. Tiedemann, Elida Schneltzer

Simulation Program Mini-Manual

System Requirements

We implemented our simulation program as a Java application on a dual core processor machine using jdk1.6 and Java3D version 1.5.2. To run it we use an amount of 1500MB of the memory allocation pool (java virtual machine options -Xms1500m -Xmx1500m).
We tested it on

- **Ubuntu 10.04 LTS - *Lucid Lynx*** with
  java version "1.6.0_20"
  OpenJDK Runtime Environment (IcedTea6 1.9.13)
  (6b20-1.9.13-0ubuntu1~10.04.1)
  OpenJDK 64-Bit Server VM (build 19.0-b09, mixed mode)
  and the **Java 3D™ 1.5.2 Release**.
- **Microsoft Windows XP Professional Version 2002 Service Pack 2** with
  java version "1.6.0_21"
  Java(TM) SE Runtime Environment (build 1.6.0_21-b07)
  Java HotSpot(TM) Client VM (build 17.0-b17, mixed mode, sharing)
  an the **Java 3D™ 1.5.1 Release**.

It appears that the Material colors implemented in Java3D, except diffuse, are ignored when rendering under Mac OS X 10.5 and later.

Installation and Starting the Application

To run the program you need to install the packed file SIM.zip, which can be downloaded from www.helmholtz-muenchen.de/en/ieg/downloads/simulation11.
After unpacking the file you get a directory (*SIM/*) with the following configuration:

- Subdirectory *ICONS/* contains figures used to design the graphical user interface of the simulation program.
- Subdirectory *sim_lib/* contains all supplementary jar files used by the application.
- Subdirectory *sim_model/* contains the standard configuration files for the gene regulatory networks mentioned in the publication and the supplementary material: *Fgf8_on_Hes1_dmC_Wnt3a_on_NICD_dpN/configfile* corresponds to the model shown in **Figure 2** (publication), while subdirectories *Fgf8_on_Hes7_dpC/* and *Fgf8_on_Hes7_dmC/* contain configuration files for the models shown in **Figure S1** and **Figure S2** (supplementary material). C*onfigfile* saves the parameter values needed to start the application.
- Jar file *sim.jar* contains the simulation program.
- Executable batch file *startSIM.bat* starts the application on **Windows**.
- Executable shell script *startSim.sh* starts the application on **Linux** and **MacOS**.

Execute *startSim.bat* to start the program on **Windows** and *./startSim.sh* on **Linux** and **MacOS**. For more details read thefile *SIM/README*.

Starting the Simulation

The application starts with a graphical user interface, on which the user can see and change the parameter values needed to start the simulation. The buttons to start and stop the simulation are on the bar at the bottom of the graphical user interface (GUI):

- By clicking on **'*start*'** the simulation starts with the configuration status described on the parameter panel (see chapter **Using the Graphical User Interface**).
- Button **'*cancel*'** terminates the Java application.
- It is possible to return from the simulation panel to the parameter panel by clicking **'*return*'**.

Using the Graphical User Interface (GUI)

The picture below shows the upper half part of the GUI from which the simulation can be started. It contains informations about the genes building the gene regulatory network (GRN) and its interactions.

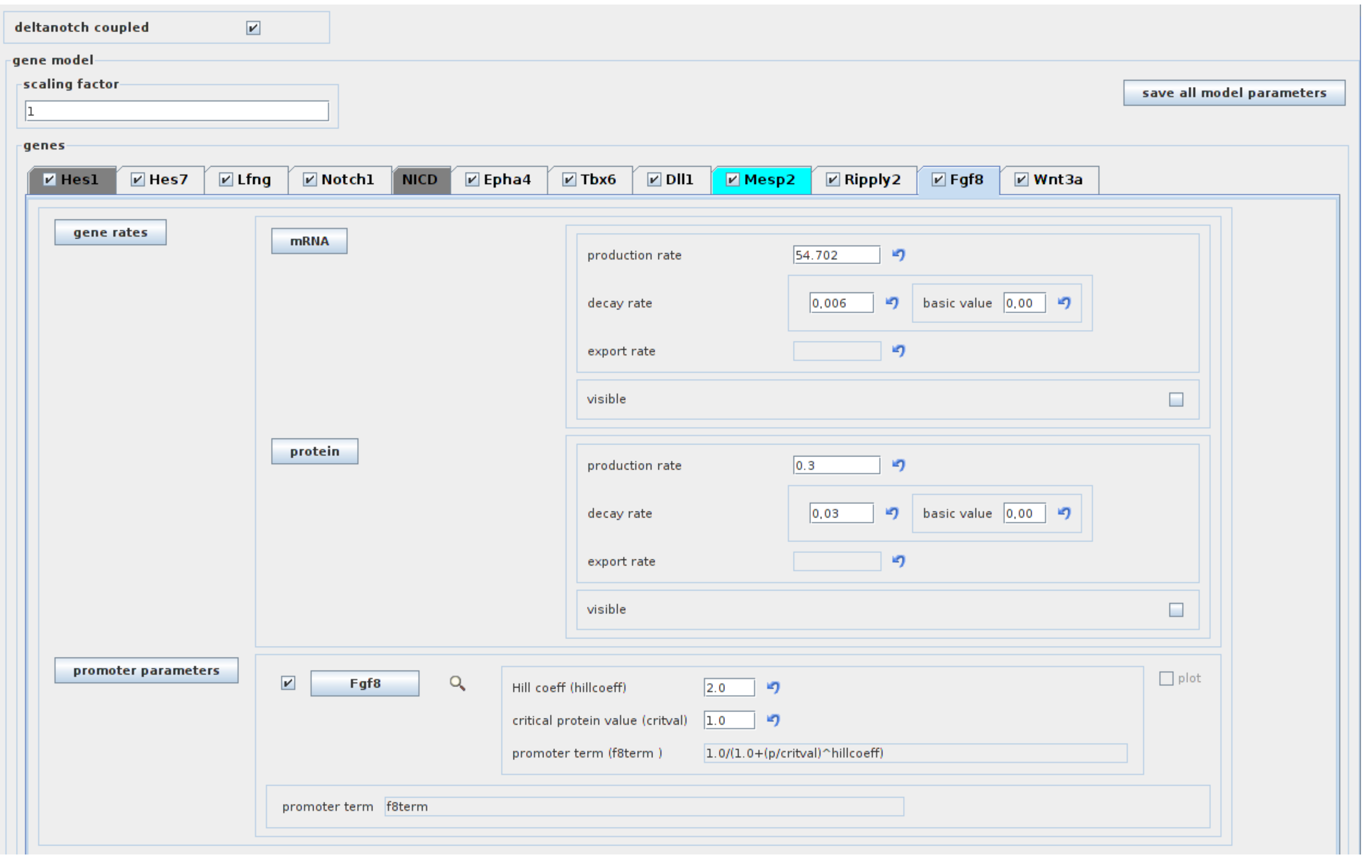

- Delta/Notch interaction between cells can be turned ***on*** or ***off*** through the '***deltanotch coupled***' check box. The pictures below show an example for what the check box selection means:
    1. Turning it ***on*** (as below) all promoter parts of genes in the GRN containing NICD binding sides are activated.

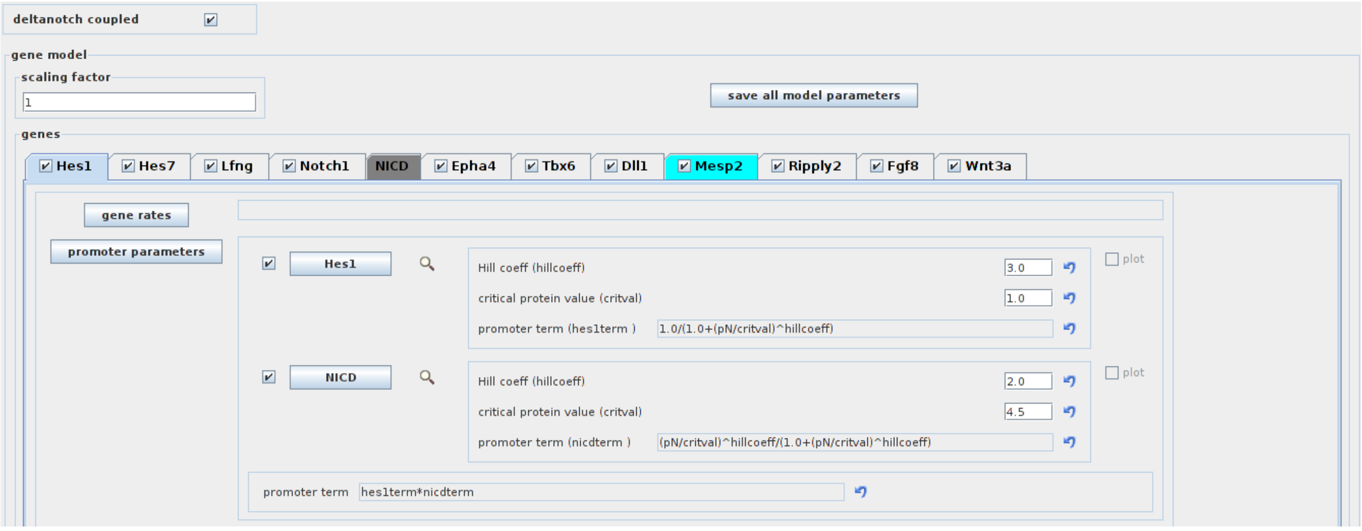

2. Turning it ***off*** (as below) has the consequence that all promoter parts

containing NICD binding sides are also switched off.

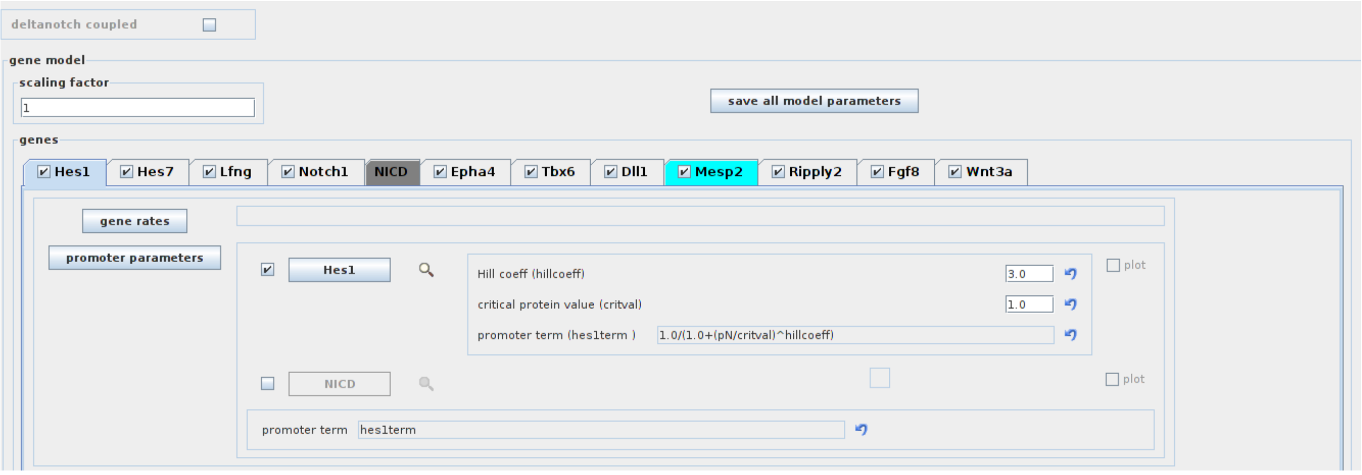

- The value of the '***scaling factor***' text field scales all parameters by a factor. This is equivalent to scaling by time. To change the **scaling factor** proceed as described in the following:
    1. Change the text field value (e.g. to 2) and press *ENTER* to continue.

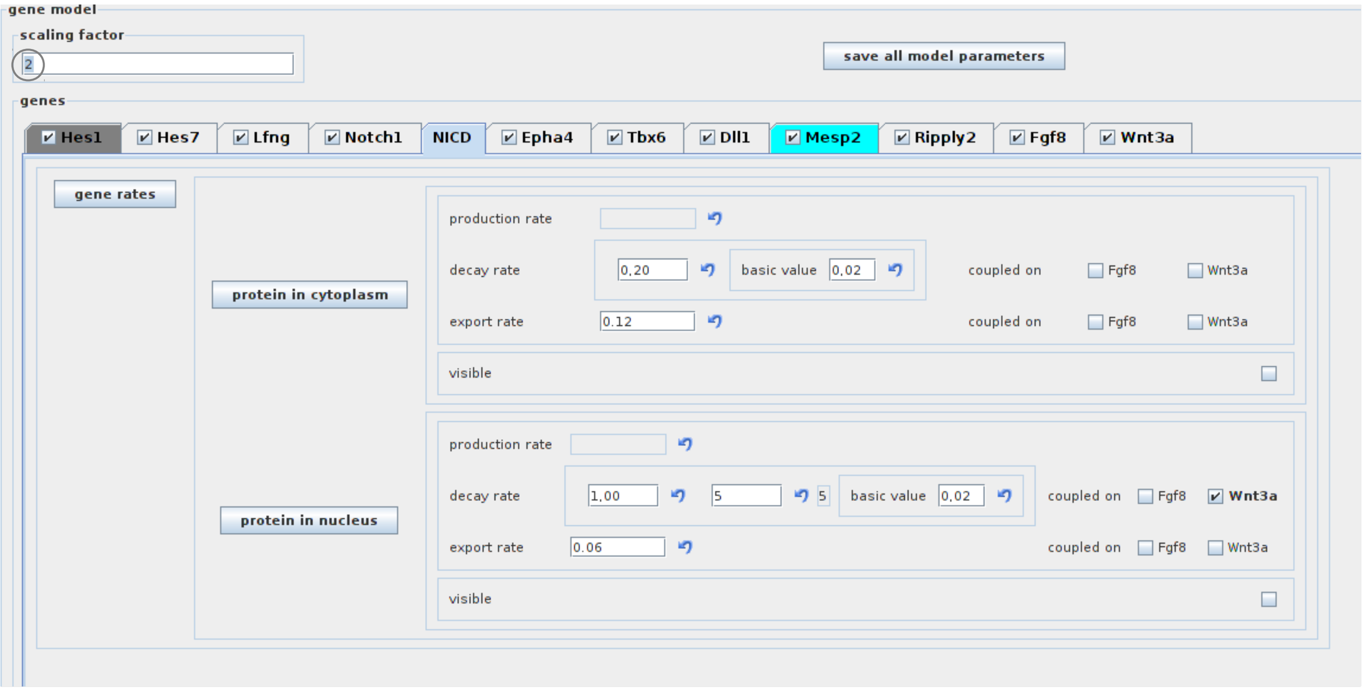

2. A pop-up window opens to inform you what will be the consequence of changing the value. To continue click ***OK*** or just close the window if nothing should happen.

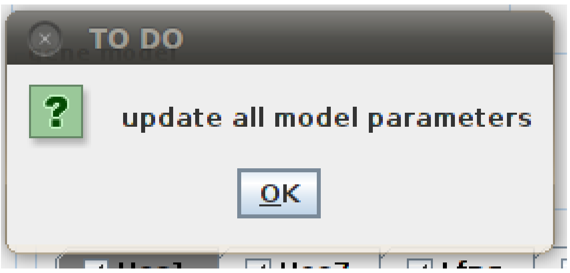

3. The parameter values are updated after clicking the ***OK*** button (see picture below).

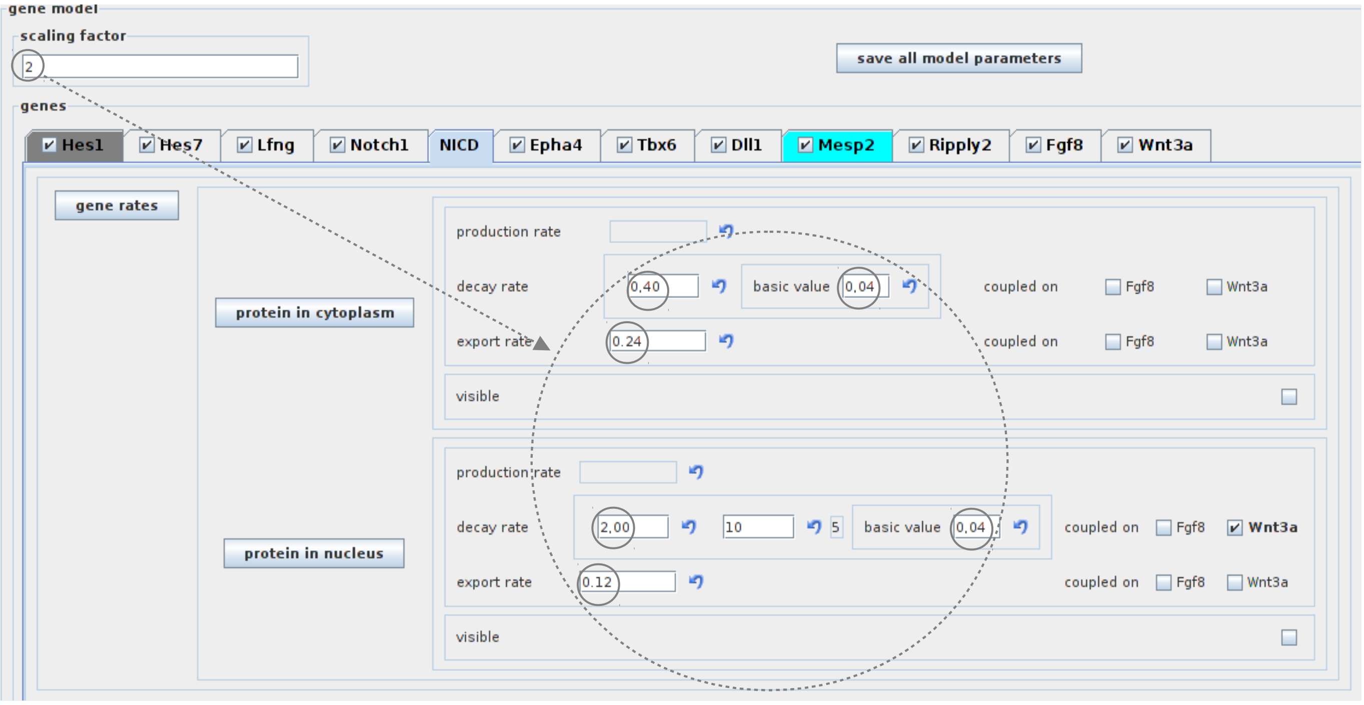

- The '***save all model parameters***' button allows the user to update the configuration file *sim_model/<model_description>/configfile*, i.e. all parameter modifications made on the GUI can be saved to be used by another program run. The old *configfile* ist moved to *OLD/configfile.OLD<index>*. By clicking the button a pop-up window opens with the option to make a notice which will be saved at the beginning of the configuration file (see picture below).

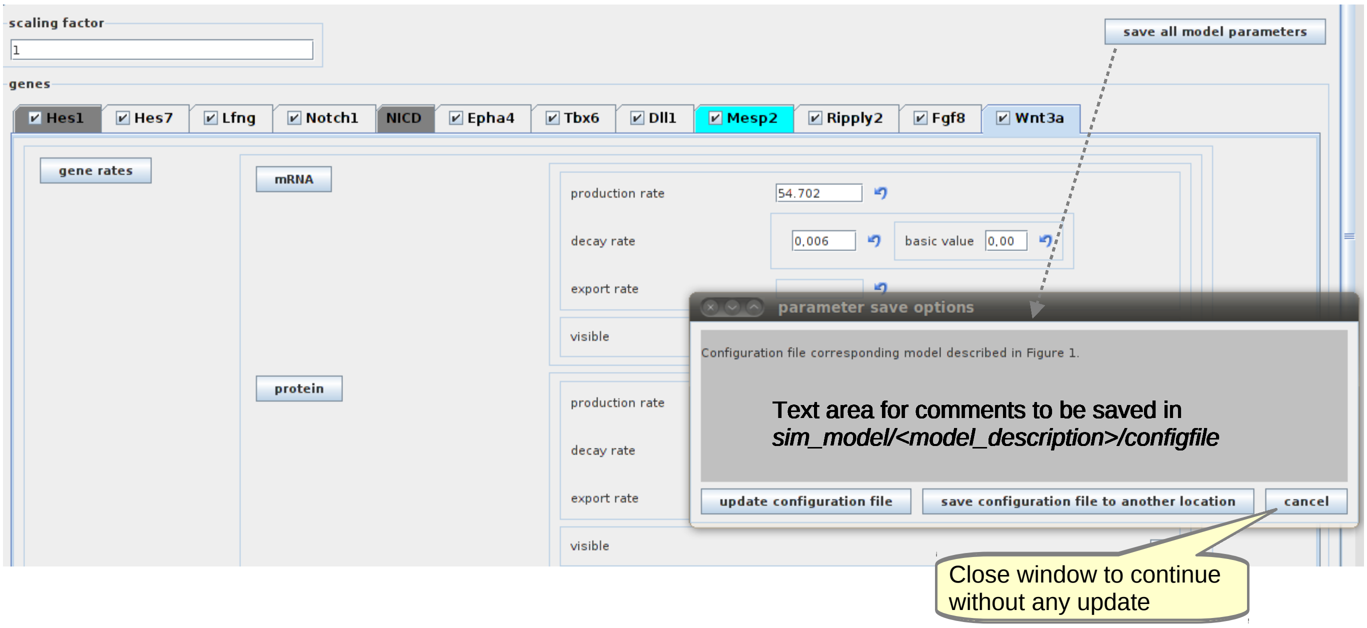

- All **genes** in the GRN are described on panels sharing the same space. The user can view and accordingly modify the parameter values of one specific gene by selecting the tab corresponding to it. The color of the tab indicates which gene products will be shown during the simulation run or whether gradient genes are coupled or not.

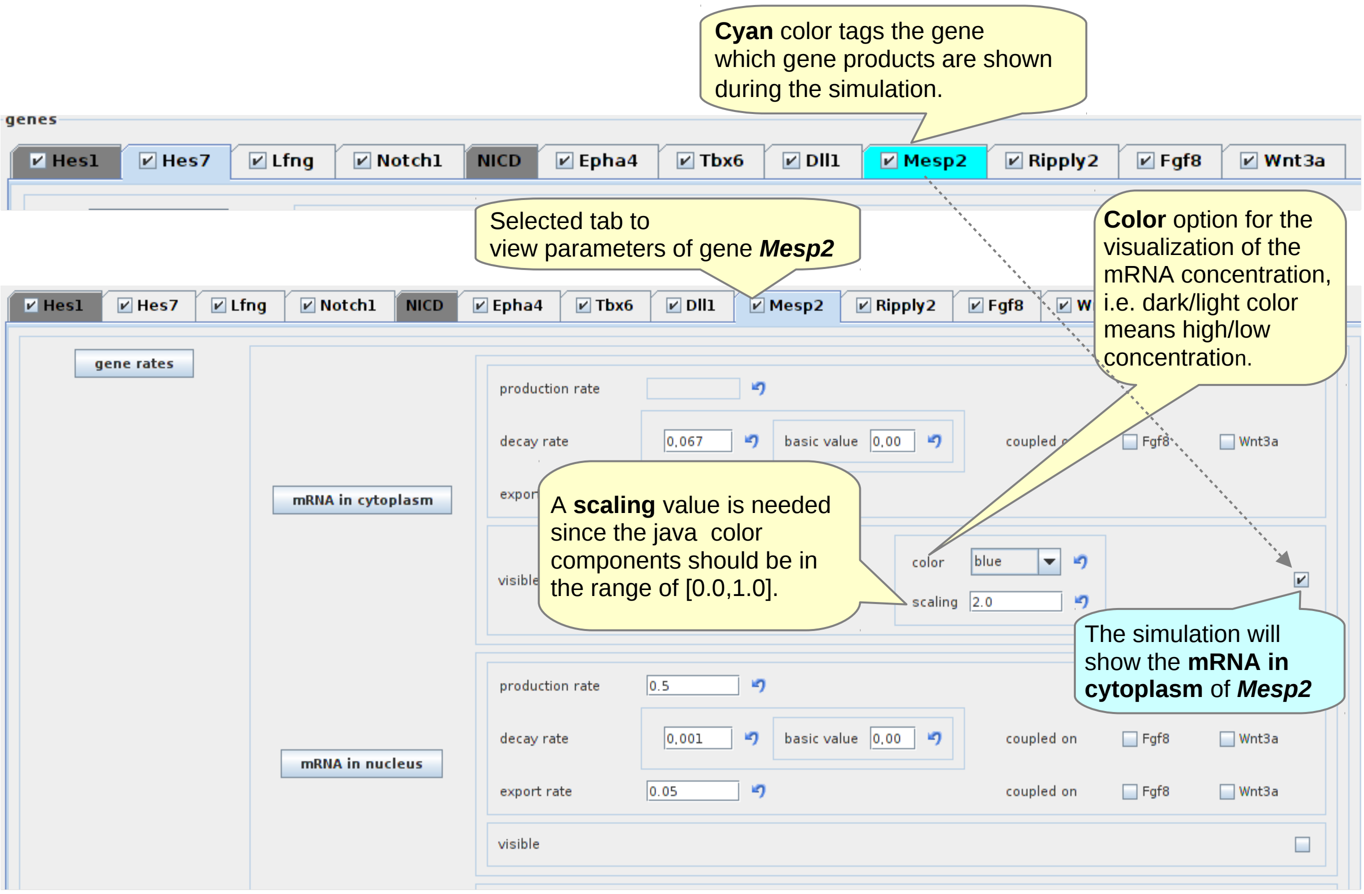

A dark gray colored tab indicates that gradient genes are coupled.

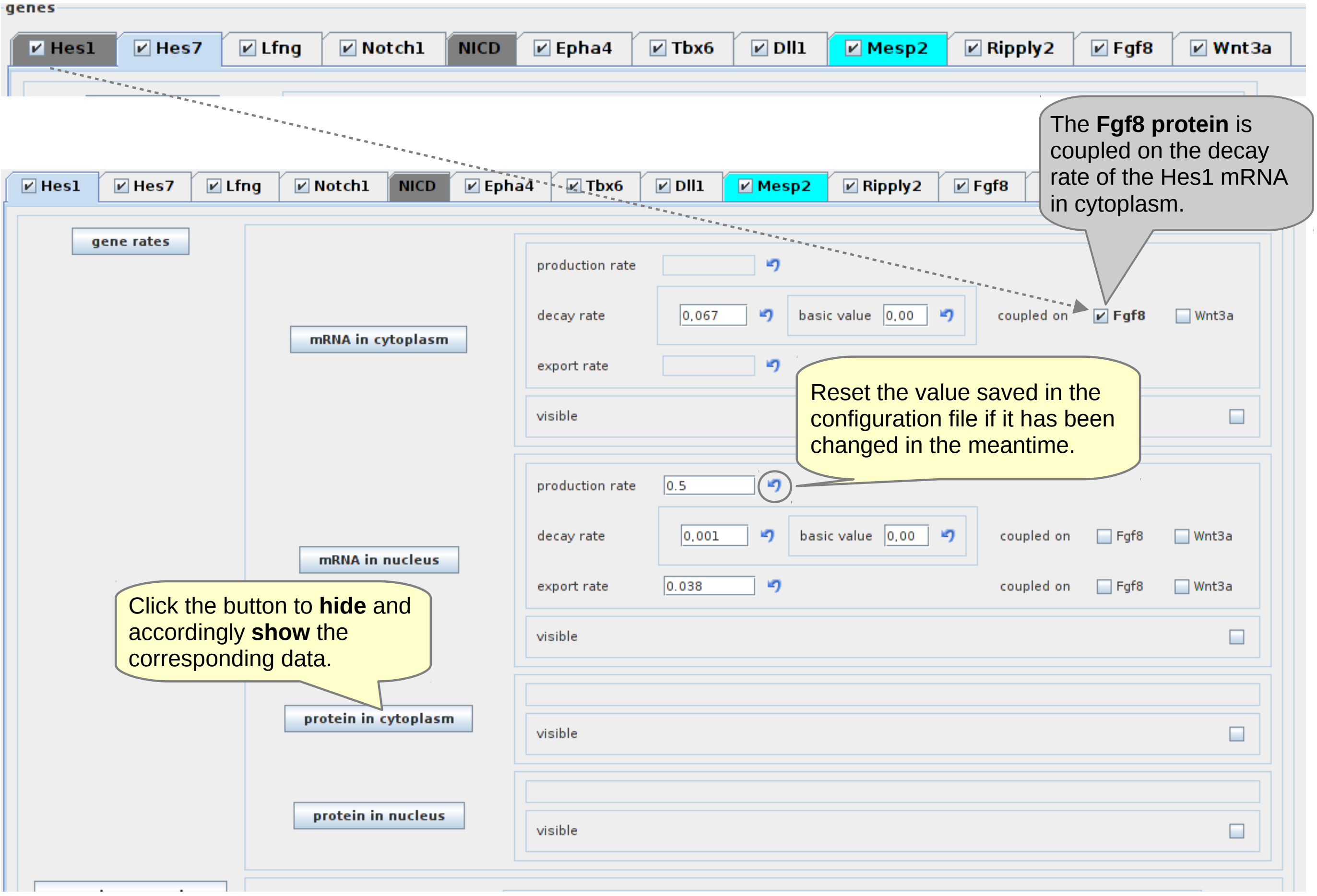

The status of each gene in the GRN can be changed by clicking on the corresponding check box.

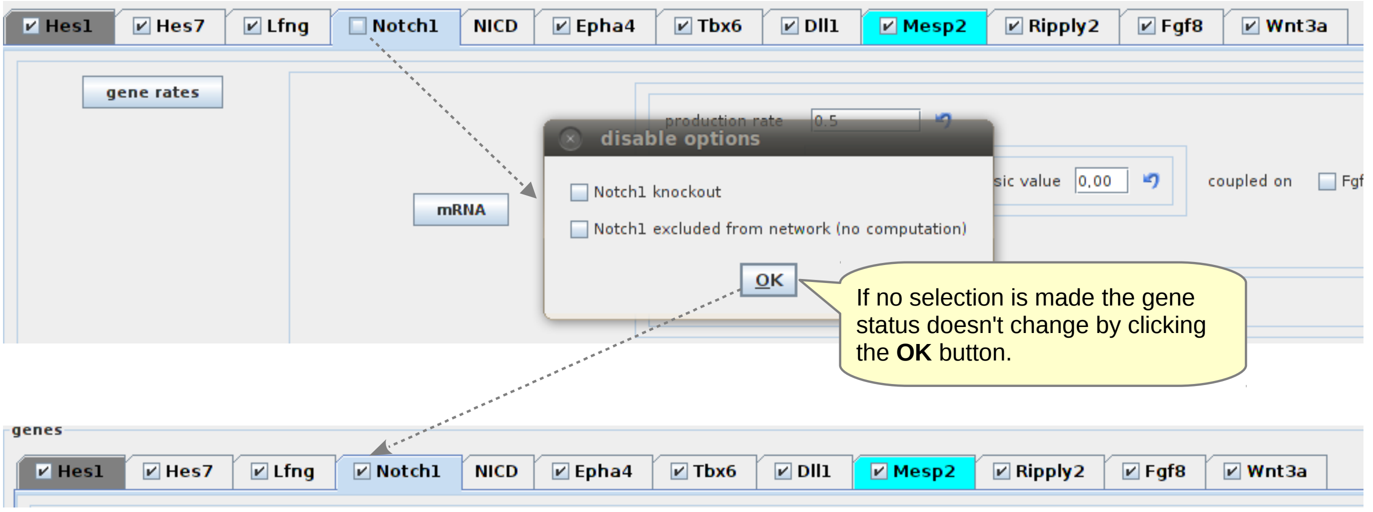

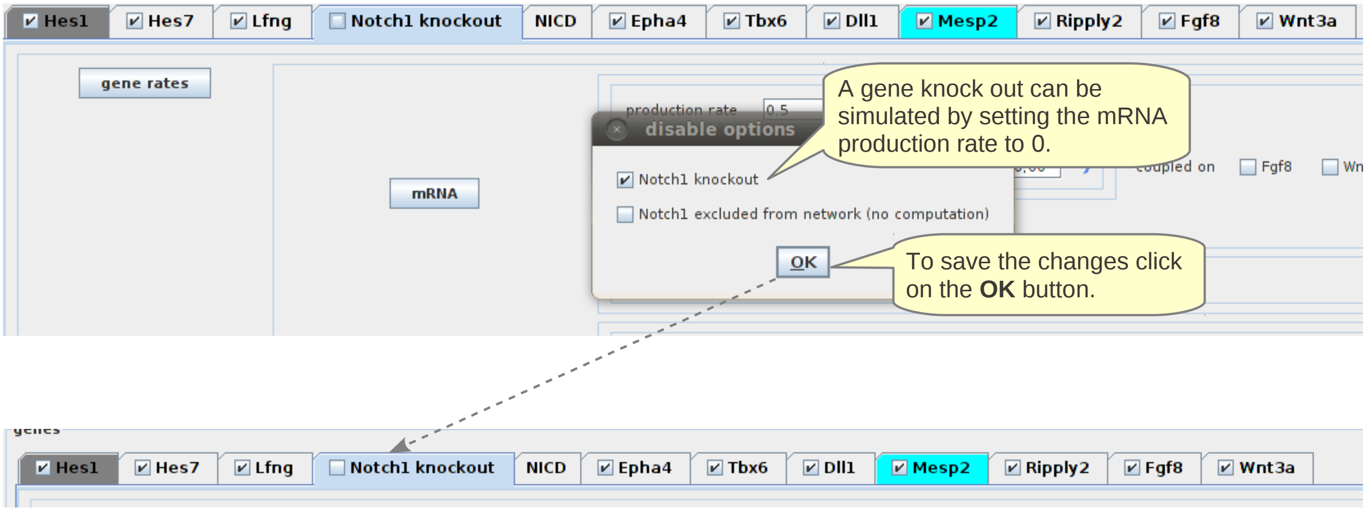

Each gene can also be excluded from the network. This means that no computation will be done for its products. In case of Delta/Notch genes the elimination of ***Dll1*** (***Notch1***) in the GRN implies also the elimination of ***Notch1*** (***Dll1***) and **NICD**.

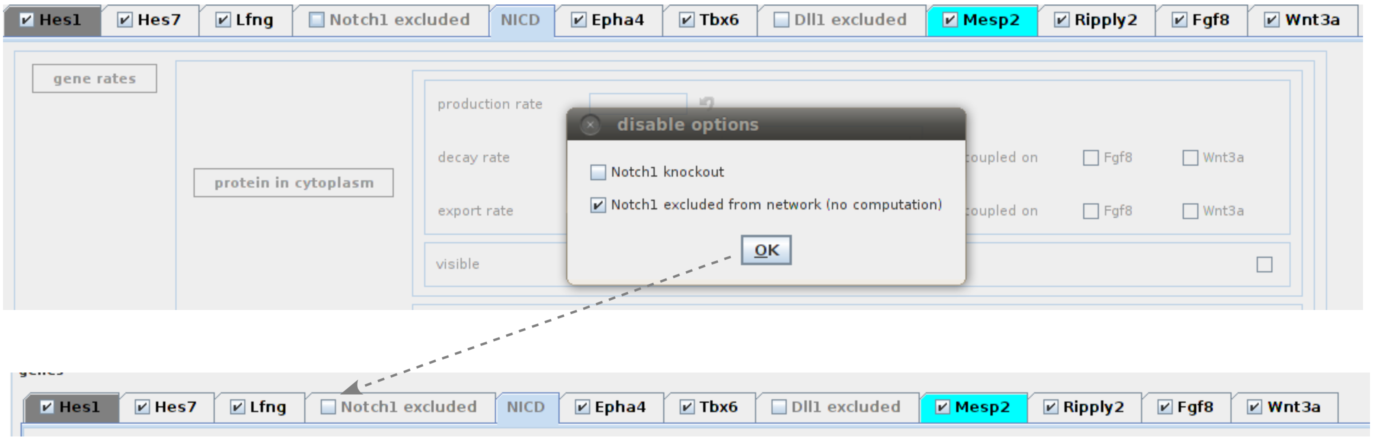

In the case of ***Fgf8*** the option of a *partial inhibition* is also supplied. This means, that the protein production rate can be reduced by a defined amount at a defined time step during the simulation.

The gene panel contains also information about gene promoters.

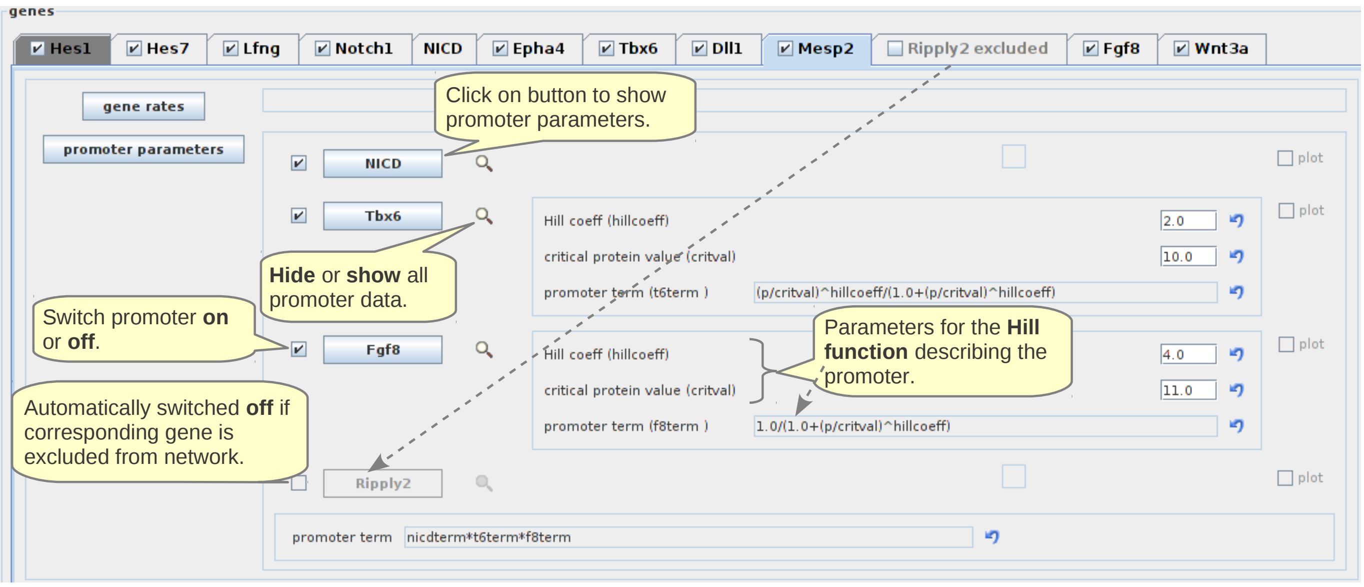

The lower part of the GUI is reserved for general settings like the layout of the proliferating cells and the way they proliferate.

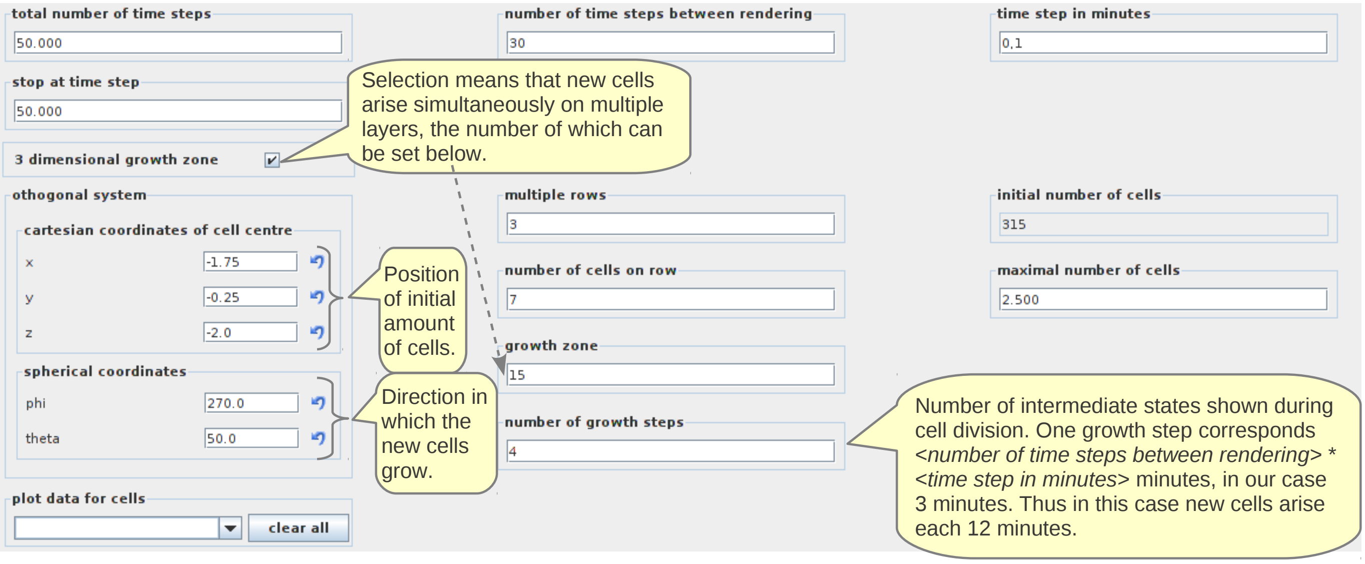

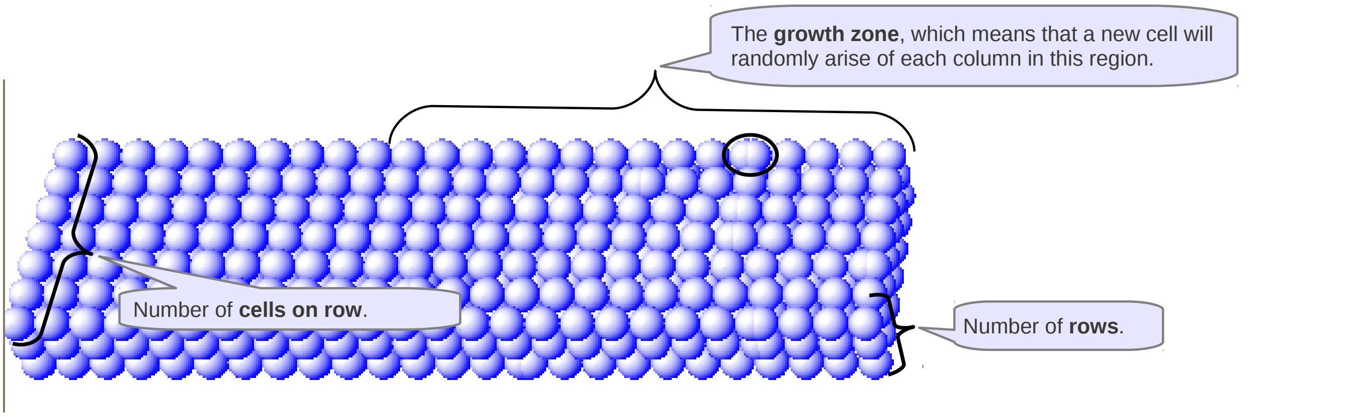

If the '***3 dimensional growth zone***' option is not selected, the growth zone consists of 1 layer, from which all new cells arise.

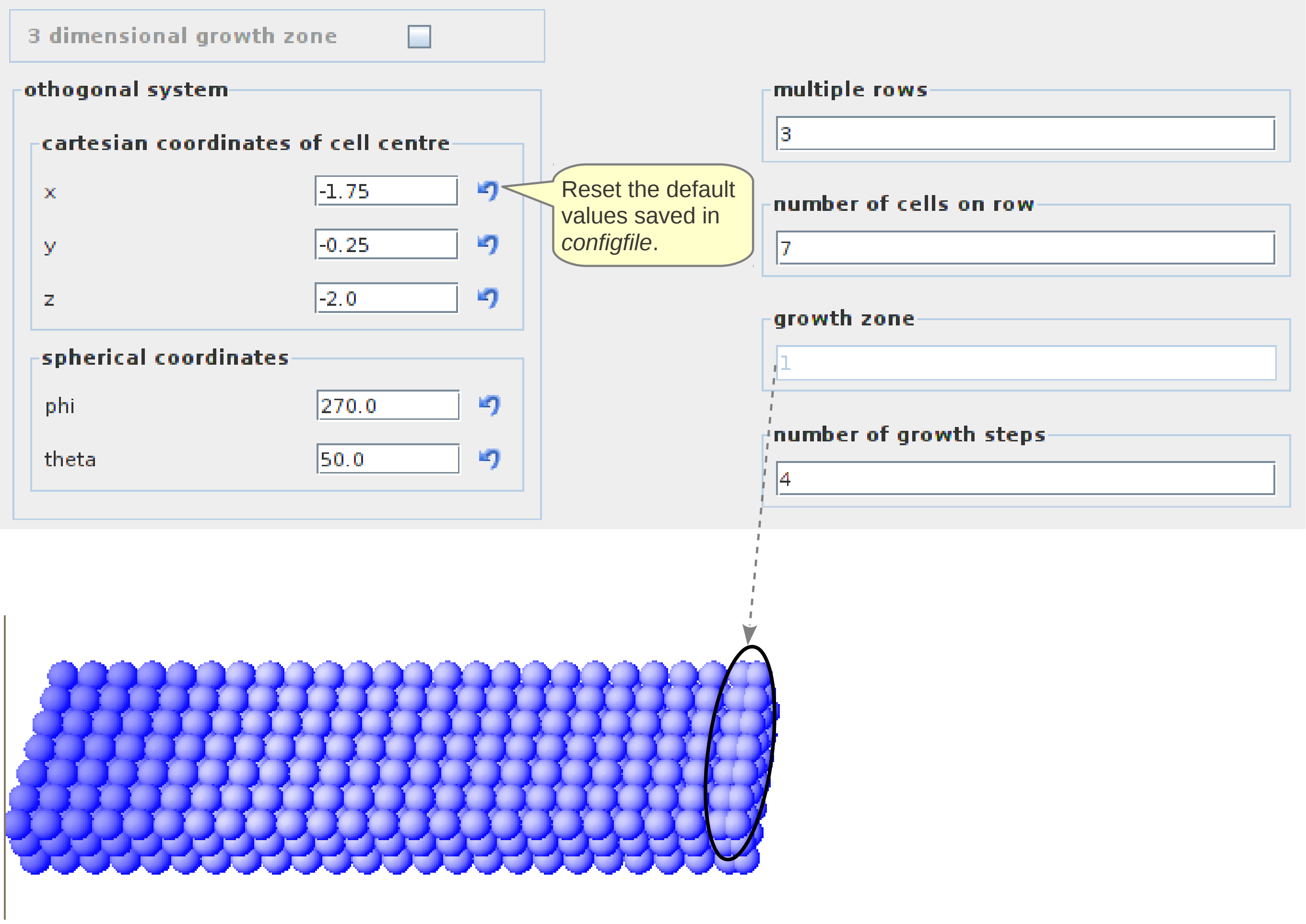

The '***plot data for cells***' field gives users the option to output the oscillators numerical data, which means that after each step of the Runge-Kutta method used to solve the system of differential equations modeling the GRN, the data will be written to a file, which can be read by an appropriate program (like *gnuplot*) to visualize the time course of the concentrations.

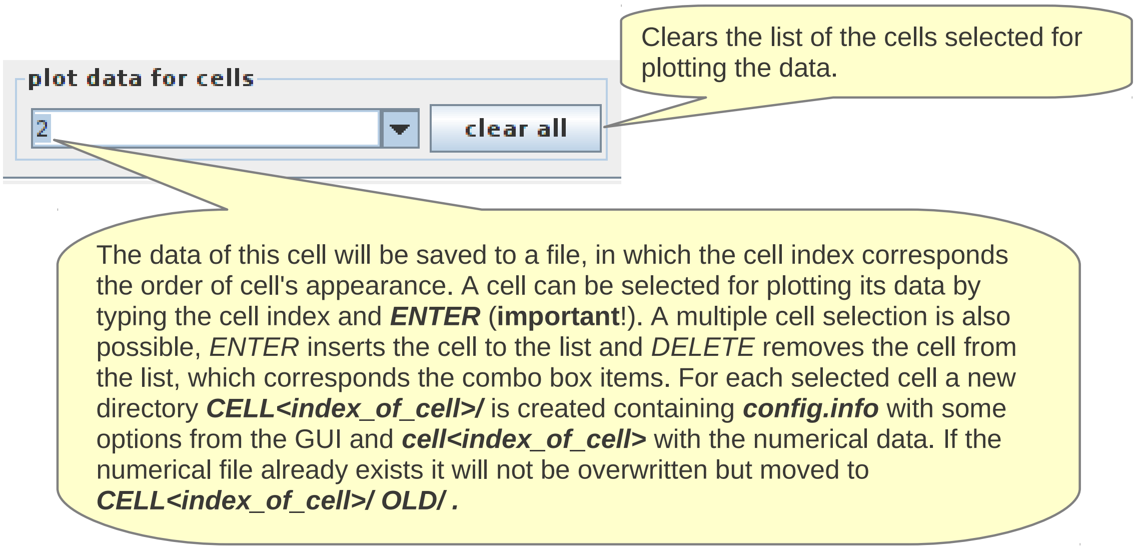

The **simulation** starts by clicking the **'*start*'** button at the bottom of the configuration panel.
